# Mortality, ethnicity, and country of birth on a national scale, 2001–2013: A retrospective cohort (Scottish Health and Ethnicity Linkage Study)

**DOI:** 10.1371/journal.pmed.1002515

**Published:** 2018-03-01

**Authors:** Raj S. Bhopal, Laurence Gruer, Genevieve Cezard, Anne Douglas, Markus F. C. Steiner, Andrew Millard, Duncan Buchanan, S. Vittal Katikireddi, Aziz Sheikh

**Affiliations:** 1 Edinburgh Migration, Ethnicity and Health Research Group, Usher Institute of Population Health Sciences and Informatics, University of Edinburgh, Edinburgh, United Kingdom; 2 Environmental & Occupational Medicine, Section of Population Health, University of Aberdeen, Aberdeen, United Kingdom; 3 NHS Grampian, Aberdeen, United Kingdom; 4 NHS Health Scotland, Glasgow, United Kingdom; 5 Information Services Division, NHS National Services Scotland, Edinburgh, United Kingdom; 6 MRC/CSO Social and Public Health Sciences Unit, University of Glasgow, Glasgow, United Kingdom; Stanford University, UNITED STATES

## Abstract

**Background:**

Migrant and ethnic minority groups are often assumed to have poor health relative to the majority population. Few countries have the capacity to study a key indicator, mortality, by ethnicity and country of birth. We hypothesized at least 10% differences in mortality by ethnic group in Scotland that would not be wholly attenuated by adjustment for socio-economic factors or country of birth.

**Methods and findings:**

We linked the Scottish 2001 Census to mortality data (2001–2013) in 4.62 million people (91% of estimated population), calculating age-adjusted mortality rate ratios (RRs; multiplied by 100 as percentages) with 95% confidence intervals (CIs) for 13 ethnic groups, with the White Scottish group as reference (ethnic group classification follows the Scottish 2001 Census). The Scottish Index of Multiple Deprivation, education status, and household tenure were socio-economic status (SES) confounding variables and born in the UK or Republic of Ireland (UK/RoI) an interacting and confounding variable. Smoking and diabetes data were from a primary care sub-sample (about 53,000 people). Males and females in most minority groups had lower age-adjusted mortality RRs than the White Scottish group. The 95% CIs provided good evidence that the RR was more than 10% lower in the following ethnic groups: Other White British (72.3 [95% CI 64.2, 81.3] in males and 75.2 [68.0, 83.2] in females); Other White (80.8 [72.8, 89.8] in males and 76.2 [68.6, 84.7] in females); Indian (62.6 [51.6, 76.0] in males and 60.7 [50.4, 73.1] in females); Pakistani (66.1 [57.4, 76.2] in males and 73.8 [63.7, 85.5] in females); Bangladeshi males (50.7 [32.5, 79.1]); Caribbean females (57.5 [38.5, 85.9]); and Chinese (52.2 [43.7, 62.5] in males and 65.8 [55.3, 78.2] in females). The differences were diminished but not eliminated after adjusting for UK/RoI birth and SES variables. A mortality advantage was evident in all 12 minority groups for those born abroad, but in only 6/12 male groups and 5/12 female groups of those born in the UK/RoI. In the primary care sub-sample, after adjustment for age, UK/RoI born, SES, smoking, and diabetes, the RR was not lower in Indian males (114.7 [95% CI 78.3, 167.9]) and Pakistani females (103.9 [73.9, 145.9]) than in White Scottish males and females, respectively. The main limitations were the inability to include deaths abroad and the small number of deaths in some ethnic minority groups, especially for people born in the UK/RoI.

**Conclusions:**

There was relatively low mortality for many ethnic minority groups compared to the White Scottish majority. The mortality advantage was less clear in UK/RoI-born minority group offspring than in immigrants. These differences need explaining, and health-related behaviours seem important. Similar analyses are required internationally to fulfil agreed goals for monitoring, understanding, and improving health in ethnically diverse societies and to apply to health policy, especially on health inequalities and inequities.

## Abstract 2

Using data from the Scottish Health and Ethnicity Linkage Study, Raj Bhopal and colleagues examine the associations between ethnicity, country of birth, and mortality.

## Author summary

**Why was this study done?:**

- There is an important question of whether ethnic minority populations have worse or better health than the majority population of the country they live in.
- The limited international evidence on this question, including from the UK, is unclear.

**What did the researchers do and find?:**

- Using death rates, an internationally accepted measure of population health, the researchers compared the health of ethnic groups in Scotland.
- They did this by anonymously linking Scottish death records for 4.62 million people for 12 years (2001–2013) to the Scotland census for 2001, thus creating a retrospective cohort study. They included almost the whole Scottish population and used the ethnic group categories that individuals had provided in the census.
- Both males and females in most ethnic minority groups had relatively lower death rates than the majority White Scottish population, even when the older average age of the latter was accounted for. The Chinese ethnic group had the lowest death rates.
- Members of ethnic groups born outside the UK or Republic of Ireland generally had comparatively low mortality compared to the White Scottish majority, but this pattern was not clear cut in those born within the UK or Republic of Ireland.

**What do these findings mean?:**

- This study shows that, as measured by death rates, ethnic minority groups in Scotland have relatively good health. Exactly why this is remains unclear and probably has many reasons. By more detailed studies, e.g., of the Chinese ethnic group, we may be able to find ways of improving the health of the whole population.
- Policy, research, and public health practice in relation to inequalities, inequities, and the social determinants of health should consider incorporating an ethnic health perspective.

## Introduction

Migrant and ethnic minority groups in European countries are typically perceived as vulnerable populations, with poor health relative to the majority. Yet, the health indicator all-cause mortality may be low for some ethnic minority groups [1,2]. For example, the UK’s Parliamentary Office of Science and Technology said, “Black and minority ethnic groups generally have worse health than the overall population, which is mainly attributed to their poorer socio-economic position” [3]. Such statements are, seemingly, not based on comprehensive or general indicators [2,4].

Mortality rates in migrants from low- to high-income countries are sometimes below those of the host population despite their usually lower or similar socio-economic status (SES) [2,5]. This finding, sometimes referred to as the healthy migrant effect, is clearer in migrants from distant than nearby countries. The main hypotheses for the healthy migrant effect and a summary of the literature are considered in the Discussion and have been reviewed recently [2,5,6]. Data from the UK provide complex and sometimes contradictory findings. For example, Marmot et al. analysed mortality around the 1971 census in England and Wales for immigrants from Indian subcontinent countries combined and reported a standardised mortality ratio (SMR) (reference 100) of 98 in men and 106 in women [7]. For those born in the Indian subcontinent, Wild and McKeigue reported an SMR of 106 in men and 100 in women around the 1991 census [8]. By contrast, Fischbacher et al.’s study in Scotland around the 2001 census disaggregated the group as Indian, Pakistani, and Bangladeshi, and found lower SMRs in all except Indian females, e.g., the SMR for Pakistani-born males was 62.9, and when standardised to England and Wales as the reference it was 82.4 [9]. Clearly, the matter requires further study, including assessing whether the patterns by country of birth found by Fischbacher et al. could be corroborated and were also seen by ethnic group.

This study is in Scotland, which has lower life expectancy and higher age-specific mortality than England and Wales and many other European countries for complex and incompletely understood reasons, with time trends indicating slower improvement in mortality than elsewhere from the 1950s onwards [10]. Sizeable inequalities are demonstrable between regions, cities, and socio-economic groups, with the West of Scotland, where most of Scotland’s ethnic minority populations live, having the poorest health [11,12]. Amongst explanations that have been scrutinised are health-related behaviours, SES, deindustrialisation, and damage to social cohesion through housing and other policies [10,12,13]. Examining mortality by ethnic group and country of birth adds potentially important new perspectives to previous analysis.

Understanding mortality by ethnic group and country of birth in multiethnic societies is core to understanding health status, which underpins health and healthcare needs assessment [14]. This information is needed to address inequality and inequity in demographically changing populations to help refine healthcare strategy and health policy.

The capacity to investigate this phenomenon cross-nationally either in Europe [1,4] or globally is limited as the international Human Mortality Database, covering 37 countries, does not include either ethnicity or country of birth, excepting Māori and non-Māori groups in New Zealand [15]. The UN’s Sustainable Development Goal 17.18 calls for reliable data including by race, ethnicity, and migratory status by 2030, but this has rarely been achieved, even in the most developed countries. Most mortality studies in migrants and ethnic minorities in Europe have used country of birth, but this approach does not include the offspring of migrants in the minority groups [4,16], though parental country of birth is sometimes used for children [17]. Furthermore, some people born abroad belong to the majority ethnic group of their country of residence. Studying mortality by both ethnicity and country of birth is desirable [14,18].

Scotland has, historically, been a country with high levels of emigration but is now becoming a multiethnic country as a result of immigration, and the offspring of immigrants, e.g., non-White ethnic groups, have increased from 1% of the population in 1991 to 2% in 2001 and about 4% in 2011, with the rise continuing. The Scottish Health and Ethnicity Linkage Study (SHELS) is a response by the Scottish government and subsequently the Chief Scientist Office to a scarcity of health statistics by ethnic group, permitting analysis by both ethnic group and country of birth [19]. Our primary, prior hypothesis for this analysis was one that underpinned most analysis of outcomes in phase 4 of SHELS, i.e., that there are at least 10% differences, in this case, in age-adjusted mortality rate ratios (RRs) between ethnic minority groups and the White Scottish majority. Given the limitations of the datasets, it would be challenging to study smaller differences accurately and precisely. Although the hypothesis is non-directional, we had prior indication of the likely directions from earlier, smaller scale work using country of birth by Fischbacher et al., where the main findings were higher mortality in men born in the Republic of Ireland and lower mortality in most other groups of men including those born in Pakistan, Bangladesh, China, and Hong Kong [9]. We hypothesized that differences would be attenuated but remain after adjusting for country of birth and SES. We also examined mortality by comparing those born in the UK or Republic of Ireland (UK/RoI) with those born elsewhere. In a sub-cohort [20], we explored the possible roles of smoking and diabetes in ethnic variations in mortality. Trend analysis was done to provide indirect evidence on remigration (salmon) bias [21].

## Methods

### Ethical and other approvals

The Multi-centre Research Ethics Committee for Scotland, the Privacy Advisory Committee of NHS National Services Scotland, and general practice data custodians gave approval. We have had an assessment of our procedures by an ethicist and a public panel [22,23]. Researchers with appropriate training (GC, MFCS) analysed data in a safe room at National Records of Scotland. Outputs were reviewed by the National Records of Scotland Disclosure Committee.

The background and methods are published in journals [19,24] and on the SHELS website (http://www.ed.ac.uk/usher/scottish-health-ethnicity-linkage), and more details are in S1 Appendix. The work was guided by our grant application and an analysis plan that was signed off before analysis started (S1 Protocol; https://www.ed.ac.uk/files/atoms/files/data_analysis_plan_for_shels_4_0.pdf), with no specific registration or protocol. In reporting we followed the RECORD statement (S1 Checklist).

SHELS used probability matching of names, sex, address, and date of birth to link the Scottish 2001 Census to the Community Health Index (CHI) of people registered with the Scottish National Health Service (NHS) (Section 1 and Fig A of S1 Appendix). The algorithms were developed by staff at National Records of Scotland. Where linkage fails, the reasons for non-linkage have been considered [19,20,25]. Using the CHI number, we linked the census to NHS data including mortality records previously transferred from National Records of Scotland. About 95% (approximately 4.62 million) of 4.86 million people in the census were linked, 91% of the estimated whole population of 5.1 million. Ten general practices with a relatively high proportion of ethnic minority patients agreed for their primary care data, including on smoking and diabetes, to be linked to our databases [20]. The sub-cohort comprised 52,975 people, of whom 48,325 (91.2%) had smoking status recorded and 2,900 (5.5%) had a diagnosis of diabetes. Given the small population, this sub-cohort analysis was primarily a demonstration of the potential value of including risk factors from primary care, and here we compare only 3 minority groups in men and 2 in women.

The UK has developed an ethnic group classification through consultation and field testing that has evolved since the 1970s (though only first used in 1991) [14,26]. We followed the terminology of the Scottish 2001 Census, including capitalisation of group labels [14]. In 2001, the variable sex was collected (male or female). Respondents selected 1 of 14 categories in the ethnic group question (Table A of S1 Appendix). We excluded the Other ethnic group as too heterogeneous for research purposes. Census data included country of birth and SES based on the Scottish Index of Multiple Deprivation (derived from postcodes and small areas), highest educational qualification in 3 categories (individual level for people aged 16–74 years and highest household educational level outside this age range), and housing tenure at household level (owning or renting).

### Analysis

Our data analysis plan (https://www.ed.ac.uk/files/atoms/files/data_analysis_plan_for_shels_4_0.pdf) is provided as S1 Protocol. In our data analysis plan, the work on all-cause mortality is discussed on page 25, and the additional work using primary care data is discussed on page 56. For the main analysis, i.e., mortality by ethnic group by sex, adjusted for a number of appropriate variables, we followed the plan exactly, with minor deviations. The plan states that specific age group data and main causes of death will be explored if numbers (sample sizes), time, and resources allow. The investigators agreed that the numbers were insufficient for such analysis, especially given that many cause-specific results have already been published from SHELS by combining mortality and hospitalisation data. The plan included an analysis by country of birth, for comparison with Fischbacher et al.’s results [9], and this is in Table H of S1 Appendix. We focused on additional analysis by ethnic group and by whether people were born within the UK/RoI or not. The planned trend analysis for each 3- to 4-year period was replaced with a similar analysis using 3-year moving averages. The analysis using the primary care data was done exactly as in the plan.

The disclosure protocol of National Records of Scotland required that for outputs the numbers were rounded to the nearest 5 and percentages estimated from these (the analysis used exact numbers). For deaths at all ages between 1 May 2001 and 30 April 2013, using Poisson regression with robust variance, we calculated the following by sex and ethnic group: (a) the denominator as person-years (PY) censored for death or transfer of records from the NHS in Scotland to elsewhere in the UK; (b) age-adjusted mortality rates per 100,000 PY; (c) age-adjusted mortality RRs, with White Scottish as the reference group (primary analysis), then further adjusted for SES and country of birth; (d) age-adjusted mortality RRs further adjusted for smoking and diabetes in the primary care sub-cohort; and (e) 95% confidence intervals (CIs) for summary measures.

As is the convention in SHELS (Appendix E of S1 Protocol), we multiplied age-adjusted mortality RRs by 100 to be interpretable as percentages, as for a SMR. We focused on age-adjusted mortality RRs where the ratio differed by at least 10%, in line with our hypothesis, and used the 95% CIs to examine the precision of the differences. When the change in age-adjusted mortality RR following adjustment for SES or country of birth was 10% or more (absolute not relative measure), we regarded that as potentially important.

Country of birth was categorised as either born in or outside the UK/RoI, and this was included in the analysis as an interaction with ethnicity, stratified by sex, with White Scottish born in UK/RoI as the reference (Table F of S1 Appendix). On the presumption of convergence in mortality across time and generations, we expected differences in age-adjusted mortality RRs to diminish after adjustment and to reflect what the patterns would be if all groups had the country-of-birth pattern of the reference population.

Using our published method [24], we examined whether the associations between SES indicators and outcome were similar across ethnic groups before using the SES indicators as confounding variables. We calculated 3-year moving averages for age-adjusted mortality RRs by ethnicity: if unrecorded emigration was relatively high in minority groups, their age-adjusted mortality RRs would spuriously reduce over time, as deaths abroad are not recorded (the alternative of genuinely reducing relative mortality is implausible given published trends in risk factors and outcomes [27,28]). More details on calculating person-years and Poisson regression modelling are given in Section 3 of S1 Appendix. Sample size calculations were not done. Data were analysed using SAS version 9.4 (SAS Institute; Cary, North Carolina, US).

## Results

### Background information on the linked cohort

As shown in linkage by ethnic group in Table A of S1 Appendix, and in the characteristics of the study population in Table B of S1 Appendix, in 2001 there were relatively large Other White British, White Irish, and Other White minority populations in Scotland, but fewer than 10,000 males or 10,000 females in non-white groups except for the Pakistani group (about 2% of total population). The mean ages of non-white groups were lower than that of the White Scottish group (38 years), especially that of the Any Mixed Background group (21 years). Many non-white people were UK/RoI born, e.g., 60% of Pakistani females. SES was highest for the Other White British group and varied for non-white ethnic groups, partly dependent on the indicator and sex. In males, as SES diminished, mortality rose in all ethnic groups (Table C of S1 Appendix). In females, the association of mortality with highest educational qualification was as expected in all ethnic groups except for the Chinese ethnic group. We judged the associations were consistent enough to treat the 3 SES variables as valid measures of potential confounders.

### Mortality by ethnic group and sex, adjusting for confounders

**Fig 1:**
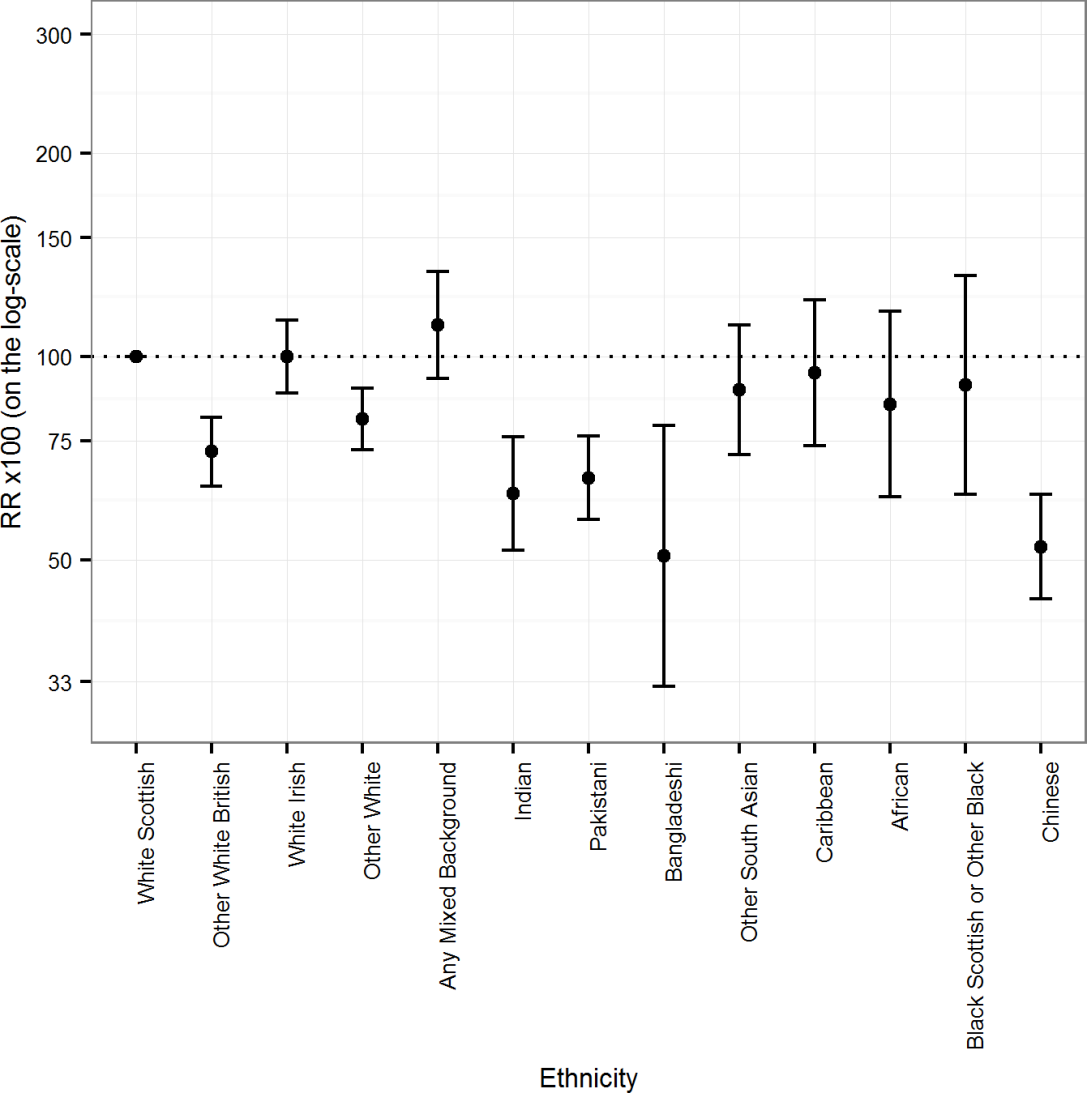
Age-adjusted rate ratios (RRs) (bars show 95% CIs) for all-cause mortality by ethnicity in males.

Table 1 shows that in males in some ethnic groups, the numbers of deaths were small and the 95% CIs were wide, e.g., 20 deaths in the Bangladeshi group (age-adjusted mortality RR 50.7 [95% CI 32.5, 79.1]). Fig 1 and Table 1 show that age-adjusted mortality RRs were mostly below 100, with a 10% or greater difference from the White Scottish majority for the Other White British, Other White, Indian, Pakistani, Bangladeshi, and Chinese groups. White Irish, Caribbean, and Black Scottish or Other Black males had smaller than 10% differences from the reference group. The only group with a 10% or higher age-adjusted mortality RR than reference was Any Mixed Background (111.3 [95% CI 98.7, 139.3]). The 95% CIs provided evidence that the RR was more than 10% lower in the following male groups: Other White British (72.3 [95% CI 64.2, 81.3]); Other White (80.8 [72.8, 89.8]); Indian (62.6 [51.6, 76.0]); Pakistani (66.1 [57.4, 76.2]); Bangladeshi (50.7 [32.5, 79.1]); and Chinese (52.2 [43.7, 62.5]).

**Table 1:** Age-adjusted mortality rates per 100,000 PY and RRs for all-cause mortality by ethnic group in males.

Ethnic group	Number of deaths	PY at risk	Mortality Rate	RR (95% CI) by adjustment variables			
				Age	Age and UK/RoI born	Age and SES	Age, UK/RoI born, and SES
White Scottish	200,725	21,179,755	947.7	100.0	100.0	100.0	100.0
Other White British	12,975	1,571,080	684.8	72.3 (64.2, 81.3)	72.6 (64.5, 81.8)	85.9 (82.0, 90.1)	86.1 (82.2, 90.3)
White Irish	2,710	202,190	946.5	99.9 (88.2, 113.1)	99.9 (88.2, 113.2)	94.9 (89.3, 100.9)	94.9 (89.3, 100.9)
Other White	1,930	278,515	765.9	80.8 (72.8, 89.8)	91.7 (83.1, 101.2)	87.7 (82.0, 93.7)	92.9 (86.7, 99.5)
Any Mixed Background	195	56,265	1,055.2	111.3 (92.8, 133.5)	117.2 (98.7, 139.3)	106.8 (92.5, 123.4)	109.1 (94.5, 125.9)
Indian	265	65,945	593.3	62.6 (51.6, 76.0)	72.8 (60.4, 87.9)	77.9 (67.2, 90.3)	83.3 (71.5, 96.9)
Pakistani	435	146,430	626.5	66.1 (57.4, 76.2)	76.6 (66.7, 88.0)	71.2 (63.7, 79.6)	76.2 (67.7, 85.7)
Bangladeshi	20	8,700	480.2	50.7 (32.5, 79.1)	57.7 (37.0, 89.9)	53.9 (36.0, 80.6)	57.0 (38.1, 85.3)
Other South Asian	115	26,800	845.9	89.3 (71.6, 111.3)	101.3 (81.8, 125.3)	90.7 (75.7, 108.7)	95.7 (79.7, 114.9)
Caribbean	45	7,245	896.9	94.6 (73.8, 121.3)	107.0 (83.8, 136.7)	96.4 (76.6, 121.2)	101.6 (80.6, 128.0)
African	60	20,135	806.0	85.0 (62.0, 116.7)	98.1 (71.7, 134.2)	82.1 (61.0, 110.6)	87.6 (65.0, 118.0)
Black Scottish or Other Black	25	4,780	859.7	90.7 (62.4, 131.9)	95.8 (66.1, 138.8)	80.1 (54.9, 116.8)	82.0 (56.2, 119.6)
Chinese	195	68,685	495.0	52.2 (43.7, 62.5)	60.9 (51.0, 72.6)	53.8 (46.2, 62.7)	57.6 (49.2, 67.5)

RRs are adjusted for age, UK/RoI born, and SES (Scottish Index of Multiple Deprivation, household tenure, educational attainment).

CI, confidence interval; PY, person-years; RR, rate ratio; SES, socio-economic status; UK/RoI, United Kingdom/Republic of Ireland.

Table 1 also shows that further adjustment for being born in the UK/RoI increased, mostly by 10% or more, the age-adjusted mortality RRs for all minority groups, except for White Irish, Other White British, Any Mixed Background, and Black Scottish or Other Black; in these 4 groups most people were born in the UK/RoI. Adjustment for SES variables had inconsistent effects: in some groups the age-adjusted mortality RR increased towards the reference value, sometimes by more than 10% (Other White British and Indian ethnic groups), and in others the changes were small (White Irish, Any Mixed Background, and African ethnic groups). But in the Black Scottish or Other Black group, the age-adjusted mortality RR reduced by more than 10%. Adjustment for age, country of birth, and SES mostly shifted the mortality RRs towards the reference value, sometimes considerably so, e.g., for the Other White British and Indian ethnic groups. The exceptions were the White Irish and Black Scottish or Other Black groups. Even after full adjustment, the age-adjusted mortality RRs of several groups (Other White British, Other White, Indian, Pakistani, Bangladeshi, African, and Black Scottish or Other Black) remained relatively low, usually with the 95% CIs being quite narrow and excluding the reference value. For example, the age-adjusted mortality RR of Chinese males remained notably low after such adjustment (57.6 [95% CI 49.2, 67.5]).

**Fig 2:**
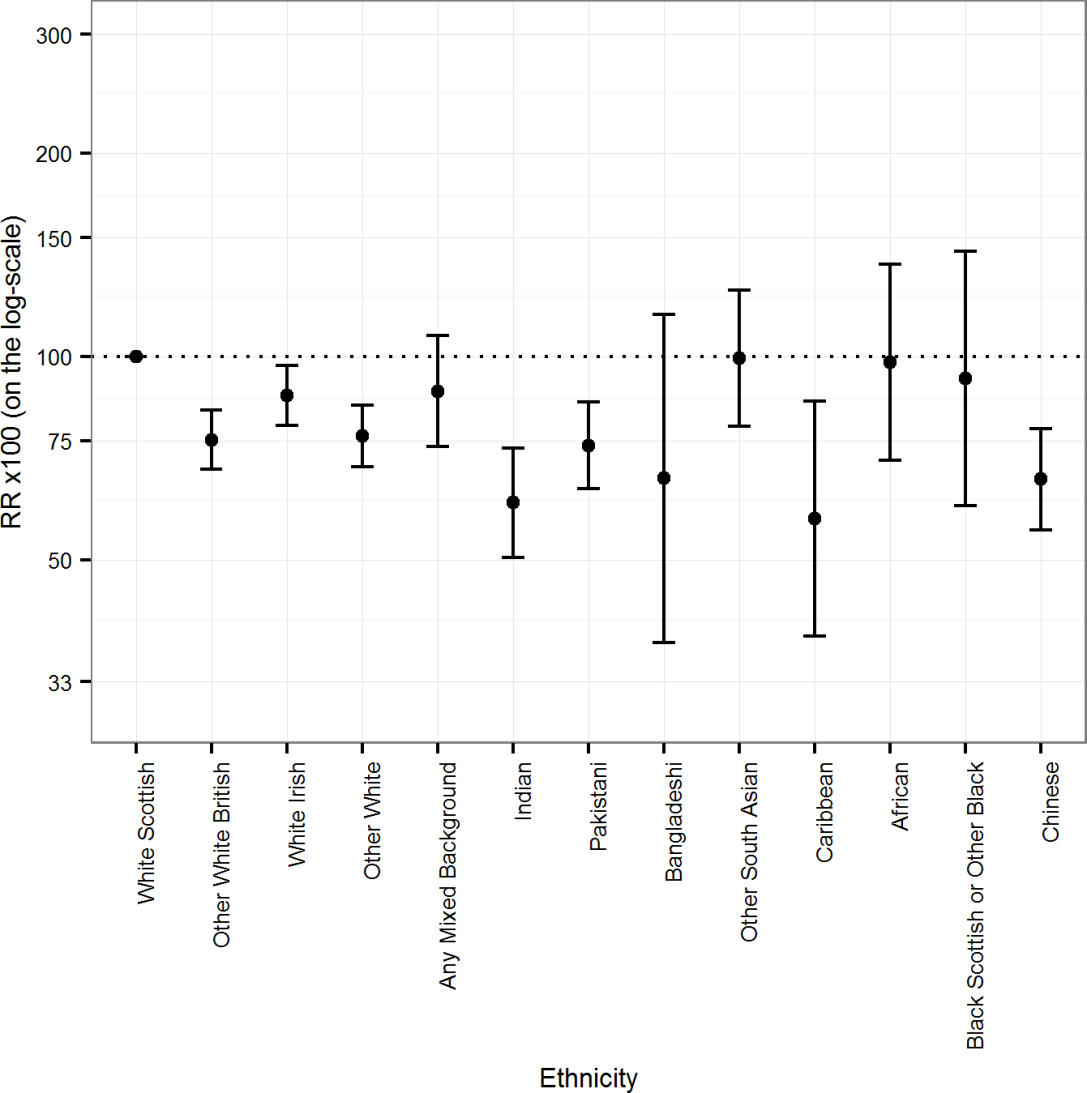
Age-adjusted rate ratios (RRs) (bars show 95% CIs) for all-cause mortality by ethnicity in females.

Fig 2 and Table 2 show similar results for females as for males. The age-adjusted mortality RRs were more than 10% lower than the reference group except for the Other South Asian, African, and Black Scottish or Other Black groups. The 95% CIs provided good evidence that the RR was more than 10% lower than reference in the following female groups: Other White British (75.2 [95% CI 68.0, 83.2]); Other White (76.2 [68.6, 84.7]); Indian (60.7 [50.4, 73.1]); Pakistani (73.8 [63.7, 85.5]); Caribbean (57.5 [38.5, 85.9]); and Chinese (65.8 [55.3, 78.2]).

**Table 2:** Age-adjusted mortality rates per 100,000 PY and RRs for all-cause mortality by ethnic group in females.

Ethnic group	Number of deaths	PY at risk	Mortality Rate	RR (95% CI) by adjustment variables			
				Age	Age and UK/RoI born	Age and SES	Age, UK/RoI born, and SES
White Scottish	179,955	22,581,190	796.9	100.0	100.0	100.0	100.0
Other White British	11,155	1,644,435	599.5	75.2 (68.0, 83.2)	75.8 (68.5, 83.8)	86.4 (82.7, 90.2)	86.7 (83.0, 90.6)
White Irish	2,470	216,905	697.5	87.5 (79.1, 96.9)	87.5 (79.0, 96.8)	86.8 (80.7, 93.4)	86.8 (80.7, 93.4)
Other White	1,735	319,915	607.4	76.2 (68.6, 84.7)	89.8 (81.6, 98.8)	84.5 (79.6, 89.8)	93.1 (87.3, 99.3)
Any Mixed Background	145	59,970	708.0	88.8 (73.5, 107.4)	94.2 (78.5, 113.1)	82.7 (70.1, 97.5)	85.3 (72.4, 100.5)
Indian	155	59,925	483.5	60.7 (50.4, 73.1)	72.6 (60.5, 87.3)	72.3 (61.1, 85.7)	80.4 (67.7, 95.5)
Pakistani	295	143,940	588.1	73.8 (63.7, 85.5)	88.3 (76.4, 102.2)	80.7 (71.1, 91.6)	89.8 (78.6, 102.5)
Bangladeshi	15	6,910	525.7	66.0 (37.7, 115.4)	77.5 (44.9, 133.7)	65.4 (36.3, 117.8)	71.9 (40.2, 128.5)
Other South Asian	75	21,700	791.7	99.3 (78.8, 125.3)	112.3 (89.6, 140.8)	95.9 (77.8, 118.0)	102.8 (83.5, 126.5)
Caribbean	25	7,825	458.5	57.5 (38.5, 85.9)	65.5 (44.3, 96.7)	65.9 (45.6, 95.2)	70.8 (49.2, 101.9)
African	40	15,865	781.6	98.1 (70.2, 137.1)	114.4 (82.2, 159.3)	95.5 (69.6, 131.2)	104.7 (76.1, 144.0)
Black Scottish or Other Black	25	4,900	738.7	92.7 (60.1, 143.0)	98.7 (64.7, 150.4)	84.3 (56.0, 126.9)	87.4 (58.6, 130.6)
Chinese	175	68,010	524.0	65.8 (55.3, 78.2)	80.0 (67.4, 94.9)	70.1 (59.9, 82.1)	78.7 (66.9, 92.5)

RRs are adjusted for age, UK/RoI born, and SES (Scottish Index of Multiple Deprivation, household tenure, educational attainment).

CI, confidence interval; PY, person-years; RR, rate ratio; SES, socio-economic status; UK/RoI, United Kingdom/Republic of Ireland.

In females, as in males, age-adjusted mortality RRs rose after adjustment for being born in the UK/RoI, except for the Other White British and White Irish groups. In Other South Asian and African females, the age-adjusted mortality RRs after adjustment for UK/RoI born exceeded 100 though the 95% CIs were wide and included the reference value. There were variable effects of adjustment for SES variables. In Other White British, Other White, Indian, Pakistani, and Caribbean females, the age-adjusted mortality RRs rose by more than 10%. In some groups, they declined but not by more than 10% (White Irish, Any Mixed Background, Other South Asian, African, and Black Scottish or Other Black ethnic groups). After full adjustment, the age-adjusted mortality RRs rose substantially in several groups (e.g., Other White British, Other White, Indian, Pakistani, and Caribbean ethnic groups). There were declines in fully adjusted age-adjusted mortality RRs in the White Irish, Any Mixed Background, and Black Scottish or Other Black ethnic groups, though they were less than 10%, and the 95% CIs were wide.

### Moving average analysis

The 3-year moving average age-adjusted mortality RRs were stable in the larger ethnic groups. In the smaller groups, there was considerable 3-year-to-3-year variation, but no apparent trend (Table D of S1 Appendix).

### Linkage to primary care data

**Fig 3:**
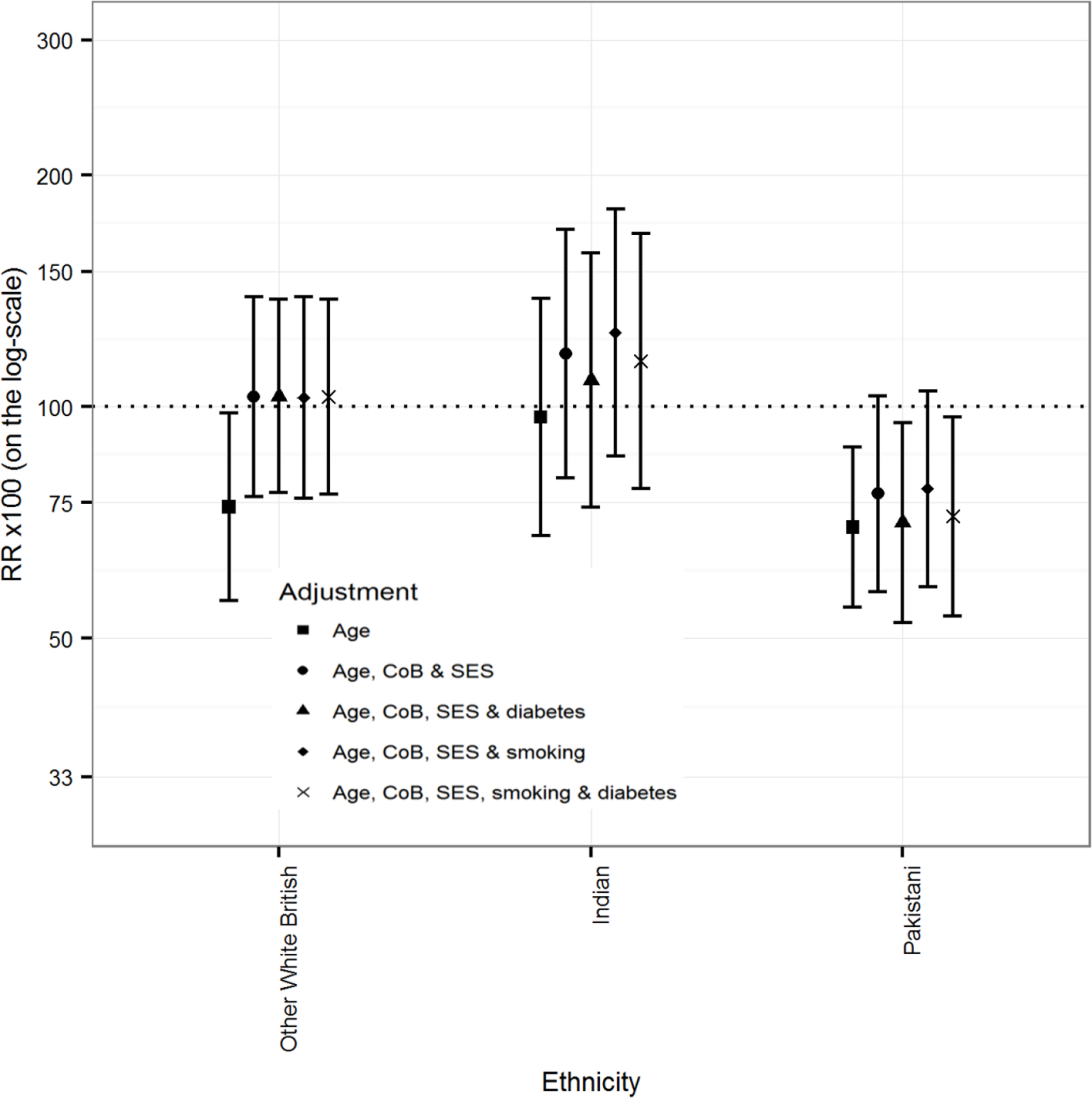
Primary care sub-cohort data: Rate ratios (RRs) (bars shows 95% CIs) for all-cause mortality by ethnicity adjusted for age, CoB relating to UK/RoI born or not, socio-economic status (SES), smoking status, and diabetes in males.

**Fig 4:**
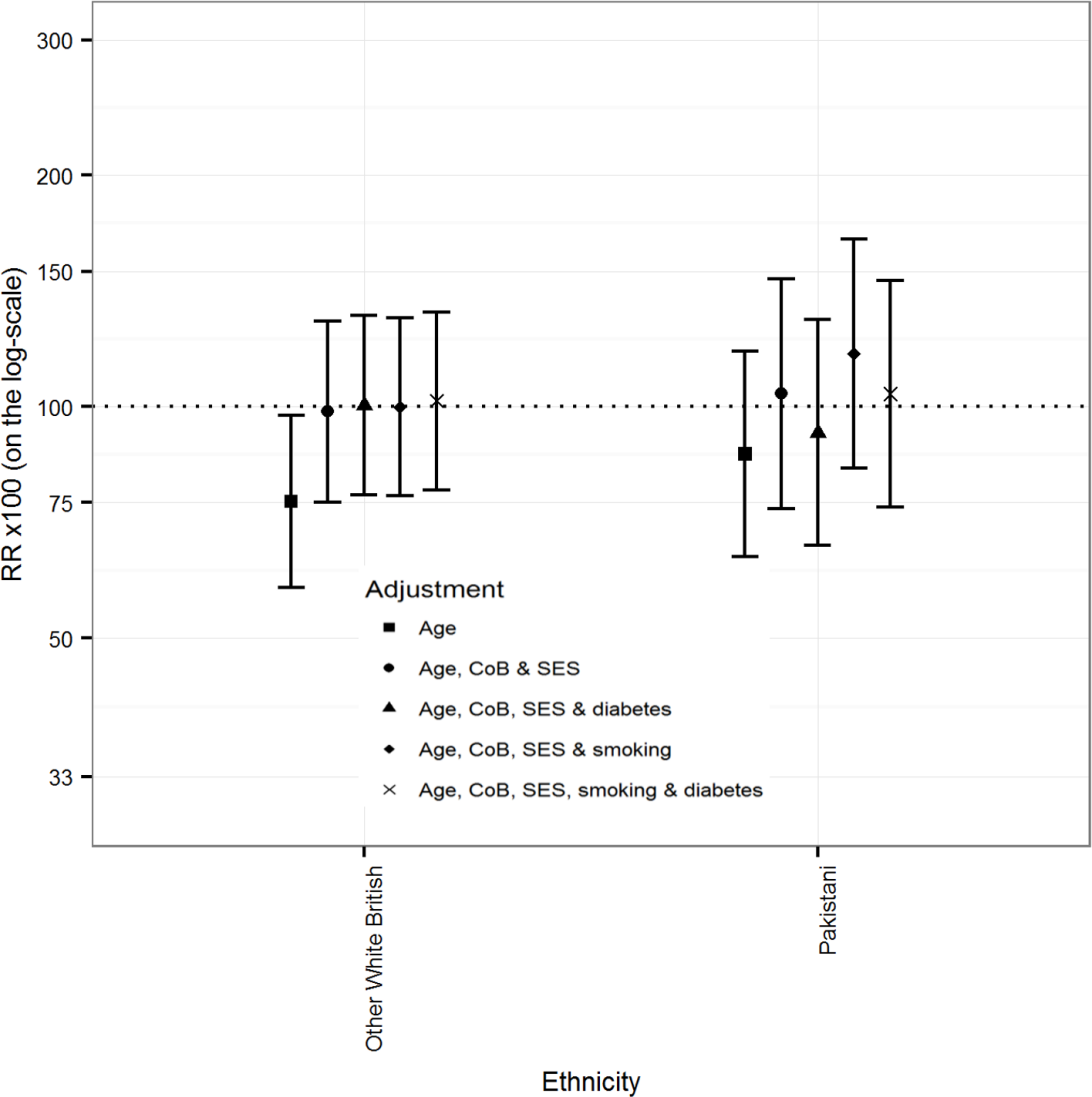
Primary care sub-cohort data: Rate ratios (RRs) (bars shows 95% CIs) for all-cause mortality by ethnicity adjusted for age, CoB relating to UK/RoI born or not, socio-economic status (SES), smoking status, and diabetes in females.

Fig 3 (males) and Fig 4 (females) show that the differences between White Scottish (dotted line) and Other White British males and females disappeared after adjusting for country of birth and SES variables, with little further change after adjustment for smoking and diabetes. In Indian males, adjusting for country of birth, SES variables, diabetes, and smoking status raised the age-adjusted mortality RR from 96.9 (95% CI 67.9, 138.3) to 114.7 (95% CI 78.3, 167.9). (There were too few outcomes for Indian females for an interpretable analysis.) Similar adjustments made little change in age-adjusted mortality RRs in Pakistani males (from 69.7 [95% CI 54.8, 88.6] to 72.0 [53.4, 97.0]). Adjusting further for diabetes made little difference in either Indian or Pakistani males. In Pakistani females, adjusting for country of birth, SES variables, and smoking raised the age-adjusted mortality RR from 86.8 (95% CI 63.8, 118.0) to 117.2 (83.2, 165.1). Adding diabetes changed the RR towards the reference value (103.9 [95% CI 73.9, 145.9]. (The data for Figs 3 and 4 are in Table E of S1 Appendix.)

### Comparing those born in and outside the UK/RoI

**Fig 5:**
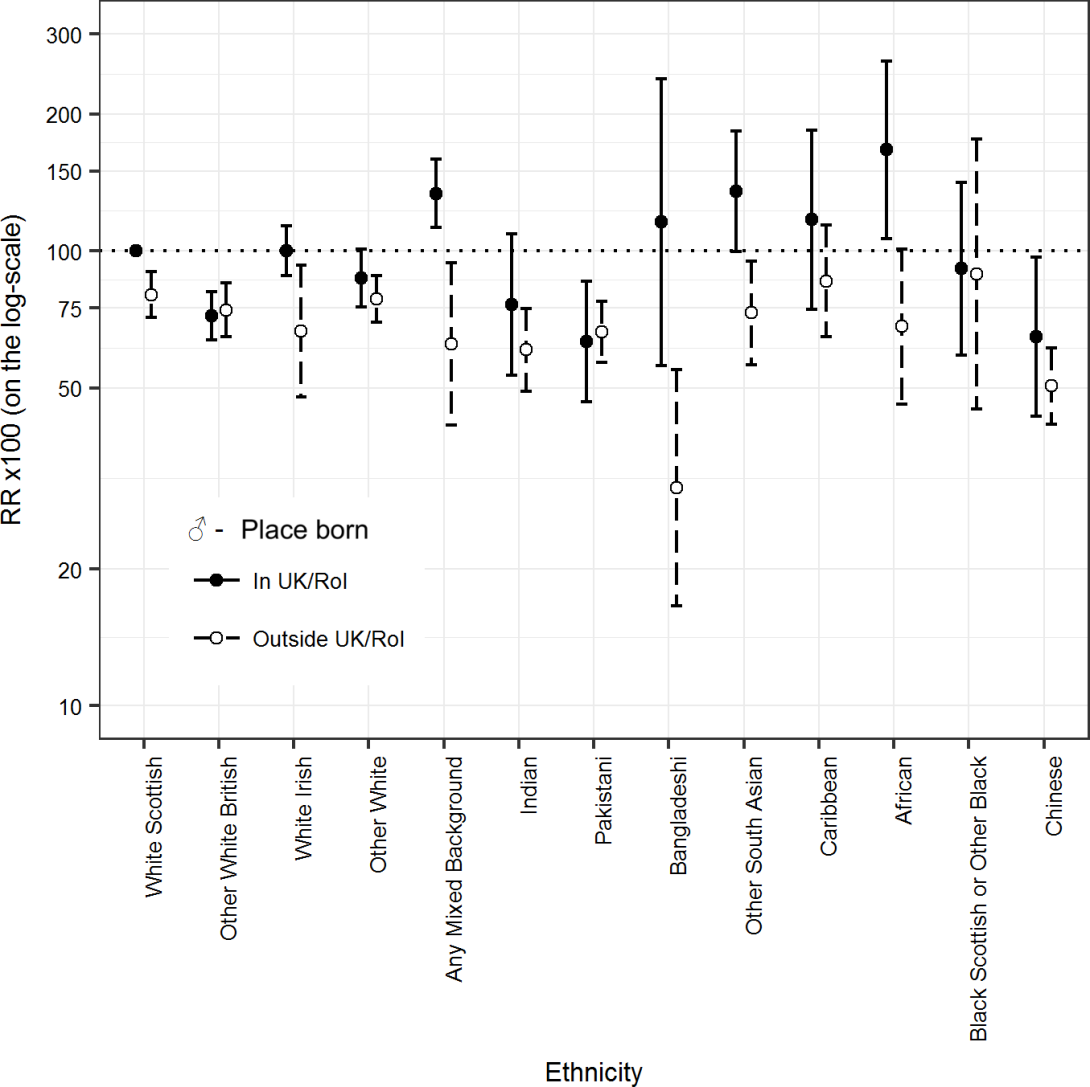
Age-adjusted RRs (bars show 95% CIs) for all-cause mortality by ethnicity and country of birth (UK/RoI born or not) in males. RR, rate ratio; UK/RoI, United Kingdom or Republic of Ireland.

**Fig 6:**
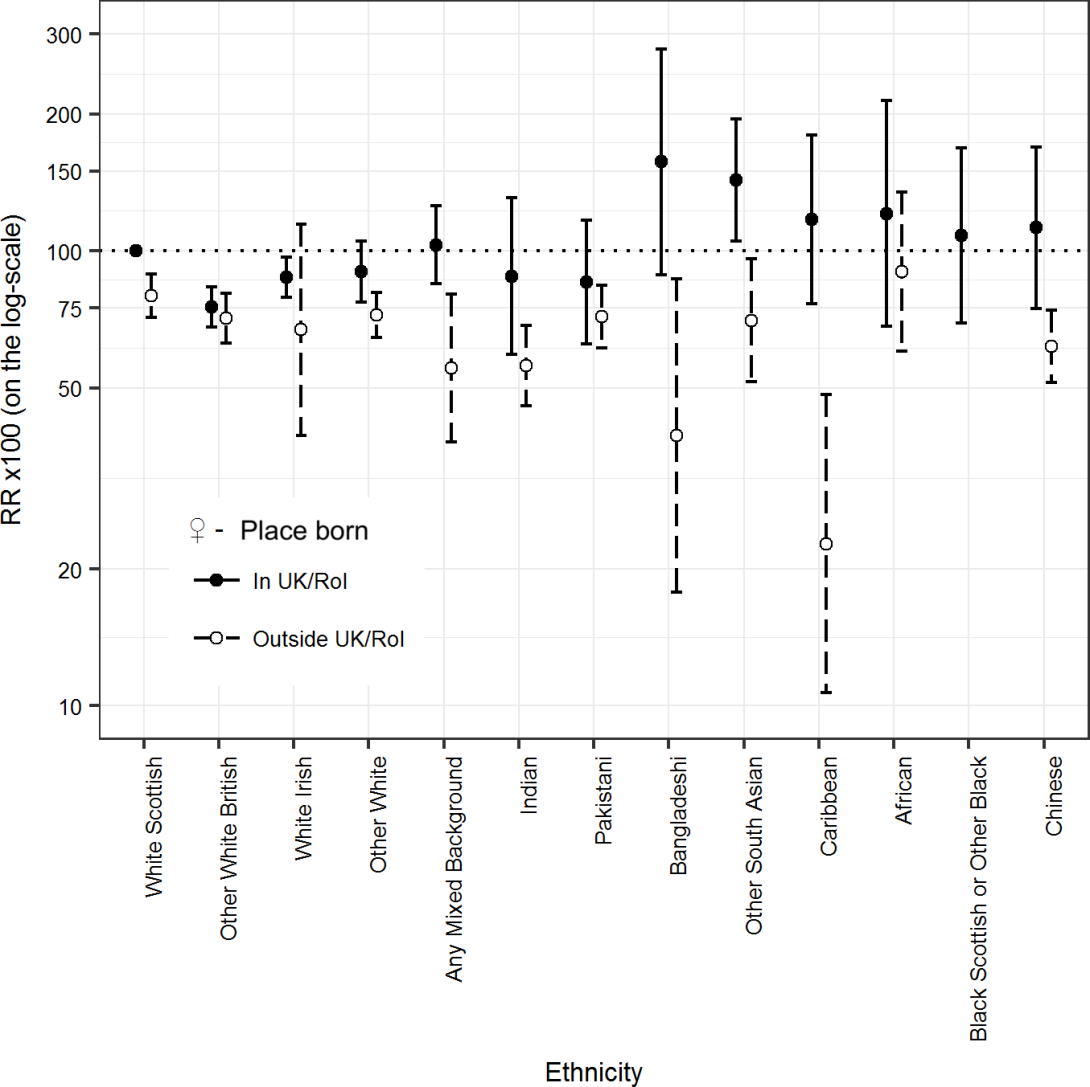
Age-adjusted RRs (bars show 95% CIs) for all-cause mortality by ethnicity and country of birth (UK/RoI born or not) in females. RR, rate ratio; UK/RoI, United Kingdom or Republic of Ireland.

Fig 5 (males) and Fig 6 (females) show that for all ethnic groups, individuals born outside the UK/RoI, including those in the White Scottish group, had lower age-adjusted mortality RRs than the reference group (White Scottish born in the UK/RoI), with 95% CIs mostly below 100. For those born in the UK/RoI, the pattern was inconsistent: 6/12 male minority groups and 5/12 female minority groups had lower age-adjusted mortality RRs than the reference group. (The data for the figures are in Table F of S1 Appendix.) For males in the Any Mixed Background group, age-adjusted RR was markedly lower in those born outside the UK/RoI (62.5 [95% CI 41.4, 94.3] than in those born in UK/RoI (133.9 [112.7, 158.9]), and results were similar in the male African group, with a RR of 68.2 (46.1, 100.8) in those born outside the UK/RoI and 166.8 (106.3, 261.9) in those born in UK/RoI.

## Discussion

In Scotland, between 2001 and 2013, several ethnic minority groups, and especially non-white populations, had substantially lower mortality—a key indicator of health status—than the White Scottish majority. Our hypothesis was mostly upheld as mortality rates were relatively low in many ethnic minority groups in Scotland, often lower than that of the White Scottish group by more than 10% [9]. Adjusting for country of birth mostly attenuated associations. Members of ethnic minority groups born outside the UK/RoI had mortality rates consistently lower than the White Scottish reference, with inconsistent patterns in those born in the UK/RoI. Adjusting for SES showed varying effects, reflecting the differing socio-economic profiles of the ethnic groups relative to the White Scottish group, but differences were not abolished. After adjustment for smoking in a primary care sub-cohort, Indian men and Pakistani women had no mortality advantage (though 95% CIs were wide), reflecting the higher smoking rates in the White Scottish population.

Our study adds to the methods of the field and previous evidence of a health advantage among migrant and ethnic minority groups, albeit in Scotland, a country with high mortality compared to others of similar SES [10]. This study demonstrated the potential value of primary care data linkage on a national scale [20]. Our methods could be utilised in other countries to augment currently sparse data.

Migrants’ mortality advantage is a paradox given the link between health status, adverse socio-economic circumstances, and suboptimal access to health care, from which we would anticipate lower mortality in majority populations, which are usually advantaged (though not always so in Scotland) [2,14].

There are 4 main hypotheses for the mortality paradox, all potentially relevant depending on context and populations [2,14,18]. First, migrants might be selected for better health compared to their counterparts who remain in the country of origin, especially if they migrate long distances. This has been studied closely, particularly in US Hispanic populations [18,29,30]. Second, migrants might have relatively healthy lifestyles and behaviours, e.g., lower smoking rates, which combine with better socio-economic and environmental conditions in their destination country to produce good health [31,32]. This advantage should diminish with acculturation. Third, the lower mortality could be a data artefact, with underestimation of the numerator or overestimation of the denominator in minority groups [14,33]. Fourth, it might be due to unrecorded remigration and deaths abroad, so-called salmon bias [21,34]. Our findings contribute to this literature as considered below, with special emphasis on studies in the UK.

Scotland has high mortality rates compared with similar nations [10], including England, as reflected in Scottish immigrants in England and Wales [8,18,35,36]. A study of mortality by country of birth in 6 European countries including Scotland [37] showed variation by country of birth, country of residence, and outcome. People born in Asia had low mortality, similar to Fischbacher et al. [9] and our current results for Chinese, Indian, Pakistani, and Other South Asian groups. In England and Wales, no consistent mortality advantage was found for South Asian groups in unlinked national mortality analyses around the censuses of 1971 [7], 1981 [35], and 1991 [8]. Chinese populations, included only in the 2001 analysis, had a clear advantage [36]. A mortality advantage was demonstrated in South Asian and Chinese groups in the linked Office for National Statistics (ONS) Longitudinal Study, comprising 1% of the 1971 census population of England and Wales, using both country of birth and ethnic groups, though in 1 study the mortality advantage was clear only after adjusting for socio-economic indicators [18,38]. Wallace reported no advantage in England and Wales for people born in Ireland and Scotland, and for their descendants [18]. The ONS Longitudinal Study indicated convergence across generations [18]. Our observations of smaller and less consistent mortality advantages among members of ethnic minority groups born in the UK/RoI compared with those born outside the UK/RoI, and some convergence in mortality RRs after adjusting for country of birth, support this. The lower mortality of several ethnic minority groups in Scotland, especially those born outside the UK/RoI, may reflect the relatively poor health of the White Scottish population [10,12].

The potential mortality disadvantage among Indian males and Pakistani females after adjusting for smoking echoes results in the US in Hispanic populations [31]. The Health Survey for England 2004 (with similar results in 1999) showed that smoking prevalence is comparatively low in Chinese and Indian populations, and in Pakistani and Bangladeshi females, though not in Pakistani and Bangladeshi males [39]. The Chinese populations in the UK, US, and elsewhere tend to have favourable lifestyles and behaviours, including low smoking rates (unlike Chinese males in China) and low alcohol consumption [39]. Lifestyle as an explanation for mortality differences needs closer study via multiethnic health surveys like the Health Survey for England [39] done internationally, including in Scotland, as well as more extensive linkage to primary care datasets [20].

In England and Wales, relative inequalities by country of birth for cardiovascular disease increased during 1979–2003, as the decline in cardiovascular disease in groups born abroad was not as fast as for those born in England and Wales [27]. In several ethnic minority groups in Scotland, chronic disease rates have overshot those in the White Scottish population, e.g., for coronary heart disease [40]. Fortunately, the results here, together with other publications, show this is not adversely affecting all-cause mortality [9,41], partly due to relatively low cancer rates [25]. Reus-Pons et al. reported similar findings in Belgium [42], with differences partly explained by SES, and the evidence on specific causes of death pointing to lifestyle.

The strengths of our study include high linkage rates, data by both ethnic group and country of birth (used both to stratify and adjust in analyses) as reported by individuals or the householder in the census, checks on the stability of ratios over time, the availability of 3 socio-economic variables, medically certified deaths, and sub-cohort data on smoking and diabetes. In comparison with studies of unlinked mortality and population data, our study reduced the likelihood of numerator/denominator bias [14,33]. Weaknesses include limited risk-factor data, and then only on a small sub-cohort; few deaths in some non-white populations, e.g., the Bangladeshi and Caribbean groups, leading to wide confidence intervals; linkage rates and participation in the census varying by ethnic group (although both were high); and an inability to include deaths abroad. We could not provide direct evidence on remigration bias. Seriously ill people would be likely to stay in Scotland, given that it provides healthcare free at the point of use. This was indirectly supported by our observation of reasonably stable moving averages in mortality RRs. A Danish study of a linked cohort of immigrants found that those with severe chronic disease were less likely to leave Denmark than those without [21]. Analysis by cause was not possible given the small numbers of deaths for most ethnic minority groups (but many outcomes have been reported by SHELS by combining hospitalisations and deaths) [19]. Data from the census are self-reported, thus have limitations including validity, especially when reported across ethnic groups [43]. Our emphasis was on differences of more than 10% in the age-adjusted RR, in line with our prior hypothesis. This decision led to emphases similar to alternative specifications of the size of difference of interest in the range of 5%–20% (Table G of S1 Appendix).

Our results contrast with the harmful perception that the health of immigrants and ethnic minority populations is poor [2,3], which is sometimes used to justify claims that immigrants and ethnic minorities constitute a disproportionate burden on society [4]. As countries become ever more ethnically diverse, increasingly through the children born to immigrants as well as fresh migration, better surveillance and research is needed to provide reliable information [2,4]. Health systems and policymakers need to be aware of the likely implications of the demographic shift from a migration focus to an ethnicity focus. Linkage methods are likely to provide the backbone for this endeavour.

In conclusion, migrants and ethnic minorities in some high-income countries, including Scotland, have relatively lower mortality than the majority populations, and in Scotland this was most clearly so for non-white people born abroad. The explanations appear to lie, at least in part, in fewer risk factors. Future studies that explain these differences may point to ways of improving the health of the whole population. This will require widespread lifestyle and health surveys providing comparisons of the majority population with migrants and ethnic minorities [39] and qualitative research [2]. Variations in mortality by ethnic/migrant group status need to be considered in strategy and policy in relation to inequalities, inequities, and social determinants of health.

## Supporting information

S1 Checklist RECORD checklist. (DOCX)

S1 Protocol Data analysis plan. (DOCX)

S1 Appendix Supplementary methods and data file. (DOCX)

## Abbreviations

CHI: Community Health Index
CI: confidence interval
NHS: National Health Service
PY: person-years
RR: rate ratio
SES: socio-economic status
SHELS: Scottish Health and Ethnicity Linkage Study
SMR: standardised mortality ratio
UK/RoI: United Kingdom or Republic of Ireland

## References

[1] Rechel B, Mladovsky P, Deville W, Rijks B, Petrova-Benedict R, McKee M. Migration and health in the European Union. Maidenhead: Open University Press; 2011.

[2] Roura M. Unravelling migrants’ health paradoxes: a transdisciplinary research agenda. J Epidemiol Community Health. 2017;71(9):870–3. 10.1136/jech-2016-208439 28739838

[3] Parliamentary Office of Science and Technology. Ethnicity and health. London: Parliamentary Office of Science and Technology; 2007 p. 1–4. doi: 10.1080/13557850601002239

[4] Rechel B, Mladovsky P, Ingleby D, Mackenbach JP, McKee M. Migration and health in an increasingly diverse Europe. Lancet. 2013;381(9873):1235–45. doi: 10.1016/S0140-6736(12)62086-8 23541058 10.1016/S0140-6736(12)62086-8

[5] McKay L, Macintyre S, Ellaway A. Migration and health: a review of the international literature. Glasgow: University of Glasgow MRC Social and Public Sciences Unit; 2003.

[6] Wallace M, Kulu H. Low immigrant mortality in England and Wales: a data artefact? Soc Sci Med. 2014;120:100–9. doi: 10.1016/j.socscimed.2014.08.032 25233336 10.1016/j.socscimed.2014.08.032

[7] Marmot MG, Adelstein AM, Bulusu L. Immigrant mortality in England and Wales 1970–78: causes of death by country of birth. London: HMSO; 1984.

[8] Wild S, McKeigue P. Cross sectional analysis of mortality by country of birth in England and Wales, 1970–92. BMJ. 1997;314(7082):705–10. 9116545 10.1136/bmj.314.7082.705 PMC2126166

[9] Fischbacher C, Steiner M, Bhopal R, Chalmers J, Jamieson J, Knowles D, et al Variations in all cause and cardiovascular mortality by country of birth in Scotland, 1997–2003. Scott Med J. 2007;52(4):5–10. doi: 10.1258/rsmsmj.52.4.5 18092629 10.1258/rsmsmj.52.4.5

[10] McCartney G, Collins C, Walsh D, Batty GD. Why the Scots die younger: synthesizing the evidence. Public Health. 2012;126(6):459–70. doi: 10.1016/j.puhe.2012.03.007 22579324 10.1016/j.puhe.2012.03.007

[11] McCartney G, Bouttell J, Craig N, Craig P, Graham L, Lakha F, et al Explaining trends in alcohol-related harms in Scotland, 1991–2011 (I): the role of incomes, effects of socio-economic and political adversity and demographic change. Public Health. 2016;132:13–23. doi: 10.1016/j.puhe.2015.12.013 26917268 10.1016/j.puhe.2015.12.013

[12] Burns H. Health inequalities—why so little progress? Public Health. 2015;129(7):849–53. doi: 10.1016/j.puhe.2015.03.026 26027452 10.1016/j.puhe.2015.03.026

[13] Gruer L, Hart CL, Watt GC. After 50 years and 200 papers, what can the Midspan cohort studies tell us about our mortality? Public Health. 2017;142:186–95. doi: 10.1016/j.puhe.2015.06.017 26255248 10.1016/j.puhe.2015.06.017

[14] Bhopal RS. Migration, ethnicity, race and health in multicultural societies. 2nd edition Oxford: Oxford University Press; 2014.

[15] Barbieri M, Wilmoth JR, Shkolnikov VM, Glei D, Jasilionis D, Jdanov D, et al Data resource profile: the Human Mortality Database (HMD). Int J Epidemiol. 2015;44(5):1549–56. doi: 10.1093/ije/dyv105 26108436 10.1093/ije/dyv105 PMC4707194

[16] Rafnsson SB, Bhopal RS. Large-scale epidemiological data on cardiovascular diseases and diabetes in migrant and ethnic minority groups in Europe. Eur J Public Health. 2009;19(5):484–91. doi: 10.1093/eurpub/ckp073 19498046 10.1093/eurpub/ckp073

[17] Kulu GI, Stronks K. Defining ethnicity in health (care) research. Eur J Public Health. 2002;12(4):S71.

[18] Wallace M. Adult mortality among the descendants of immigrants in England and Wales: does a migrant mortality advantage persist beyond the first generation? J Ethn Migr Stud. 2016;42(9):1558–77.

[19] Bhopal R, Fischbacher C, Povey C, Chalmers J, Mueller G, Steiner M, et al Cohort profile: Scottish health and ethnicity linkage study of 4.65 million people exploring ethnic variations in disease in Scotland. Int J Epidemiol. 2011;40(5):1168–75. doi: 10.1093/ije/dyq118 20657021 10.1093/ije/dyq118

[20] Douglas A, Cezard G, Simpson CR, Steiner MF, Bhopal R, Bansal N, et al Pilot study linking primary care records to census, cardiovascular hospitalization and mortality data in Scotland: feasibility, utility and potential. J Public Health (Oxf). 2016;38(4):815–23. 28158483 10.1093/pubmed/fdv192

[21] Norredam M, Hansen OH, Petersen JH, Kunst AE, Kristiansen M, Krasnik A, et al Remigration of migrants with severe disease: myth or reality?—a register-based cohort study. Eur J Public Health. 2015;25(1):84–9. doi: 10.1093/eurpub/cku138 25201902 10.1093/eurpub/cku138

[22] Boyd KM. Ethnicity and the ethics of data linkage. BMC Public Health. 2007;7(1):318. 17996063 10.1186/1471-2458-7-318 PMC2151939

[23] Douglas A, Ward HJT, Bhopal R, Kirkpatrick T, Sayed-Rafiq A, Gruer L, et al Is the linkage of census and health data justified? Views from a public panel of the Scottish Health and Ethnicity Linkage study. J Public Health (Oxf). 2017 5 25 doi: 10.1093/pubmed/fdx060 28541459 10.1093/pubmed/fdx060

[24] Fischbacher CM, Cezard G, Bhopal RS, Pearce J, Bansal N. Measures of socioeconomic position are not consistently associated with ethnic differences in cardiovascular disease in Scotland: methods from the Scottish Health and Ethnicity Linkage Study (SHELS). Int J Epidemiol. 2014;43(1):129–39. doi: 10.1093/ije/dyt237 24355746 10.1093/ije/dyt237

[25] Bhopal R, Bansal N, Steiner M, Brewster DH. Does the ‘Scottish effect’ apply to all ethnic groups? All cancer, lung, colorectal, breast and prostate cancer in the Scottish Health and Ethnicity Linkage Cohort Study. BMJ Open. 2012;2:e001957 doi: 10.1136/bmjopen-2012-001957 23012329 10.1136/bmjopen-2012-001957 PMC3467629

[26] Sillitoe K. Ethnic origin: the search for a question. Popul Trends. 1978;13:25–30.

[27] Harding S, Rosato M, Teyhan A. Trends for coronary heart disease and stroke mortality among migrants in England and Wales, 1979–2003: slow declines notable for some groups. Heart. 2008;94(4):463–70. doi: 10.1136/hrt.2007.122044 17690159 10.1136/hrt.2007.122044 PMC2565582

[28] Bhopal RS, Humphry RW, Fischbacher CM. Changes in cardiovascular risk factors in relation to increasing ethnic inequalities in cardiovascular mortality: comparison of cross-sectional data in the Health Surveys for England 1999 and 2004. BMJ Open. 2013;3(9):e003485 doi: 10.1136/bmjopen-2013-003485 24052612 10.1136/bmjopen-2013-003485 PMC3780340

[29] Ruiz JM, Steffen P, Smith TB. Hispanic mortality paradox: a systematic review and meta-analysis of the longitudinal literature. Am J Public Health. 2013;103(3):e52–60. doi: 10.2105/AJPH.2012.301103 23327278 10.2105/AJPH.2012.301103 PMC3673509

[30] Abraido-Lanza AF, Dohrenwend BP, Ng-Mak DS, Turner JB. The Latino mortality paradox: a test of the “salmon bias” and healthy migrant hypotheses. Am J Public Health. 1999;89(10):1543–8. 10511837 10.2105/ajph.89.10.1543 PMC1508801

[31] Fenelon A. Revisiting the Hispanic mortality advantage in the United States: the role of smoking. Soc Sci Med. 2013;82:1–9. doi: 10.1016/j.socscimed.2012.12.028 23453311 10.1016/j.socscimed.2012.12.028 PMC3588600

[32] Razum O. Commentary: of salmon and time travellers-musing on the mystery of migrant mortality. Int J Epidemiol. 2006;35(4):919–21. doi: 10.1093/ije/dyl143 16847016 10.1093/ije/dyl143

[33] Gill PS, Kai J, Bhopal RS, Wild S. Health care needs assessment: black and minority ethnic groups In: Stevens A, Raftery J, Mant J, Simpson S, editors. Health care needs assessment: the epidemiologically based needs assessment reviews. Third Series. Abingdon: Radcliffe Medical Press; 2007 pp. 227–389.

[34] Wallace M, Kulu H. Migration and health in England and Scotland: a study of migrant selectivity and salmon bias. Popul Space Place. 2014;20(8):694–708.

[35] Balarajan R, Bulusu L. Mortality among immigrants in England and Wales, 1979–83 In: Britton M, editor. Mortality and geography: a review in the mid 1980’s. London: HMSO; 1990 pp. 103–21.

[36] Wild SH, Fischbacher C, Brock A, Griffiths C, Bhopal R. Mortality from all causes and circulatory disease by country of birth in England and Wales 2001–2003. J Public Health (Oxf). 2007;29(2):191–8. 17456532 10.1093/pubmed/fdm010

[37] Ikram UZ, Mackenbach JP, Harding S, Rey G, Bhopal RS, Regidor E, et al All-cause and cause-specific mortality of different migrant populations in Europe. Eur J Epidemiol. 2016;31(7):655–65. doi: 10.1007/s10654-015-0083-9 26362812 10.1007/s10654-015-0083-9 PMC4977342

[38] Scott AP, Timaeus IM. Mortality differentials 1991–2005 by self-reported ethnicity: findings from the ONS Longitudinal Study. J Epidemiol Community Health. 2013;67(9):743–50. doi: 10.1136/jech-2012-202265 23740930 10.1136/jech-2012-202265

[39] Becker E, Boreham R, Chaudhury M, Craig R, Deverill C, Doyle M, et al Health Survey for England 2004 The health of minority ethnic groups. London: Information Centre; 2006.

[40] Bansal N, Fischbacher CM, Bhopal RS, Brown H, Steiner MF, Capewell S, et al Myocardial infarction incidence and survival by ethnic group: Scottish Health and Ethnicity Linkage retrospective cohort study. BMJ Open. 2013;3(9):e003415 doi: 10.1136/bmjopen-2013-003415 24038009 10.1136/bmjopen-2013-003415 PMC3773657

[41] Gruer L, Cezard G, Clark E, Douglas A, Steiner M, Millard A, et al Life expectancy of different ethnic groups using death records linked to population census data for 4.62 million people in Scotland. J Epidemiol Community Health. 2016;70:1251–4. 10.1136/jech-2016-207426 PMC5136685 27473157

[42] Reus-Pons M, Vandenheede H, Janssen F, Kibele EUB. Differences in mortality between groups of older migrants and older non-migrants in Belgium, 2001–09. Eur J Public Health. 2016;26(6):992–1000. doi: 10.1093/eurpub/ckw076 27312258 10.1093/eurpub/ckw076

[43] Hunt S, Bhopal R. Self reports in research with non-English speakers. BMJ. 2003;327(7411):352–3. doi: 10.1136/bmj.327.7411.352 12919965 10.1136/bmj.327.7411.352 PMC1126776

