# Supplementary material for: Mortality, ethnicity, and country of birth on a national scale, 2001–2013: A retrospective cohort (Scottish Health and Ethnicity Linkage Study)
Source: PLoS Med. 2018 Mar 1;15(3):e1002515. doi: 10.1371/journal.pmed.1002515 (PMC5832197; doi:10.1371/journal.pmed.1002515)
Supplement: S1 Checklist — (DOCX) [file pmed.1002515.s001.docx]

**Supplementary File 1**

**The RECORD statement – checklist of items, extended from the STROBE statement, that should be reported in observational studies using routinely collected health data.**

**Please note that the line numbers and page numbers refer to the tracked changes version of the resubmitted manuscript**

|  | **Item No.** | **STROBE items** | **Location in manuscript where items are reported** | **RECORD items** | **Location in manuscript where items are reported** |
| --- | --- | --- | --- | --- | --- |
| **Title and abstract** | | | | | |
|  | 1 | (a) Indicate the study’s design with a commonly used term in the title or the abstract (b) Provide in the abstract an informative and balanced summary of what was done and what was found | Title and lines and methods and findings in abstract | RECORD 1.1: The type of data used should be specified in the title or abstract. When possible, the name of the databases used should be included.  RECORD 1.2: If applicable, the geographic region and timeframe within which the study took place should be reported in the title or abstract.  RECORD 1.3: If linkage between databases was conducted for the study, this should be clearly stated in the title or abstract. | Methods and findings section in abstract  Title and methods section in abstract  In title and abstract |
| **Introduction** | | | | | |
| Background rationale | 2 | Explain the scientific background and rationale for the investigation being reported | Introduction-paragraphs 4,5,6 |  |  |
| Objectives | 3 | State specific objectives, including any prespecified hypotheses | Introduction- paragraph 6 |  |  |
| **Methods** | | | | | |
| Study Design | 4 | Present key elements of study design early in the paper | Abstract-methods subsection; Methods; S1 Text |  |  |
| Setting | 5 | Describe the setting, locations, and relevant dates, including periods of recruitment, exposure, follow-up, and data collection | Methods-paragraph 2,3,4 |  |  |
| Participants | 6 | *(a) Cohort study* - Give the eligibility criteria, and the sources and methods of selection of participants. Describe methods of follow-up  *Case-control study* - Give the eligibility criteria, and the sources and methods of case ascertainment and control selection. Give the rationale for the choice of cases and controls  *Cross-sectional study* - Give the eligibility criteria, and the sources and methods of selection of participants  *(b) Cohort study* - For matched studies, give matching criteria and number of exposed and unexposed  *Case-control study* - For matched studies, give matching criteria and the number of controls per case | Scottish 1991 Census participants.  Follow up by data linkage  N/A  N/A  N/A | RECORD 6.1: The methods of study population selection (such as codes or algorithms used to identify subjects) should be listed in detail. If this is not possible, an explanation should be provided.  RECORD 6.2: Any validation studies of the codes or algorithms used to select the population should be referenced. If validation was conducted for this study and not published elsewhere, detailed methods and results should be provided.  RECORD 6.3: If the study involved linkage of databases, consider use of a flow diagram or other graphical display to demonstrate the data linkage process, including the number of individuals with linked data at each stage. | Methods-paragraphs 2,3,4; S1 Text, section 5; references to methods papers  No specific validation studies were done by the investigators  S1 Text; S1 Appendix- Fig A shows the linkage process and Table A provides the numbers of individuals linked by ethnic group |
| Variables | 7 | Clearly define all outcomes, exposures, predictors, potential confounders, and effect modifiers. Give diagnostic criteria, if applicable. |  | RECORD 7.1: A complete list of codes and algorithms used to classify exposures, outcomes, confounders, and effect modifiers should be provided. If these cannot be reported, an explanation should be provided. | These are all defined in paper, and S1 Text, especially in data analysis protocol-see S1 Protocol |
| Data sources/ measurement | 8 | For each variable of interest, give sources of data and details of methods of assessment (measurement).  Describe comparability of assessment methods if there is more than one group | All measures are standard ones. Comparability across ethnic groups was checked for socio-economic variables quantitatively using published methods (the results are in Table C of S1 Appendix). |  |  |
| Bias | 9 | Describe any efforts to address potential sources of bias | We checked the associations of SES variables (Table C of S1 Appendix) and examined trends using 3-year moving averages (results section sub- titled moving average analysis, and Table D of S1 Appendix) | | |
| Study size | 10 | Explain how the study size was arrived at | We aimed to study the entire Scottish population so study size was not calculated. We took the maximum period for which outcome data was deemed complete at outset of study. | | |
| Quantitative variables | 11 | Explain how quantitative variables were handled in the analyses. If applicable, describe which groupings were chosen, and why | The handling of variables is described in the methods section subtitled analysis, and in more detail in Table C of S1 Appendix and in our data analysis protocol (S1 Protocol). | | |
| Statistical methods | 12 | (a) Describe all statistical methods, including those used to control for confounding  (b) Describe any methods used to examine subgroups and interactions  (c) Explain how missing data were addressed  (d) *Cohort study* - If applicable, explain how loss to follow-up was addressed  *Case-control study* - If applicable, explain how matching of cases and controls was addressed  *Cross-sectional study* - If applicable, describe analytical methods taking account of sampling strategy  (e) Describe any sensitivity analyses | See Methods, subheading Analysis, and data analysis protocol (S1 Protocol)  See Methods, subheading Analysis  95.1% of the Census completing population was included, and there was no way to trace non-completers.  Loss to follow-up is a difficult matter in linkage studies. See Methods subheading Analysis for our approaches.  N/A  N/A | | |
| Data access and cleaning methods |  | .. |  | RECORD 12.1: Authors should describe the extent to which the investigators had access to the database population used to create the study population.  RECORD 12.2: Authors should provide information on the data cleaning methods used in the study. | We had access to Census data once it was linked. Other data were accessible prior to linkage in a safe haven at Information Services Division (ISD).  Data cleaning was done for the primary care data as published. [1] |
| Linkage |  |  |  | RECORD 12.3: State whether the study included person-level, institutional-level, or other data linkage across two or more databases. The methods of linkage and methods of linkage quality evaluation should be provided. | The linkage was between persons and involved several databases. The methods are described in detail, including in the data analysis plan (S1 Protocol) and have been published. |
| **Results** | | | | | |
| Participants | 13 | (a) Report the numbers of individuals at each stage of the study (*e.g.*, numbers potentially eligible, examined for eligibility, confirmed eligible, included in the study, completing follow-up, and analysed)  (b) Give reasons for non-participation at each stage.  (c) Consider use of a flow diagram | See Fig A and Table A of S1 Appendix | RECORD 13.1: Describe in detail the selection of the persons included in the study (*i.e.,* study population selection) including filtering based on data quality, data availability and linkage. The selection of included persons can be described in the text and/or by means of the study flow diagram. | See response to RECORD 6.3. There was no selection. We studied everyone linked. |
| Descriptive data | 14 | (a) Give characteristics of study participants (*e.g.*, demographic, clinical, social) and information on exposures and potential confounders  (b) Indicate the number of participants with missing data for each variable of interest  (c) *Cohort study* - summarise follow-up time (*e.g.*, average and total amount) | See Table B of S1 Appendix and text in the results with the subtitle Background information on the linked cohort.  Missing data in Census, mortality and primary care records may have been resolved by the Census or other authorities before releasing data e.g. the Census Office used methods to ensure 100% of the population had an ethnic group recorded. We accepted such adjustments. In the primary care data set, 91.2 percent of the records had smoking status recorded (104). We were not able to supplement these data. [1]  12-years except for data censoring | | |
| Outcome data | 15 | *Cohort study* - Report numbers of outcome events or summary measures over time  *Case-control study* - Report numbers in each exposure category, or summary measures of exposure  *Cross-sectional study* - Report numbers of outcome events or summary measures | Table 1 and 2  N/A  N/A |  |  |
| Main results | 16 | (a) Give unadjusted estimates and, if applicable, confounder-adjusted estimates and their precision (e.g., 95% confidence interval). Make clear which confounders were adjusted for and why they were included  (b) Report category boundaries when continuous variables were categorized  (c) If relevant, consider translating estimates of relative risk into absolute risk for a meaningful time period | Please note that completely unadjusted rates and ratios are not valuable given age differences but we give age adjusted rates and ratios (with 95% confidence intervals) by sex. In the methods we also explain how we use the variable born in the UK**/RoI or not. We also explain our use of 3 socio-economic variables.**  N/A  Although absolute risks are not the key output they are given in Tables 1 and 2 | | |
| Other analyses | 17 | Report other analyses done—e.g., analyses of subgroups and interactions, and sensitivity analyses | This is reported in the paper and in S1 Appendix, including our analysis in response to referees. | | |
| **Discussion** | | | | | |
| Key results | 18 | Summarise key results with reference to study objectives | Done – see results and S1 Appendix |  |  |
| Limitations | 19 | Discuss limitations of the study, taking into account sources of potential bias or imprecision. Discuss both direction and magnitude of any potential bias | See Discussion, paragraph 8 | RECORD 19.1: Discuss the implications of using data that were not created or collected to answer the specific research question(s). Include discussion of misclassification bias, unmeasured confounding, missing data, and changing eligibility over time, as they pertain to the study being reported. | These and other issues are discussed in this and published papers (Discussion, paragraph 8) |
| Interpretation | 20 | Give a cautious overall interpretation of results considering objectives, limitations, multiplicity of analyses, results from similar studies, and other relevant evidence | We have done this in the conclusion section of the abstract, the first paragraph of the discussion, paragraph 8 of the discussion, and in the final paragraph of the discussion | | |
| Generalisability | 21 | Discuss the generalisability (external validity) of the study results | The relative mortality of ethnic minorities is context specific but our methods and general observations have generalisable lessons given the state-of-the-art and these are considered in the discussion, in the context of the international literature with special reference to the UK. | | |
| **Other Information** | | | | | |
| Funding | 22 | Give the source of funding and the role of the funders for the present study and, if applicable, for the original study on which the present article is based | This information is on your website and will be transferred to the published paper |  |  |
| Accessibility of protocol, raw data, and programming code |  | .. |  | RECORD 22.1: Authors should provide information on how to access any supplemental information such as the study protocol, raw data, or programming code. | The data analysis protocol is provided as S1 Protocol. The raw data can be accessed in the safe haven in National Records Scotland on application (a contact is provided), and the programming code can be made available by the authors. |

*Reference: Benchimol EI, Smeeth L, Guttmann A, Harron K, Moher D, Petersen I, Sørensen HT, von Elm E, Langan SM, the RECORD Working Committee. The REporting of studies Conducted using Observational Routinely-collected health Data (RECORD) Statement. *PLoS Medicine* 2015; in press.

*Checklist is protected under Creative Commons Attribution ([CC BY](http://creativecommons.org/licenses/by/4.0/)) license.

1. Douglas A, Cezard G, Simpson CR, Steiner MF, Bhopal R, Bansal N, et al. Pilot study linking primary care records to Census, cardiovascular hospitalization and mortality data in Scotland: feasibility, utility and potential. J Public Health (Oxf). 2016;38(4):815-23.
