# Supplementary material for: Mortality, ethnicity, and country of birth on a national scale, 2001–2013: A retrospective cohort (Scottish Health and Ethnicity Linkage Study)
Source: PLoS Med. 2018 Mar 1;15(3):e1002515. doi: 10.1371/journal.pmed.1002515 (PMC5832197; doi:10.1371/journal.pmed.1002515)
Supplement: S1 Protocol — (DOCX) [file pmed.1002515.s002.docx]

# S1 Protocol

# Data analysis protocol for SHELS phase 4

[Data analysis protocol for SHELS phase 4 1](#_Toc454460994)

[1. Preparatory work (3-6 months) 3](#_Toc454460995)

[2. Data extraction (1-2 months) 4](#_Toc454460996)

[3. Data preparation (5-6 months) 6](#_Toc454460997)

[4. Statistical analysis (24 months) 8](#_Toc454460998)

[5. Disclosure review and release (2 weeks) 14](#_Toc454460999)

[Area: all hospitalisation, all mortality, infections, injuries, accident and poisoning 16](#_Toc454461000)

[Project analysis plan – Stage 1a: All-cause hospitalisation 18](#_Toc454461001)

[Project analysis plan – stage 1b: Readmission 19](#_Toc454461002)

[Project analysis plan – stage 2a: All-cause mortality 25](#_Toc454461003)

[Project analysis plan – stage 2b: Life expectancy 26](#_Toc454461004)

[Project analysis plan – stage 2c: Amenable and preventable mortality 27](#_Toc454461005)

[Project analysis plan – stage 3: Injuries, accidents and poisoning 30](#_Toc454461006)

[External causes of morbidity and mortality 31](#_Toc454461007)

[Project analysis plan – stage 4: Hospitalisations and deaths due to Infections 33](#_Toc454461008)

[Area: infections – Blood borne viruses (HIV, Hepatitis B and Hepatitis C) 41](#_Toc454461009)

[Project analysis plan – stage 5a: Prevalence and Incidence of diagnosis with Hepatitis C, HIV and Hepatitis B 43](#_Toc454461010)

[Project analysis plan – stage 5b: Incidence of late diagnosis with Hepatitis C, HIV and Hepatitis B 45](#_Toc454461011)

[Project analysis plan – stage 5c: Attendance at specialist services for Hepatitis C and HIV 47](#_Toc454461012)

[Project analysis plan – stage 6: Bowel Cancer Screening 49](#_Toc454461013)

[Project analysis plan – stage 7: Primary Care risk factors 52](#_Toc454461014)

| **Version** | **Date** | **Author/Editor** | **Comments** |
| --- | --- | --- | --- |
| **1.0** | **20 October 2014** | **Genevieve Cezard** | **Version 1.0 signed off by Data Analysis Group** |
| **2.0 draft** | **20 March 2015** | **Linda Williams** | Updates to all cause hospitalisations and bowel cancer screening. |
| 2.0 draft |  | GC and AD | Updates to section 3.3, 4.3 (SIMD as categorical), stage 2c (add preventable mortality), BBV analyses stage 5b, Bowel cancer screening analyses. Addition of tables for ICD10 codes for amenable mortality and avoidable hospitalisation and update to Infections codes table. |
| 2.0 draft | **05 May 2015** | LW | Modifications to all cause hospitalisation incl. readmissions, LOS and avoidable hospitalisations) |
| 2.0 draft | **01 Jul 2015** | AD | Minor changes to all cause hospitalisation and bowel cancer screening |
| 2.0 | **03 Sep 2015** | Anne Douglas | Version 2.0 signed off by data analysis subgroup and chairs of hospitalisations and bowel cancer screening subgroups. |
| 3.0 draft  3.1 | **21 January 2016**  **Feb-May2016**  **September 2016** | GC  LW,AD,GC  GC | Removal of “pair-wise comparisons”, keeping “comparison to the White Scottish” reference to ensure accurate statistical terminology.  Updates to bowel cancer screening analysis p50-52  Updates to SHELS analysis and Variables sections 4.2 and 4.3  Addition of Appendix E  Update to Injuries analysis p33  Add specific conditions for LOS analysis p19  Specified “chronic” for attendance at specialist services for Hepatitis C p47  Adding “Republic of Ireland (RoI)” when referring to adjustment for CoB (UK/RoI born) p10-11 and p17 |

## Preparatory work (3-6 months)

### 1.1 Data analysis plans

Subgroups will be convened for each health area. Subgroups will discuss and identify key and appropriate questions and hypotheses relevant to specific health areas and develop specific analysis plans detailing analysis objectives, questions and hypotheses, diagnoses and diagnostic codes (ICD), inclusion and exclusion criteria and methods. These analyses should be appropriate to the health area, take into account timelines and available resources as well as data limitations and ethical and methodological issues relating to the linked dataset. Analysis plans should prioritise key analyses using this agreed approach: 1. Prioritise hypothesis-based analyses and allow time for detailed exploration. 2. Look at other exploratory disease areas in a more simple analysis.

### 1.2 List of health and census variables for extraction

Final analysis plans should generate a list of required health and census variables. This list should be checked against health and census fields available. A list of agreed census variables used in phase 3 is detailed in the PID, and any additions to this list will need to be justified and discussed with NRS. Except for Blood borne viruses (BBV) data extracts which will be prepared at HPS, fields needed from the health extract should be communicated to the data extraction team at ISD taking into account the need for any additional secondary ‘generated’ fields (i.e., age at specific time, time to event for survival analyses).

## Data extraction (1-2 months)

**Location: ISD (eDRIS), NRS**

**Staff:** Markus Steiner, Genevieve Cezard, Anne Douglas, HPS team liaising with eDRIS

**Time estimation:** Health extracts are expected 1-2 month after approval received, around Spring 2014 the earliest.

### 2.1 Health data extract

The eDRIS data extraction team will provide an extract of data as agreed using the agreed list of variables and also with detailed documentation of the data extract. This should contain information on the file size, number of records/observations, time period of observations, a list of variables including codebook and any other relevant information about the datasets.

The data extract should contain all the identified fields, for all the cases (as identified by ICD codes from 2001 to the nearest complete date), for the required time period. We are asking for 10 years of hospitalisation records prior to the 2001 census for specific health outcome (infectious diseases). The extract will have all identifiable information and CHI removed but an encrypted CHI added instead. Once the data has been extracted, it will put on the SHELS shared folder at ISD and a meeting should be arranged between the data analyst and extraction team to double check the extract against the variable list and check for any missing information.

For phase 4, we would like the extract of all hospitalisation, all mortality, and both health areas infectious diseases and “injuries, accidents and poisoning” to be provided at the same time as format of the data needed is similar (SMR01 and death records). For all hospitalisation and the specific health areas data, in addition to relevant SMR01 data, related death records will be extracted. For the specific health area data, specific death records of the health area will also be extracted for people without a CHI or a SMR01 record. Bowel cancer screening data and Cancer registry data (SMR06) will be linked and provided separately. Health Protection Scotland (HPS) datasets will be provided after optimal linkage to the CHI and subsequent linkage to specific hospitalisation and death records.

### 2.2 Census data extract

NRS will provide an extract of agreed census data accessible in the safe setting at NRS only. This will be the same data used in phase 3 with the addition of the Standard Occupational Classification (SOC) and with a unique reference number for each new ISD extract. The reference number will be the same for the all hospitalisation and the all mortality data extracts which will be linked to the primary care sample dataset created during phase 3 available in the NRS safe setting and henceforth only one associated census extract will be required for the all hospitalisation and all mortality extracts. All census records should be provided with an indicator to identify those with a census link and those without. This will allow for subsequent exploration of linkage rates and assessment of bias. This extract should be checked for completeness prior to linkage with health data.

### 2.3 SHELS data extract

From phase 3, we will reuse 2 datasets which are held at NRS. The primary care sample dataset will be used for further linkage and analysis in order to enable the analysis of risk factors. To adjust the calculation of the census denominator (person year at risk) for the lost to follow-up, the death and migration file will be updated at ISD with the most recent data and continue to be routinely linked to the census file at NRS prior to linkage to the corresponding health dataset.

## Data preparation (5-6 months)

**Location:** ISD, HPS, NRS

**Staff:** Markus Steiner, Genevieve Cezard, Anne Douglas, HPS team

**Time estimation:** Data preparation should be done from May to October 2014 assuming data extracts are ready.

### 3.1 Data preparation at ISD – health data (3-4 months)

Preparation of 5 morbidity and mortality datasets will be carried out at ISD by the data analysis team (Markus Steiner and Genevieve Cezard, Anne Douglas for supervision and validation).

Before preparation of the dataset for data analysis the following steps are necessary:

a- Cross-check and overview of each datasets contents validation according to the requested information and provided codebook:

- Tabulation and documentation of the content of the dataset provided by the ISD data extraction team and interaction with the ISD team if issues arise.

- Check for outliers, inconsistent data or missing values

b- Corrections (arising from step a.):

- Apply appropriate corrections if needed

- Report in writing all the steps of the checking, decisions and corrections done by commenting on the code (SAS program)

c- Prepare datasets for the analysis:

- SAS programming to define the health outcomes of interest (e.g. 1^st^ events of specific diagnostic conditions within a specified time period (based on a known consistent period of data collection)) and creation of outcome variables as defined in the corresponding health area specific analysis plan.

- Clarifying the criteria for diagnostic exclusion (only specific diagnostic condition and/or exclusion of a whole diagnostic group) for the look back procedure. This will require input and clarification from sub-group chairs.

- Preparation and documentation of the datasets for outcomes of interest (e.g. 1^st^ event or any event), this includes complete variable and value labelling in SAS.

d- Validation of the created datasets

- Tabulation of the diagnostic events for each condition by year for the specified time period and double-check against publications (ISD, reference papers) and with subgroup chairs to determine if the numbers we get are sensible and expected.

- Validation of the created datasets done by a 2^nd^ person (Anne) using the commented upon code (in order to make sure that nothing has been missed)

e- Preparation of the health dataset(s) to be sent to NRS:

f- The created and validated health datasets are (newly) documented including file size, observation numbers, and variable list together with a codebook and MD5 hashes of the datasets and compressed/encrypted (WinZIP using AES encryption) for transfer to transfer medium. NHS secure FTP will be used if available at NRS.

g- Specification of the linkage output to be produced by NRS (to be done by analysts and emailed to NRS detailing what census records are required. As 1.3.2 above, we will need all census records)

### 3.2 Data preparation at HPS – health data (2-3 months)

### a-f- similar to 3.1

g- The prepared BBV data at HPS by a HPS analyst (Christian Schnier) are transferred to ISD (using SFTP) for CHI encryption before transfer from ISD to NRS.

### 3.3 Data checking at NRS – census and linked health data (1.5-2 months)

The team at NRS (currently David Campbell) will check the health and death data (extracted at ISD) received with the help of the attached file documentation. If any issue is noticed, the data analyst(s) concerned will be contacted before proceeding. Otherwise the health-census identifier will be linked to the health (or death) data.

NRS staff should make the full health dataset (without modifications in observation numbers or health outcome) linked with death and census information and the full census dataset linked with death and migration data* available in the safe setting at NRS. An identifier will be created for the purpose of linking the census/death dataset to the health dataset. An arbitrary flag variable will also be added in all datasets for linkage rate analysis (to allow the evaluation of the non-linkable proportion of events in the health or death data).

* NB. NRS does not collect data on deaths for Scottish residents who die outwith Scotland (ie England, Wales and abroad). Therefore these are not included in our deaths records obtained from ISD. NHSCR does include information it might receive on an ad-hoc basis about deaths abroad, and also ‘cleans’ the data at regular intervals and this will feed into ISD deaths data at that point.

Validation of the morbidity/mortality data, Bowel Cancer Screening/Cancer registry and BBV data linked to the census 2001 data will be carried out at NRS by data analysts (Markus Steiner, Genevieve Cezard, Anne Douglas for supervision).

a- Checking of the linked dataset contents:

- Look at numerators and denominators and check if they are as expected

- Check (frequency tables) all variables of the dataset

- Cross tabulate ethnicity with the outcome variable and breakdown by all the variables of interest to check distributions and the extent of missing values- Apply corrections if needed and comment on the code accordingly

- Main and all diagnosis as well as primary and all causes of deaths will be tabulated by ethnicity to allow decision within each subgroup when necessary

b- Prepare for disclosive issues:

- Define the different ethnic groups that are possible and relevant for the analyses

- Check for disclosive information for each ethnic breakdown selected, with the health outcomes of interest and the possible covariates

- Decide how ethnic groups will be categorised, with subgroup chairs, taking into account small numbers and which minority ethnic groups are of interest for each specific health outcome.

c- Report the linkage rates

- Calculate the relevant linkage rates (% of health/ISD records from 2001 onward linked to census information)

## Statistical analysis (24 months)

### 4.1 Development of statistical methods and procedures

**Location:** ISD & PHS/UoE/NRS

**Staff:** Markus, Duncan, Genevieve, Anne

**Time estimation:** end 2013 – mid 2015

Development of a statistical routine for calculating relative risks/risk ratios, for model checking and appropriate documentation:

1. Based on simulation datasets from phase 2 and 3 the methods around calculating risk ratios using Poisson regression were validated regarding the appropriate use of standard or robust variances

- Result: Use of **robust regression methods only** for all models; parameter estimates for ethnic groups and all covariates are included in the outputs.

1. The use of Negative binomial regression will be tested of each outcome to fit with the underlying over dispersed or under dispersed distribution of the data.
2. Review of diagnostic measures for both Poisson and Negative binomial regressions and necessary outputs.
3. Implementation of diagnostic measures is tested and a protocol for their appropriate use is developed. A decision had been made of what diagnostic measures are needed in the disclosure documents for the chairs to make a decision on which models are used (if not decided during the analysis stage within NRS by the data analysts)

- Result: The following diagnostic parameters are included in the outputs
  - Residuals * Predicted plot
  - Use of %vuong macro to compare nested models (<http://support.sas.com/kb/42/514.html>)
  - GoF statistics copied over from the conventional model as it isn’t in the output of the robust model output
  - Deviance/df should be close to 1
  - Output tables should contain:
    estimate / se / df / Wald / p + RR + n

1. The SAS macro code for risk ratio calculation will be cleaned and modified to include necessary diagnostic output, and generalized to be usable without modification for any health area. This should ensure a standard quality for all outputs using the specified method.
2. The possibility to use repeated events instead of 1^st^ events only will be evaluated and reviewed. If this proves valuable by the PI and chairs and if time is available, appropriate SAS code will be developed including a standard procedure for output.
3. The developed SAS code, as described above, is sent to NRS via email to be transferred onto the PCs in the safe setting by NRS staff.
4. All SAS programs should be documented in a separate table documenting: subject area (all hospitalisation, all mortality, accident and poisoning, infection, bowel cancer screening), file name, path, date of last modification, source file (if based on a previous version of a file) and comment (documentation of relevant changes; exemption is work in progress that has not been utilised to produce relevant output).

### 4.2 Statistical analysis of linked data

**Location:** NRS

**Staff:** Markus Steiner, Genevieve Cezard, Christian Schnier for assistance on HPS data, Anne Douglas for supervision

**Time estimation:** early 2014 – end 2015

Analyses (to adapt depending on each analysis plan):

1. Creating distribution tables (frequencies and percentages) for the health outcome of interest by ethnicity, age categories, sex, and any other relevant covariates as specified for each analysis.
2. Ethnic group categorisation will be discussed and agreed by the chairs and the PI before further analysis.
3. Calculating age adjusted rates for the outcome of interest by ethnic group and sex, with White Scottish as a reference using a person-year approach.
4. Calculating relative risks for events of interest. Poisson regression with robust error variances or Negative Binomial regression will be used depending on the underlying distribution of the data. Whenever possible, we will check the best model using goodness of fit measures. The outputs will contain parameter estimates for all covariates used in the model, and diagnostic measures as appropriate and developed in *3.1* (e.g. diagnostic plots). Latter might need clarification if diagnostic plots contain identifiable information.
5. Adjust for further risk factors as agree for each specific subgroup analysis.
6. Output tables from the regression models will be checked for disclosive figures and nominator/denominator figures checked against previously verified numbers from 3.2.1
7. Meetings with members of the subgroups in the safe setting in NRS will be necessary to decide on the final models prepared in 3.2.4.

### 4.3 Analysis of SHELS variables and expected results

1. **Ethnicity (main variable)**
   1. Self-reported ethnicity: is the main way we assess ethnicity. Other variables that relate to ethnicity e.g. country of birth or religion may be used to gain further insight into ethnic variations, or occasionally, used for stratified analysis.
   2. Anticipated results: experience shows that for most outcomes there are sizeable and potentially important ethnic variations when each minority group is compared against the Scottish White reference population.
   3. Interpretation: We will not have the required risk factor data for causal analysis but we will examine the effects of some co-variables as considered below (No 3 onwards).

Variables that are not directly related to ethnicity will be utilised as below.

1. **Sex**
   1. We analyse data by sex. We do this in the tradition of reporting vital statistics. In addition, we do this because there are often varying patterns of disease in males and females. While this implies that sex is an effect modifier, in SHELS our focus is on ethnic rather than sex variations. Formal analysis to compare patterns in males and females is not planned in SHELS4.
   2. Anticipated results: We expect some differences between men and women but we will simply signal these as of potential interest in future phases of SHELS.
   3. Interpretation: We will not attempt to interpret or explain sex variations.
2. **Age**
   1. Age greatly affects disease patterns in all populations. However, age is not of primary interest in SHELS, especially as numbers of outcomes decline rapidly when specific age groups are studied. However, we check age specific data in the safe haven. We do this as a data quality check and as a way of seeing whether there may be ethnic variations in the relationship between age group and disease outcome.
   2. Anticipated results: Mostly, we expect age specific results to be similar across ethnic groups. When this is so we will adjust for age in Poisson regression models. Where there are potentially important differences in age patterns between ethnic groups the subgroup needs to discuss these. The analysts need to draw this to the attention of subgroup chairs and the PI.
   3. Interpretation: Mostly, ethnic minority groups are younger than the reference. After age adjustment we assume age differences have been fully accounted for. This age adjusted analysis is our primary analysis answering the question: Are there ethnic variations in comparisons of each ethnic minority group with the White Scottish population?
3. **Country of birth (CoB)**
4. It is known that, mostly, there is a convergence of disease risk whereby immigrants’ risk becomes closer to that of the host (here reference) population. We do not have data on time since settlement. Country of birth is used in SHELS as an alternative. We would expect there to be differences in disease patterns in ethnic minority people born in the UK or Republic of Ireland (RoI) and those born abroad. Ideally, we would analyse these subgroups separately (as for sex) but the numbers of outcomes by ethnic group often becomes too small for meaningful analysis. So we will enter CoB into our model, when this is appropriate. CoB can also sometimes be used as a stratifying variable for example as used in the BBV analysis in SHELS 4.

Anticipated results: On the assumption of convergence, where the foreign born have a higher rate of disease than the UK/RoI born, we would expect the difference in the RR to decline after such adjustment. Where the foreign born have a lower rate the difference in the RR would rise.

1. Interpretation: We can interpret the adjusted data as indicating what the ethnic group patterns would be like if each ethnic group had the same country of birth pattern as the reference population i.e. White Scottish.
2. **Socio-economic variables**
   1. One major explanation for observed ethnic variation is difference in socio-economic status which is associated with both disease outcome and ethnicity. We will enter into our Poisson models one or more socio-economic variables based on our published SHELS 3 methodology as adapted by SHELS 4 data analysis subgroup.
   2. Anticipated results: Experience shows that in SHELS adjustment for these variables often makes little difference. Nonetheless, using our methodology, we would expect that where the ethnic minority group has higher (better) socio-economic status (SES) than the reference population, ethnic differences in RRs will become greater. Where the minority group has lower SES ethnic differences will become smaller.
   3. Interpretation: given our SES indicators are limited and may not be truly indicative of SES we should be cautious. However, mostly we will interpret findings as indicating the potential importance of SES in the pathways leading to ethnic differences in disease outcome.
   4. Are SES indicators confounding factors?

A confounding factor ought not to be on the causal pathways. In the field of ethnicity, race and health it is agreed that SES is likely to be on the causal pathway e.g. through racial discrimination leading to relative poverty and psychosocial stress, subsequently leading to disease. SHELS subgroups should be sure that for their outcomes SES is not on the causal pathway before labelling SES indicators as confounding factors. This requires work on causal pathways (including directed acyclic graphs).

- 1. The method to assess various socio-economic measures as potential confounders in the health and ethnicity association has been published:

Fischbacher CM, Cezard G, Bhopal RS, Pearce J, Bansal N. Measures of socioeconomic position are not consistently associated with ethnic differences in cardiovascular disease in Scotland: methods from the Scottish Health and Ethnicity Linkage Study (SHELS). Int J Epidemiol 2014 Feb;43(1):129-39.

In SHELS4, we intend to use a comparatively simpler analysis in 2 steps.

First, we will analyse the strengh of association between each socio-economic measures of interest and the health outcome under study by ethnic group. We will select for inclusion in the regression analysis the measures which are similarly associated with the outcome across ethnic groups as potentially valid confounders.

In phase 4 and for the census participants of any age, we will use:

- Scottish Index for Multiple Deprivation (SIMD) (100% complete and use as a categorical variable, either quintiles or deciles etc)) ^1^
- Household tenure (98.5% complete)
- Education at the household level (95% complete)

We will also investigate the use of a combined individual and household level education where the individual level of education is used for people aged 16-74 and the household level for children and elderly.

Where analysis of adults and particularly 16-74 year olds is appropriate we will use:

- Education at the individual level (100% complete in this age group)
- Economic activity last week (100% complete in this age group)

When there is a wide number of outcomes under study for a specific health area, the analysis to choose socio-economic variables for inclusion will be restricted to groups of diseases rather than each one of the whole set.

1. **Other variables: acknowledge causal factors**
2. From the primary care data set we have some variables that are agreed as causal e.g. smoking and diabetes for our outcomes of CVD and all-cause mortality.
3. Anticipated results: we expect that when entered into the model these covariables will alter the RR. The direction of change is predictable e.g. if the minority group has a higher prevalence of the RF the differences in RRs compared to the reference will decrease. When the prevalence is lower the difference in RR will increase.
4. Interpretation: We will interpret changes as indicative of causal effects of the variables. Our measures are imperfect so these causal effects are likely to be underestimates as occurs with misclassification (and regression dilution) errors.

**The nature of variables**

It is not always clear how a variable such as CoB or SIMD or education is fitting into a causal pathway. Such variables could be thought of as confounders, effect modifiers or causal risk factors. This requires detailed consideration and this process can be done through causal graphs. Causal graphs have now been prepared and are being considered.

^1^ _DAP V1.0 24032015 did not state that SIMD should be used as a categorical variable – SIMD was used as a continuous variable in adjusted models for all cause mortality and BBV. Checks on all cause mortality results showed no difference in RRs and CIs when SIMD used as categorical variable_

## Disclosure review and release (2 weeks)

### 5.1 Preparing outputs for disclosure review and release

Outputs should be prepared for release guided by the data analysis plan and subgroup discussions and follow an agreed table template (see Appendix A). Outputs for release are categorised as intermediary and final outputs, Intermediary outputs are only for distribution within the research groups to inform a decision process and not for distribution or use in publications. Intermediary outputs provide important background information and should take into account future final table requests particularly in relation to changes in group categorisations. Where possible, detailed intermediary outputs should be viewed in the safe setting with subgroup chairs.

Outputs should be double checked for errors prior to submission and cells checked against NRS disclosure guidelines (Appendix B).

All frequency numbers below 5 included will be removed and those above 6 included will be rounded to the nearest 5. Percentages should be derived from rounded numbers.

All outputs should be accompanied with a detailed contents list providing the following information:

- Linked file: i.e. all hospitalisation
- Date of analysis (manually including to avoid automatic update)
- Number of tables/figures requested
- Number of request (i.e., phase 3/ all hospitalisation/output 1/version 1)
- Intended use of output (i.e., internal use, presentation, publication)

Each table/figure in the output should be clearly and appropriately labelled and numbered. When submitting revised or amended outputs, replacing or relating to previously released outputs, this should be clearly stated with changes made i.e., this output is a revision of table 3 in previously released output 1, ethnic categories have changed.

One table will be updated and provided with every output for disclosure containing the following information:

- Path, filename and title of the document
- Date of preparation for disclosure
- Date reviewed in NRS
- Result of review
- Filename and Title/Version of document superseded. This is only important for documents which are replaced by a newer version for whatever reason (data error, etc). It is in the responsibility of every project member to securely destroy superseded documents (see Appendix C).

When outputs are ready for submission they should be placed in an appropriately labelled and dated folder which should be placed in the ‘Outputs for review’ folder on the D drive. A copy of the output should also be placed in the health area folder for that analysis and added onto the ‘list of outputs submitted for review’ so that we have an updated record of all outputs submitted.

An email is then sent to the project IT support team at NRS (currently Robert Collins) informing them and the disclosure team of outputs submitted. Where possible the IT team and NRS disclosure committee should be informed in advance of number and timing of outputs for removal and review, respectively.

### 5.2 Transfer of outputs from safe setting PC to NRS committee

The NRS IT team should confirm transfer of outputs to the disclosure team by sending data analysts a confirmatory email.

### 5.3 Release of documents from NRS disclosure committee to data analysts

NRS disclosure committee should advise data analysts of timing of review and release on receipt of outputs. Any queries should be raised with the lead data analyst including the whole data analysis team in all emails. On release, the committee should make clear any changes made or failed outputs. Outputs should be emailed to all project data analysts.

### 5.4 Sharing and use of released outputs

Outputs should be shared with the subgroup chair and subgroup members as relevant. Any further sharing should be limited to the steering group and on a need to know basis. Sharing of outputs outwith the steering and subgroup is prohibited and would require approval by the project steering group and NRS disclosure committee. Outputs should only be used for the intended use as detailed on submission. Further use needs to come back through disclosure committee for review and approval. A secure option to store and access documents online rather than using printouts will be re-evaluated to prevent the use of outdated output documents and minimise the need for printouts.

### 5.5 Storage and destruction of released outputs

All printed outputs should be marked and dated. File and paper outputs should be treated as confidential and stored securely. Paper copies of printouts should be kept to a minimum. File and paper outputs should be destroyed and disposed of securely when superseded or replaced by subsequent updated outputs (Appendix C).

### 5.6 Publication and presentation to conferences

All outputs used in publication and presentation need to be cleared by the disclosure committee. All materials about to be shared externally to SHELS members should be sent to currently David Campbell (and members of the NRS disclosure team): abstracts and manuscripts before submission and slides before presentation. Publications and presentation can be sent to CSO for information.

# Area: all hospitalisation, all mortality, infections, injuries, accident and poisoning

### (SMR01 & death registrations)

### Objectives

To explore:

1. Ethnic inequalities in all-cause hospitalisation, readmission and length of stay
2. Ethnic inequalities in all-cause mortality
3. Ethnic inequalities in hospitalisation and/or mortality in the following conditions:

- Injuries, accidents and poisoning
- Infections

Analysis will be done by ethnicity (the reference group being White Scottish), by sex and examining the potential effect of available covariates (see next page)

Additional

1. Explore ethnic inequalities in unmet need for health care by looking at differences in avoidable hospitalisation, unplanned readmission and amenable mortality

### Outcomes

For the study period (1/5/2001 - 30/4/2013):

1. 1st hospital discharge/hospital death for any disease
2. Subsequent hospital discharge
3. Length of stay for any hospital admission
4. Death for any cause
5. 1^st^ hospital discharge/ any death for specific diseases (accidents, injuries and poisoning and infections)

### Note

Results for “all other ethnic groups” will be available at NRS but not included in publications because of difficulties of interpretation in such a mixed group.

### Main covariates (sex stratified)

- Age

Socioeconomic variables which will be added in the regression analysis if assessed as having a consistent and positive association with the outcome across ethnic group:

For population of any age:

- Area based socioeconomic status: Scottish Index of Multiple Deprivation (SIMD)
- House ownership
- Highest qualification (household)

We will also investigate the use of a combined individual and household level education where the individual level of education is used for people aged 16-74 and the household level for children and elderly.

For adult population (restricted to 16-74 years old):

- Highest qualification (individual)
- Economic activity in the previous week of census completion

Other covariates:

- Country of birth (UK/ROI/Non-UK/ROI)
- Religion
- Marital status
- Urban/rural indicator
- Health board
- Occupational risk group

### For injuries, accidents and poisoning and infections specifically:

### Diagnosis criteria:

All six diagnostic positions in the SMR01 dataset or all 11 positions in the death records will be included to identify specific diseases.

### Diagnostic groups (ICD 10):

- For accidents and poisoning Table 1
- For infections Table 2

### Deaths:

The numbers of deaths outside hospital is usually small. Assuming that such deaths count for less than 20% of incident cases, hospitalisations, including deaths in hospital, and deaths outside hospital will be combined for analysis.

### Minimum number of cases:

A specific disease will be analysed by ethnic group if the total number of incident events for this disease reached 1000 cases per year. This should allow enough numbers for minority ethnic groups to be studied as well as provide sufficient statistical power for the analysis.

## Project analysis plan – Stage 1a: All-cause hospitalisation

**(1) Incidence of any hospital admission, by ethnicity and sex**

| **Background** | Little is known about differences in all-cause hospitalisation rates by ethnic group in Scotland or in the rest of the UK. |
| --- | --- |
| **Aim** | Establish the pattern of ethnic differences in the incidence of all-cause hospitalisation in Scotland |
| **Hypotheses** | 1. There are ethnic variations (≥10%) in rates of all-cause hospitalisation in men and women. We hypothesise these will vary in pair-wise comparisons between specific ethnic groups and White Scottish.  2. These variations cannot be fully explained by available covariates |
| **Data and ICD codes** | Linkage census database, SMR01. |
| **Numerator** | Admission between May 2001 –April 2013  The total number of hospitalisations over the period of interest |
| **Denominator** | Linked 2001 census population – no age restriction  Person Years adjusted for deaths and leaving NHS Scotland |
| **Tabulation** | For both sexes and each ethnic group |
| **Analysis** | For both sexes and each ethnic group  Incidence (number of first events), report absolute numbers  Inspect age stratified results (10 years age band, children, adult) – not for disclosure  Report differences in age-adjusted hospitalisation rates and age-adjusted risk ratios (Poisson) with confidence intervals and p-values  Adjust for other relevant covariates |
| **Adjust/stratify** | Explore the effect of a range of available covariates |

## Project analysis plan – stage 1b: Readmission

**(1) Readmission following first hospital discharge, by ethnicity and sex**

| **Background** | Little is known about differences in readmission rates by ethnic group in Scotland or the rest of the UK. |
| --- | --- |
| **Aim** | Explore ethnic variations in readmission rates |
| **Hypotheses** | 1. There are ethnic variations (≥10%) in readmission rates in men and women. We hypothesise these will vary between specific ethnic groups and White Scottish.  2. These variations cannot be fully explained by available covariates |
| **Data and ICD codes** | Linkage census database, SMR01.  1- All-cause hospitalisation, all ICD codes |
| **Numerator** | Admission to hospital within 30 days (<30 days) of previous hospital discharge  b) Total number of readmissions due to any urgent or emergency code (or non-elective) |
| **Denominator** | Linked 2001 census population, with first hospital admission between May 2001 – April 2013  (Total number of admissions (excluding hospitalisations of patients who died within 30 days of discharge – including death in hospital - and hospitalisations with insufficient post-discharge time to close of study) |
| **Tabulation** | By 10 year age band, sex, ethnic group and 3-4 years period (for trend analysis) |
| **Analysis** | Inspect 30 days urgent/emergency/non-elective readmission by sex and ethnic group, adjusted for age.  Report differences in age-adjusted discharge rates and age-adjusted risk ratios (based on Poisson regression) with confidence intervals and p-values  Adjust for other relevant covariates  Examine in-hospital mortality rates  Trend analysis: same analysis for each 3-4 years period with look-back |
| **Adjust/stratify** | Explore the effect of a range of available covariates |
| **Future research** | Adjustment for comorbidity and initiating disease. Adjust for LOS. |

***Project analysis plan – stage 1c: Length of stay (LOS)***

| **Background** | Little is known about differences in LOS by ethnic group in Scotland or in the rest of the UK. |
| --- | --- |
| **Aim** | Explore ethnic variations in length of stay |
| **Hypotheses** | There are ethnic variations (≥10%) in length of stay in men and women. We hypothesise LOS will be longer in minority ethnic groups compared to White Scottish.  These variations cannot be fully explained by adjustment for available covariates |
| **Data and ICD codes** | Linkage census database, SMR01.  All-cause hospitalisation (excluding individuals with no hospital stay)  LOS of 0 (day cases) will be adjusted to 0.5 days |
| **Numerator** | Length of stay of any hospitalisation |
| **Denominator** | Linked 2001 census population, with hospital admission between May 2001 – April 2013  No denominator, but may need to assume independence of visit lengths if it is not possible to include repeated measures on a patient basis. |
| **Tabulation** | By sex and ethnic group |
| **Analysis** | Between May 2001 – April 2013:  LOS for all hospitalisations and 3 selected specific conditions (with high number of hospitalisations)  Adjust for age, and other relevant covariates. Examine the distribution ‘tail’ (>90 days) and examine distribution of diagnosis codes/chapters. Perform sensitivity analysis of LOS including/excluding excessively long stays. Check fit of data to Poisson, log and Negative Binomial distributions.  LOS will be compared over periods of years to allow a trend analysis if numbers and time allow. |
| **Adjust/stratify** | Explore the effect of a range of available covariates. Possible further stratified/adjusted analyses (eg by diagnostic group or indicator condition) will be considered in due course. |
| **Future research** | Inclusion of DRG/HRG codes (or ICD10 chapters). Calculate expected LOS from case mix in order to calculate excess LOS, would need Charlson Index and/or secondary hospitalisation causes. |

***Project analysis plan – stage 1d: Avoidable hospitalisation***

**(1) Avoidable hospitalisation, by ethnicity and sex**

| **Background** | Nothing is known about differences in avoidable hospitalisation by ethnic group in Scotland and little in the rest of the UK. Most of the work in this area has been done in the US |
| --- | --- |
| **Aim** | Calculate incidence rate by ethnic group |
| **Hypotheses** | There may be ethnic variations in the proportion of hospitalisations that can be considered avoidable. We hypothesise there will be variations between specific ethnic groups and White Scottish.  These variations cannot be fully explained by adjustment for available covariates |
| **Data and ICD codes** | Linkage census database, hospital discharge  ICD codes as per the Department of Health’s Outcomes Framework |
| **Numerator** | Hospital admissions (for those aged 19 years and over) identified as avoidable (using NHS outcome framework definition) between May 2001 – April 2013 (or latest reliable date)  - all avoidable hospitalisations  - acute  - chronic  Total number of events (all, chronic, acute) |
| **Denominator** | Linked 2001 census population aged 19 years and above  Person Years adjusted for deaths and leaving NHS Scotland |
| **Tabulation** | By sex and ethnic group |
| **Analysis** | For both sexes and each ethnic group  Number of avoidable hospitalisations (all, acute, chronic)  Inspect stratified results by age– not for disclosure  Report differences in age-adjusted avoidable hospitalisations rates and age-adjusted risk ratios (using Poisson regression) with confidence intervals and p-values  Further explore the adjustment for socio-economic covariates if time and resources allow. |
| **Adjust/stratify** | Explore the effect of a range of covariates |

**Table 1: Hospitalisation codes considered to be avoidable (NHS Outcomes Framework)**

| **ICD10 codes** | **Condition** | **Acute** | **Chronic** |
| --- | --- | --- | --- |
| A02.0 | Salmonella enteritis | X |  |
| A04 | Other bacterial intestinal infections | X |  |
| A05.9 | Bacterial foodborne intoxication, unspecified | X |  |
| A07.2 | Cryptosporidiosis | X |  |
| A08 | Viral and other specified intenstinal infections | X |  |
| A09 | Diarrhoea and gastroenteritis of presumed infectious origin | X |  |
| A36 | Diphtheria | X |  |
| A37 | Whooping cough | X |  |
| A69.0 | Necrotizing ulcerative stomatitis | X |  |
| B05 | Measles | X |  |
| B06 | Rubella | X |  |
| B16.1 | Acute hepatitis B with delta-agent without hepatic coma | X |  |
| B16.9 | Acute hepatitis B without delta-agent and without hepatic coma | X |  |
| B18.0 | Chronic viral hepatitis B with delta-agent |  | X |
| B18.1 | Chronic viral hepatitis B without delta-agent |  | X |
| B26 | Mumps | X |  |
| D50.1 | Sideropenic dysphagia |  | X |
| D50.8 | Other iron deficiency anemias |  | X |
| D50.9 | Iron deficiency anemia, unspecified |  | X |
| D51 | Vitamin B12 deficiency anaemia |  | X |
| D52 | Folate deficiency anaemia |  | X |
| E10 | Type 1 diabetes mellitus |  | X |
| E11 | Type 2 diabetes mellitus |  | X |
| E12 | Malnutrition-related diabetes mellitus |  | X |
|  |  |  |  |
| E13 | Other specified diabetes mellitus |  | X |
| E14 | Unspecified diabetes mellitus |  | X |
| E86 | Volume depletion | X |  |
| F00 | Dementia in alzheimers |  | X |
| F01 | Vascular dementia |  | X |
| F02 | Dementia in other diseases |  | X |
| F03 | Unspecified dementia |  | X |
| G25.3 | Myoclonus | X |  |
| G40 | Epilepsy and recurrent seizures |  | X |
| G41 | Status epilepticus |  | X |
| H66 | Suppurative and unspecified otitis media | X |  |
| H67 | Otitis media in diseases classified elsewhere | X |  |
| I10X | Essential (primary) hypertension |  | X |
| I11.0 | Hypertensive heart disease with heart failure |  | X |
| I11.9 | Hypertensive heart disease without heart failure |  | X |
| I13.0 | Hypertensive heart and renal disease with (congestive) heart failure |  | X |
| I20 | Angina pectoris |  | X |
| I24.0 | Coronary thrombosis not resulting in myocardial infarction | X |  |
| I24.8 | Other forms of acute ischaemic heart disease | X |  |
| I24.9 | Acute ischaemic heart disease, unspecified | X |  |
| I25 | Chronic ischaemic heart disease |  | X |
| I48X | Atrial fibrillation and flutter |  | X |
| I50 | Heart failure |  | X |
| I89.1 | Lymphangitis | X |  |
| J02 | Acute pharyngitis | X |  |
| J03 | Acute tonsillitis | X |  |
| J04.0 | Acute laryngitis | X |  |
| J06 | Acute upper respiratory infections multiple and unsp sites | X |  |
| J10 | Influenza due to other identified influenza virus | X |  |
| J11 | Influenza due to unidentified influenza virus | X |  |
| J13X | Pneumonia due to Streptococcus pneumoniae | X |  |
| J14 | Pneumonia due to Hemophilus influenzae | X |  |
| J15.3 | Pneumonia due to streptococcus, group B | X |  |
| J15.4 | Pneumonia due to other streptococci | X |  |
| J15.7 | Pneumonia due to Mycoplasma pneumoniae | X |  |
| J15.9 | Unspecified bacterial pneumonia | X |  |
| J16.8 | Pneumonia due to other specified infectious organisms | X |  |
| J18.1 | Lobar pneumonia, unspecified organism | X |  |
| J18.8 | Other pneumonia, unspecified organism | X |  |
| J20 | Acute bronchitis |  | X |
| J31.2 | Chronic pharyngitis | X |  |
| J41 | Simple and mucopurulent chronic bronchitis |  | X |
| J42X | Unspecified chronic bronchitis |  | X |
| J43 | Emphysema |  | X |
| J44 | Other chronic obstructive pulmonary disease |  | X |
| J45 | Asthma |  | X |
| J46X | Status asthmaticus |  | X |
| J47X | Bronchiectasis |  | X |
| J81X | Pulmonary edema |  | X |
| K02 | Dental caries | X |  |
| K03 | Other diseases of hard tissues of teeth | X |  |
| K04 | Diseases of pulp and periapical tissues | X |  |
| K05 | Gingivitis and periodontal diseases | X |  |
| K06 | Other disorders of gingiva and edentulous alveolar ridge | X |  |
| K08 | Other disorders of teeth and supporting structures | X |  |
| K09.8 | Other cysts of oral region, not elsewhere classified | X |  |
| K09.9 | Cyst of oral region, unspecified | X |  |
| K12 | Stomatitis and related lesions | X |  |
| K13 | Other diseases of lip and oral mucosa | X |  |
| K20 | Esophagitis | X |  |
| K21 | Gastro-oesophageal reflux disease | X |  |
| K25.0 | Acute gastric ulcer with hemorrhage | X |  |
| K25.1 | Acute gastric ulcer with perforation | X |  |
| K25.2 | Acute gastric ulcer with both hemorrhage and perforation | X |  |
| K25.4 | Chronic or unspecified gastric ulcer with hemorrhage | X |  |
| K25.5 | Chronic or unspecified gastric ulcer with perforation | X |  |
| K25.6 | Chronic or unspecified gastric ulcer with both hemorrhage and perforation | X |  |
| K26.0 | Acute duodenal ulcer with hemorrhage | X |  |
| K26.1 | Acute duodenal ulcer with perforation | X |  |
| K26.2 | Acute duodenal ulcer with both hemorrhage and perforation | X |  |
| K26.4 | Chronic or unspecified duodenal ulcer with hemorrhage | X |  |
| K26.5 | Chronic or unspecified duodenal ulcer with perforation | X |  |
| K26.6 | Chronic or unspecified duodenal ulcer with both hemorrhage and perforation | X |  |
| K27.0 | Acute peptic ulcer, site unspecified, with hemorrhage | X |  |
| K27.1 | Acute peptic ulcer, site unspecified, with perforation | X |  |
| K27.2 | Acute peptic ulcer, site unspecified, with both hemorrhage and perforation | X |  |
| K27.4 | Chronic or unspecified peptic ulcer, site unspecified, with hemorrhage | X |  |
| K27.5 | Chronic or unspecified peptic ulcer, site unspecified, with perforation | X |  |
| K27.6 | Chronic or unspecified peptic ulcer, site unspecified, with both hemorrhage and perforation | X |  |
| K28.0 | Acute gastrojejunal ulcer with hemorrhage | X |  |
| K28.1 | Acute gastrojejunal ulcer with perforation | X |  |
| K28.2 | Acute gastrojejunal ulcer with both hemorrhage and perforation | X |  |
| K28.4 | Chronic or unspecified gastrojejunal ulcer with hemorrhage | X |  |
| K28.5 | Chronic or unspecified gastrojejunal ulcer with perforation | X |  |
| K28.6 | Chronic or unspecified gastrojejunal ulcer with both hemorrhage and perforation | X |  |
| K52 | Other noninfective gastroenteritis and colitis | X |  |
| L01 | Impetigo | X |  |
| L02 | Cutaneous abscess, furuncle and carbuncle | X |  |
| L03 | Cellulitis | X |  |
| L04 | Acute lymphadenitis | X |  |
| L08.0 | Pyoderma | X |  |
| L08.8 | Other specified local infections of skin and subcutaneous tissue | X |  |
| L08.9 | Local infection of skin and subcutaneous tissue, unspecified | X |  |
| L88 | Pyoderma gangrenosum | X |  |
| L98.0 | Pyogenic granuloma | X |  |
| M01.4 | Rubella arthritis | X |  |
| N10 | Acute tubulo-interstitial nephritis | X |  |
| N11 | Chronic tubulo-interstitial nephritis | X |  |
| N12 | Tubulo-interstitial nephritis not specified as acute or chronic | X |  |
| N13.6 | Pyonephrosis | X |  |
| N15.9 | Renal tubulo-interstitial disease, unspecified | X |  |
| N30.0 | Acute cystitis | X |  |
| N30.8 | Other cystitis | X |  |
| N30.9 | Cystitis, unspecified | X |  |
| N39.0 | Urinary tract infection, site not specified | X |  |
| O15 | Eclampsia | X |  |
| R56 | Convulsions, not elsewhere classified | X |  |

## Project analysis plan – stage 2a: All-cause mortality

**(1) Mortality, by ethnicity and sex**

| **Background** | There are known all-cause mortality differences by country of birth in Scotland. Fischbacher et al showed that, compared to those born in Scotland, men born in Ireland had higher all-cause mortality, but lower mortality was observed in people born in Northern Ireland (women only), Pakistan, Bangladesh (men only), China and Hong Kong (men only). |
| --- | --- |
| **Aim** | Compare and refine the findings for all-cause mortality in Scotland by ethnic group with those of Fischbacher et al. where country of birth was used as a proxy for ethnicity. |
| **Hypotheses** | 1. Large variations (≥10%) in all-cause mortality in males and females are expected as previously shown.  2. These variations will be partly attenuated by adjusting for country of birth and socio-economic covariates for White ethnic groups |
| **Data and ICD codes** | Linkage census database, death records  All ICD codes |
| **Numerator** | (a) Death (all causes) between May 2001 – April 2013 (or latest reliable date) |
| **Denominator** | Linked 2001 census population  Person Years adjusted for deaths and leaving NHS Scotland |
| **Tabulation** | By sex and ethnic group |
| **Analysis** | For both sexes and each ethnic group  Number of deaths  Inspect stratified results for age at death (10 years age band, children, younger adults, older adults) – not for disclosure  A preliminary analysis will report age-adjusted mortality rates and age-adjusted risk ratios (Poisson) with confidence intervals and p-values by country of birth for comparison purpose with Fischbacher’s findings.  Report differences in age-adjusted mortality rates and age-adjusted risk ratios (Poisson) with confidence intervals and p-values  Adjust for other relevant variables  Results will be explored for specific age groups and for main causes of death if numbers, time and resources allow.  Trend analysis (salmon bias): same analysis for each 3-4 years period |
| **Adjust/stratify** | Can be refined using country of birth analysis.  Explore the effect of a range of available covariates |

## Project analysis plan – stage 2b: Life expectancy

**(1) Mortality, by ethnicity and sex**

| **Background** | Life expectancy by ethnic group has never been calculated in Scotland |
| --- | --- |
| **Aim** | Calculate two estimates of life expectancy by ethnic group, one around the Census 2001 (with SHELS) and the other around the Census 2011 (with ethnicity recorded on the death record). |
| **Hypotheses** | There are ethnic variations in life expectancy in Scotland. |
| **Data and ICD codes** | (a) Linkage census database and death records (3 years: 2001-2003)  (b) Census 2011 and death records (3 years: 2012-2014) |
| **Numerator** | (a) Deaths in 2001 – 2003  (b) Deaths in 2012 – 2014 |
| **Denominator** | (a) Linked 2001 census population  (b) 2011 census population |
| **Tabulation** | By sex and ethnic group |
| **Analysis** | For both sexes and each ethnic group  Life expectancy can be calculated for small area/group under certain conditions of numbers for the area/group:   - More than 5000 people - More than 40 deaths   It will be calculated by sex or combined depending on numbers.  Mortality rates will be calculated for 5 or 10 years age bands.  Life expectancy calculation may only be possible for the larger ethnic group and for older age groups. |
| **Adjust/stratify** | Explore the effect of a range of available covariates |

## Project analysis plan – stage 2c: Amenable and preventable mortality

**(1) Amenable mortality, by ethnicity and sex**

| **Background** | Nothing is known about amenable and preventable mortality differences by ethnic group in Scotland or the rest of the UK. Work in this field has been mainly conducted in the US, NZ and Singapore. |
| --- | --- |
| **Aim** | Using pre-established definitions of amenable and preventable mortality, calculate amenable mortality rates by ethnic group. |
| **Hypotheses** | There may be ethnic variations in the proportion of deaths that could be considered amenable to healthcare interventions. We hypothesise these will vary between specific ethnic groups and White Scottish.  These variations cannot be fully explained by adjustment for available covariates |
| **Data and ICD codes** | Linkage census database, death records  ICD codes as defined by the Office for National Statistics |
| **Numerator** | (a) Deaths identified as amenable (using ONS definition) between May 2001 – April 2013 (or latest reliable date) |
| **Denominator** | Linked 2001 census population  Person Years adjusted for deaths and leaving NHS Scotland |
| **Tabulation** | By sex and ethnic group |
| **Analysis** | For both sexes and each ethnic group  Number of amenable and preventable deaths  Inspect stratified results by age– not for disclosure  Report differences in age-adjusted mortality rates and age-adjusted risk ratios (Poisson) with confidence intervals and p-values  Further explore the adjustment for socio-economic covariates if time and resources allow. |
| **Adjust/stratify** | Explore the effect of a range of available covariates |

**Table 2: Causes of death (classified using the International Classification of Diseases, tenth revision) considered to be avoidable**

Based on underlying cause of death

| **Condition group and cause** | **ICD-10 codes** | **Age** | **Amenable** | **Preventable** |
| --- | --- | --- | --- | --- |
|  |  |  |  |  |
| **Infections** |  |  |  |  |
| Tuberculosis | A15-A19, B90 | 0-74 | • | • |
| Selected invasive bacterial and protozoal infections | A38-A41, A46, A48.1, B50-B54, G00, G03, J02, L03 | 0-74 | • |  |
| Hepatitis C | B17.1, B18.2 | 0-74 | • | • |
| HIV/AIDS | B20-B24 | All | • | • |
| **Neoplasms** |  |  |  |  |
| Malignant neoplasm of lip, oral cavity and pharynx | C00-C14 | 0-74 |  | • |
| Malignant neoplasm of oesophagus | C15 | 0-74 |  | • |
| Malignant neoplasm of stomach | C16 | 0-74 |  | • |
| Malignant neoplasm of colon and rectum | C18-C21 | 0-74 | • | • |
| Malignant neoplasm of liver | C22 | 0-74 |  | • |
| Malignant neoplasm of trachea, bronchus and lung | C33-C34 | 0-74 |  | • |
| Malignant melanoma of skin | C43 | 0-74 | • | • |
| Mesothelioma | C45 | 0-74 |  | • |
| Malignant neoplasm of breast | C50 | 0-74 | • | • |
| Malignant neoplasm of cervix uteri | C53 | 0-74 | • | • |
| Malignant neoplasm of bladder | C67 | 0-74 | • |  |
| Malignant neoplasm of thyroid gland | C73 | 0-74 | • |  |
| Hodgkin's disease | C81 | 0-74 | • |  |
| Leukaemia | C91, C92.0 | 0-44 | • |  |
| Benign neoplasms | D10-D36 | 0-74 | • |  |
| **Nutritional, endocrine and metabolic** |  |  |  |  |
| Diabetes mellitus | E10-E14 | 0-49 | • | • |
| **Drug use disorders** |  |  |  |  |
| Alcohol related diseases, excluding external causes | F10, G31.2, G62.1, I42.6, K29.2, K70, K73, K74 (excl. K74.3-K74.5), K86.0 | 0-74 |  | • |
| Illicit drug use disorders | F11-F16, F18-F19 | 0-74 |  | • |
| **Neurological disorders** |  |  |  |  |
| Epilepsy and status epilepticus | G40-G41 | 0-74 | • |  |
| **Cardiovascular diseases** |  |  |  |  |
| Rheumatic and other valvular heart disease | I01-I09 | 0-74 | • |  |
| Hypertensive diseases | I10-I15 | 0-74 | • |  |
| Ischaemic heart disease | I20-I25 | 0-74 | • | • |
| DVT with pulmonary embolism | I26, I80.1-I80.3, I80.9, I82.9 | 0-74 |  | • |
| Cerebrovascular diseases | I60-I69 | 0-74 | • |  |
| Aortic aneurysm and dissection | I71 | 0-74 |  | • |
| **Respiratory diseases** |  |  |  |  |
| Influenza (including swine flu) | J09-J11 | 0-74 | • | • |
| Pneumonia | J12-J18 | 0-74 | • |  |
| Chronic obstructive pulmonary disorder | J40-J44 | 0-74 |  | • |
| Asthma | J45-J46 | 0-74 | • |  |
| **Digestive disorders** |  |  |  |  |
| Gastric and duodenal ulcer | K25-K28 | 0-74 | • |  |
| Acute abdomen, appendicitis, intestinal obstruction, cholecystitis/lithiasis, pancreatitis, hernia | K35-K38, K40-K46, K80-K83, K85, K86.1-K86.9, K91.5 | 0-74 | • |  |
| **Genitourinary disorders** |  |  |  |  |
| Nephritis and nephrosis | N00-N07, N17-N19, N25-N27 | 0-74 | • |  |
| Obstructive uropathy and prostatic hyperplasia | N13, N20-N21, N35, N40, N99.1 | 0-74 | • |  |
| **Maternal and infant** |  |  |  |  |
| Complications of perinatal period | P00-P96, A33 | All | • |  |
| Congenital malformations, deformations and chromosomal anomalies | Q00-Q99 | 0-74 | • |  |
| **Unintentional injuries** |  |  |  |  |
| Transport Accidents | V01-V99 | All |  | • |
| Accidental Injury | W00-X59 | All |  | • |
| **Intentional injuries** |  |  |  |  |
| Suicide and self inflicted injuries | X60-X84, Y10-Y34 | All |  | • |
| Homicide/Assault | X85-Y09, U50.9 | All |  | • |
| Misadventures to patients during surgical and medical care | Y60-Y69, Y83-Y84 | All | • | • |

## Project analysis plan – stage 3: Injuries, accidents and poisoning

**(1) Incidence of Injuries, accidents and poisoning hospitalisation and death, by ethnicity and sex**

| **Background** | Nothing is known about accidents and poisoning by ethnic group in Scotland. In the Netherland, Stirbu found all ethnic minorities combined had an increased mortality of all injuries together, pedestrian accidents, drowning and poisoning compared to native Dutch but lower mortality in accidents involving cyclists and motorcyclists. Ethnic inequalities were higher among children and younger adults. |
| --- | --- |
| **Aim** | Establish the pattern of ethnic differences in the incidence of the above conditions in Scotland |
| **Hypotheses** | 1. There are ethnic variations (≥10%) in rates of the above outcomes in male and female. We hypothesise these will vary between specific ethnic groups and White Scottish.  2. Patterns of ethnic variations in children, younger and older adults differ.  3. These variations cannot be explained by available covariates |
| **Data and ICD codes** | Linkage census database, SMR01 and death records  ICD codes for all discharges (ICD10) : Chapter XIX and XX  See table 1 for breakdown |
| **Numerators** | (a) First and recurrent events : Hospital discharge or death between May 2001 – April 2013 (or latest reliable date)  (b) for any of the above ICD codes in any of the 6 discharge diagnostic positions in the SMR01 dataset or main cause of death for the above conditions. * |
| **Denominator** | Linked 2001 census population  Person Years adjusted for deaths and migrations |
| **Tabulation** | For each diagnosis: By sex and ethnic group |
| **Analysis** | For each diagnosis or for groups of diagnoses:  Incidence (number of first and recurrent events), report absolute numbers  Inspect age stratified results (children aged 0-14, in 5 years age band; younger adults aged 15 to 24, adults aged 25 to 64 and older adults aged 65 +, in 10 years age band) – not for disclosure  Report differences in age-adjusted discharge rates and age-adjusted risk ratios (Poisson) with confidence intervals and p-values  Adjust for other relevant variables  Explore differences in children, younger and older adults if numbers allow. Numbers are expected to be too small among the youngest (0-4) and the elderly (65+) from the non-white minority ethnic groups. |
| **Adjust/stratify** | Explore the effect of a range of available covariates including occupational risk and area-level deprivation? |

**Table 3: ID diagnostic groups and associated ICD codes for injuries, accident and poisoning**

| **Chapter** | **ID Group** | **ICD-10 Codes** |
| --- | --- | --- |
| XX | External causes of morbidity and mortality | **V01-Y98** |
|  | **Accidents** | **V01-X59** |
|  | Transport accidents | V01-V99 |
|  | Pedestrian and cyclist injured in transport accident | V01-V19 |
|  | Motorcycle rider and car occupant injured in transport accident | V21-V49 |
|  | Other external causes of accidental injuries | W00-X59 |
|  | Falls | W00-W19 |
|  | Exposure to inanimate mechanical forces  (including cut/pierced, Truck by, crushing, machinery) | W20-W49 |
|  | Exposure to animate mechanical forces | W50-W64 |
|  | **Accidental poisoning** | X40-X49 |
|  | Accidental exposure to other and unspecified factors | X58-X59 |
|  | Assault | X85-Y09 |
|  |  |  |
| XIX | **Injury, poisoning and certain other consequences of external causes** | **S00-T98** |
|  | **Injury** | **S00-S99** |
|  | Injury of the head | S00-S09 |
|  | Injury of the neck | S10-S19 |
|  | Injury of the thorax | S20-S29 |
|  | Injury of the abdomen, lower back, lumbar spine and pelvis | S30-S39 |
|  | Injury of the shoulder and upper arm | S40-S49 |
|  | Injury of the elbow and forearm | S50-S59 |
|  | Injury of the wrist and hand | S60-S69 |
|  | Injury of the hip and thigh | S70-S79 |
|  | Injury of the knee and lower leg | S80-S89 |
|  | Injury of the ankle and foot | S90-S99 |
|  | Burns and corrosions | T20-T32 |
|  | **Poisoning and toxic effect** | **T36-T65** |
|  | Poisoning by drug, medicaments and biological substances | T36-T50 |

*The subgroup agreed to:

Exclude all records where there is a cause of death for any intentional harm (ICD10 codes X60-X84).

Exclude records where the underlying cause of death is an ‘Undetermined intent’, ICD10 codes Y10-Y34.

Include records for ICD10 codes for complications of medical or surgical care (Y40-Y84) and subgroup will review analyses and agree any decisions about including these – or not – for release.

Exclude records for hospital admissions with admission type =31 (patient injury self-inflicted)

## Project analysis plan – stage 4: Hospitalisations and deaths due to Infections

**(1) Incidence of hospitalisation and death due to infections, by ethnicity and sex**

| **Background** | Little is known about differences by ethnic group in Scotland in deaths and hospitalisation rates due to infections. A recent paper from New Zealand by Baker et al (2012) showed marked variations by ethnic group in the incidence of hospitalisation for infections. |
| --- | --- |
| **Aim** | Establish the pattern of ethnic differences in the incidence of infections hospitalisation and death in Scotland |
| **Hypotheses** | 1. There are ethnic variations (≥10%) in rates of the above outcomes. We hypothesise these will vary between specific ethnic groups and White Scottish.  2. These variations cannot be explained by available covariates |
| **Data and ICD codes** | Linkage census database, SMR01 and death records  ICD codes for all discharge (ICD10) - See table 2 for breakdown |
| **Numerators** | (a) 1.First event: hospital discharge or death between May 2001 – April 2013 (or latest reliable date) and 2. All events: hospital discharge or death between May 2001 – April 2013 (excluding second admissions within 30 days of 1^st^ admission)  (b) for any of the 6 discharge diagnosis or any cause of death for the above conditions  (c) 5 years look-back prior to 2001 will be considered for specific infections    For maternal and perinatal infections, the period of interest will depend on the denominator available. |
| **Denominator** | Linked 2001 census population – no age restriction  Person Years adjusted for deaths and migrations  Maternal and perinatal infections will need separate denominators (number of births/mother) |
| **Tabulation** | For each diagnosis: By sex and ethnic group |
| **Analysis** | For each diagnosis or for groups of diagnoses :  Incidence (number of first events), report absolute numbers  Inspect age stratified results– not for disclosure  Report differences in Age-adjusted discharge rates and Age-adjusted risk ratios (Poisson) with confidence intervals and p-values  Adjust for other relevant variables  Analysis will be done for each specific disease on a case by case basis, in general when more than 1000 annual cases are available but if the prevalence is high in minority ethnic group as for Tuberculosis, fewer annual cases may be required. |
| **Adjust/stratify** | Explore the effect of a range of available covariates |

**Table 3: ID diagnostic groups and associated ICD codes for infections**

| **ID Group** | **ICD10** | **code title** |
| --- | --- | --- |
| **Enteric infections 1** | A00 | Cholera |
|  | A01 | Typhoid and paratyphoid fevers |
|  | A02 | Other salmonella infections |
|  | A03 | Shigellosis |
|  | A04 | Other bacterial intestinal infections |
|  | A05 | Other bacterial foodborne intoxications |
|  | A06 | Amoebiasis |
|  | A07 | Other protozoal intestinal diseases |
|  | A08 | Viral and other specified intestinal infections |
| **Enteric symptoms 2** | A09X | Diarrhoea and gastroenteritis of presumed infectious origin |
|  | I880 | Nonspecific mesenteric lymphadenitis |
|  | R11X | Nausea and vomiting |
|  | *A09* | *Diarrhoea and gastroenteritis of presumed infectious origin*  *(only for death between 2011 and 2013 due to change in ICD10 version used)* |
| **Septicaemia 3** | A40 | Streptococcal septicaemia |
|  | A41 | Other septicaemia |
| **STI 4** | A50 | Congenital syphilis |
|  | A51 | Early syphilis |
|  | A52 | Late syphilis |
|  | A53 | Other and unspecified syphilis |
|  | A54 | Gonococcal infection |
|  | A55X | Chlamydial lymphogranuloma (venereum) |
|  | A56 | Other sexually transmitted chlamydial diseases |
|  | A57X | Chancroid |
|  | A58X | Granuloma inguinale |
|  | A59 | Trichomoniasis |
|  | A60 | Anogenital herpesviral [herpes simplex] infection |
|  | A63 | Other predominantly sexually transmitted diseases NEC |
|  | A64X | Unspecified sexually transmitted disease |
|  | N290 A | Late syphilis of kidney |
| **HIV/AIDS 5** | B20 | Human immunodef virus dis result infectious parasitic dis |
|  | B21 | Human immunodef virus dis resulting malignant neopl |
|  | B22 | Human immunodef virus dis resulting in other spec dis |
|  | B23 | Human immunodef virus dis resulting in other conditions |
|  | B24X | Unspecified human immunodefiency virus [HIV] disease |
| **Meningococcal 6** | A39 | Meningococcal infection |
| **CNS viral infections 7** | A801 | Acute paralytic poliomyelitis wild virus imported |
|  | A802 | Acute paralytic poliomyelitis wild virus indigenous |
|  | A803 | Acute paralytic poliomyelitis other and unspecified |
|  | A804 | Acute nonparalytic poliomyelitis |
|  | A809 | Acute poliomyelitis unspecified |
|  | A810 | Creutzfeldt-Jakob disease |
|  | A811 | Subacute sclerosing panencephalitis |
|  | A812 | Progressive multifocal leukoencephalopathy |
|  | A818 | Other slow virus infections of central nervous system |
|  | A819 | Slow virus infection of central nervous system unspecified |
|  | A82 | Rabies |
|  | A83 | Mosquito-borne viral encephalitis |
|  | A84 | Tick-borne viral encephalitis |
|  | A85 | Other viral encephalitis not elsewhere classified |
|  | A86X | Unspecified viral encephalitis |
|  | A87 | Viral meningitis |
|  | A88 | Other viral infections of central nervous system NEC |
|  | A89X | Unspecified viral infection of central nervous system |
| **CNS general infections 8** | G00 | Bacterial meningitis not elsewhere classified |
|  | G01X A | Meningitis in bacterial diseases classified elsewhere |
|  | G02.- A | Meningitis in other infectious and parasitic diseases EC |
|  | G030 | Nonpyogenic meningitis |
|  | G039 | Meningitis unspecified |
|  | G04 | Encephalitis myelitis and encephalomyelitis |
|  | G05.- A | Encephalitis myelitis and encephalomyelitis in diseases CE |
|  | G06 | Intracranial and intraspinal abscess and granuloma |
|  | G07X A | A Intracranial and intraspinal abscess and granuloma dis EC |
|  | G08X | Intracranial and intraspinal phlebitis and thrombophlebitis |
|  | G09X | Sequelae of inflammatory diseases of central nervous system |
|  | G610 | Guillain-Barre syndrome |
| **Eye infections 9** | B30 | Viral conjunctivitis |
|  | H000 | Hordeolum and other deep inflammation of eyelid |
|  | H03 A | Disorders of eyelid in diseases classified elsewhere |
|  | H043 | Acute and unspecified inflammation of lacrimal passages |
|  | H050 | Acute inflammation of orbit |
|  | H100 | Mucopurulent conjunctivitis |
|  | H102 | Other acute conjunctivitis |
|  | H103 | Acute conjunctivitis unspecified |
|  | H109 | Conjunctivitis unspecified |
|  | H130 A | Filarial infection of conjunctiva |
|  | H131 A | Conjunctivitis in infectious and parasitic diseases EC |
|  | H160 | Corneal ulcer |
|  | H190 A | Scleritis and episcleritis in diseases classified elsewhere |
|  | H191 A | Herpesviral keratitis and keratoconjunctivitis |
|  | H192 A | Keratitis and keratoconjunctivitis oth infec/parasit dis EC |
|  | H220 A | Iridocyclitis in infectious and parasitic diseases EC |
|  | H440 | Purulent endophthalmitis |
|  | H451 A | Endophthalmitis in diseases classified elsewhere |
| **Ear infections 10** | H600 | Abscess of external ear |
|  | H601 | Cellulitis of external ear |
|  | H602 | Malignant otitis externa |
|  | H603 | Other infective otitis externa |
|  | H609 | Otitis externa unspecified |
|  | H62 A | Disorders of external ear in diseases classified elsewhere |
|  | H65 | Nonsuppurative otitis media |
|  | H66 | Suppurative and unspecified otitis media |
|  | H67.- A | Otitis media in diseases classified elsewhere |
|  | H680 | Eustachian salpingitis |
|  | H70 | Mastoiditis and related conditions |
|  | H730 | Acute myringitis |
|  | H750 A | A Mastoiditis in infectious and parasitic diseases EC |
|  | H830 | Labyrinthitis |
|  | H940 A | Acoustic neuritis in infectious and parasitic diseases EC |
| **Upper RTI 11** | J00X | Acute nasopharyngitis [common cold] |
|  | J01 | Acute sinusitis |
|  | J02 | Acute pharyngitis |
|  | J03 | Acute tonsillitis |
|  | J04 | Acute laryngitis and tracheitis |
|  | J05 | Acute obstructive laryngitis [croup] and epiglottitis |
|  | J06 | Acute upper respiratory infections multiple and unsp sites |
|  | J32 | Chronic sinusitis |
|  | J340 | Abscess furuncle and carbuncle of nose |
|  | J36X | Peritonsillar abscess |
|  | J37 | Chronic laryngitis and laryngotracheitis |
|  | J390 | Retropharyngeal and parapharyngeal abscess |
|  | J391 | Other abscess of pharynx |
| **Tuberculosis 12** | A15 | Resp TB bacteriologically and histologically confirmed |
|  | A16 | Respiratory TB not confirmed bact or histologically |
|  | A17.-D | Tuberculosis of nervous system |
|  | A18 | Tuberculosis of other organs |
|  | A19 | Miliary tuberculosis |
|  | N740 A | Tuberculous infection of cervix uteri |
|  | N741 A | Female tuberculous pelvic inflammatory disease |
|  | J65X | Pneumoconiosis associated with tuberculosis |
| **Acute LRTI 13** | A481 | Legionnaires' disease |
|  | A482 | Nonpneumonic Legionnaires' disease [Pontiac fever] |
|  | B59X | Pneumocystosis |
|  | **J09X** | **Influenza due to certain identified influenza virus** |
|  | J10 | Influenza due to other identified influenza virus |
|  | J11 | Influenza virus not identified |
|  | J12 | Viral pneumonia not elsewhere classified |
|  | J13X | Pneumonia due to Streptococcus pneumoniae |
|  | J14X | Pneumonia due to Haemophilus influenzae |
|  | J15 | Bacterial pneumonia not elsewhere classified |
|  | J16 | Pneumonia due to other infectious organisms NEC |
|  | J17 A | Pneumonia in diseases classified elsewhere |
|  | J18 | Pneumonia organism unspecified |
|  | J20 | Acute bronchitis |
|  | J21 | Acute bronchiolitis |
|  | J22X | Unspecified acute lower respiratory infection |
|  | *J09* | *Influenza due to certain identified influenza virus*  *(only for death between 2011 and 2013 due to change in ICD10 version used)* |
|  | *J44* | *Other chronic obstructive pulmonary disease*  *(only for death between 2011 and 2013 due to change in ICD10 version used)* |
| **Chronic LRTI 14** | J40X | Bronchitis not specified as acute or chronic |
|  | J41 | Simple and mucopurulent chronic bronchitis |
|  | J42X | Unspecified chronic bronchitis |
|  | J440 | Chronic obstruct pulmonary dis with acute lower resp infec |
|  | J47X | Bronchiectasis |
|  | J85 | Abscess of lung and mediastinum |
|  | J86 | Pyothorax |
|  | J988 | Other specified respiratory disorders |
| **Heart & Circulatory infections 15** | B332 | Viral carditis |
|  | I00X | Rheumatic fever without mention of heart involvement |
|  | I01 | Rheumatic fever with heart involvement |
|  | I02 | Rheumatic chorea |
|  | I05 | Rheumatic mitral valve diseases |
|  | I06 | Rheumatic aortic valve diseases |
|  | I07 | Rheumatic tricuspid valve diseases |
|  | I08 | Multiple valve diseases |
|  | I09 | Other rheumatic heart diseases |
|  | I301 | Infective pericarditis |
|  | I33 | Acute and subacute endocarditis |
|  | I38X | Endocarditis valve unspecified |
|  | I39.-A | Endocarditis and heart valve disorders in diseases EC |
|  | I400 | Infective myocarditis |
|  | I410 A | A Myocarditis in bacterial diseases classified elsewhere |
|  | I411 A | A Myocarditis in viral diseases classified elsewhere |
|  | I412 A | A Myocarditis in other infectious and parasitic diseases EC |
|  | I430 A | A Cardiomyopathy in infectious & parasitic diseases CE |
|  | I790 A | Aneurysm of aorta in diseases classified elsewhere |
|  | I791 A | Aortitis in diseases classified elsewhere |
| **Oral infections 16** | K02 | Dental caries |
|  | K044 | Acute apical periodontitis of pulpal origin |
|  | K046 | Periapical abscess with sinus |
|  | K050 | Acute gingivitis |
|  | K052 | Acute periodontitis |
|  | K053 | Chronic periodontitis |
|  | K113 | Abscess of salivary gland |
|  | K122 | Cellulitis and abscess of mouth |
| **GI tract infections 17** | K230 A | Tuberculous oesophagitis |
|  | K231 A | Megaoesophagus in Chagas' disease |
|  | K25 | Gastric ulcer |
|  | K26 | Duodenal ulcer |
|  | K27 | Peptic ulcer site unspecified |
|  | K28 | Gastrojejunal ulcer |
|  | K293 | Chronic superficial gastritis |
|  | K294 | Chronic atrophic gastritis |
|  | K295 | Chronic gastritis unspecified |
|  | K35 | Acute appendicitis |
|  | K36X | Other appendicitis |
|  | K37X | Unspecified appendicitis |
|  | K61 | Abscess of anal and rectal regions |
|  | K630 | Abscess of intestine |
|  | K632 | Fistula of intestine |
|  | K650 | Acute peritonitis |
|  | K678 A | Other disorders of peritoneum in infectious diseases EC |
|  | K908 | Other intestinal malabsorption |
|  | K930 A | TB disord intestine peritoneum and mesenteric glands |
| **Hepatic infections 18** | K750 | Abscess of liver |
|  | K770 A | Liver disorders in infectious and parasitic diseases EC |
|  | K830 | Cholangitis |
| **Viral hepatitis 19** | B15 | Acute hepatitis A |
|  | B16 | Acute hepatitis B |
|  | B17 | Other acute viral hepatitis |
|  | B18 | Chronic viral hepatitis |
|  | B19 | Unspecified viral hepatitis |
| **Kidney infections 20** | N00 | Acute nephritic syndrome |
|  | N05 | Unspecified nephritic syndrome |
|  | N10X | Acute tubulo-interstitial nephritis |
|  | N136 | Pyonephrosis |
|  | N151 | Renal and perinephric abscess |
| **Urinary tract infections 21** | N300 | Acute cystitis |
|  | N341 | Nonspecific urethritis |
|  | N351 | Postinfective urethral stricture not elsewhere classified |
|  | N37 A | Urethral disorders in diseases classified elsewhere |
|  | N390 | Urinary tract infection site not specified |
| **Reproductive tract infections Male 22** | N410 | Acute prostatitis |
|  | N411 | Chronic prostatitis |
|  | N412 | Abscess of prostate |
|  | N413 | Prostatocystitis |
|  | N431 | Infected hydrocele |
|  | N45 | Orchitis and epididymitis |
|  | N481 | Balanoposthitis |
|  | N482 | Other inflammatory disorders of penis |
|  | N490 | Inflammatory disorders of seminal vesicle |
|  | N49 | Inflammatory disorders of male genital organs NEC |
|  | N51 A | Disorders of male genital organs in diseases EC |
| **Reproductive tract infections Female 23** | N70 | Salpingitis and oophoritis |
|  | N71 | Inflammatory disease of uterus except cervix |
|  | N72X | Inflammatory disease of cervix uteri |
|  | N73 | Other female pelvic inflammatory diseases |
|  | N74 A | Female pelvic inflammatory disorders in diseases EC |
|  | N751 | Abscess of Bartholin's gland |
|  | N764 | Abscess of vulva |
|  | N87 | Dysplasia of cervix uteri |
| **Skin infections typical 24** | A46X | Erysipelas |
|  | L00X | Staphylococcal scalded skin syndrome |
|  | L01 | Impetigo |
|  | L02 | Cutaneous abscess furuncle and carbuncle |
|  | L03 | Cellulitis |
|  | L04 | Acute lymphadenitis |
|  | L050 | Pilonidal cyst with abscess |
|  | L08 | Other local infections of skin and subcutaneous tissue |
| **Breast infections 25** | N61X | Inflammatory disorders of breast |
| **Osteomyelitis 26** | M462 | Osteomyelitis of vertebra |
|  | M463 | Infection of intervertebral disc (pyogenic) |
|  | M464 | Discitis unspecified |
|  | M465 | Other infective spondylopathies |
| **Joint infections 27** | M00 | Pyogenic arthritis |
|  | M01 A | Direct infections joint in infectious and parasitic dis EC |
| **Connective tissue infections 28** | M021 | Postdysenteric arthropathy |
|  | M023 | Reiter's disease |
|  | M03 A | Postinfective and reactive arthropathies in diseases EC |
|  | M600 | Infective myositis |
|  | M630 A | Myositis in bacterial diseases classified elsewhere |
|  | M631 A | Myositis in protozoal and parasitic infections EC |
|  | M632 A | Myositis in other infectious diseases classified elsewhere |
|  | M650 | Abscess of tendon sheath |
|  | M651 | Other infective (teno)synovitis |
|  | M680 A | Synovitis and tenosynovitis in bacterial diseases EC |
|  | M710 | Abscess of bursa |
|  | M711 | Other infective bursitis |
|  | M896 | Osteopathy after poliomyelitis |
| **Neoplasms from infection 29** | C11 | Malignant neoplasm of nasopharynx |
|  | C161 | Malignant neoplasm of fundus of stomach |
|  | C162 | Malignant neoplasm of body of stomach |
|  | C163 | Malignant neoplasm of pyloric antrum |
|  | C164 | Malignant neoplasm of pylorus |
|  | C165 | Malignant neoplasm of lesser curvature of stomach unsp |
|  | C166 | Malignant neoplasm of greater curvature of stomach unsp |
|  | C168 | Malignant neoplasm overlapping lesion of stomach |
|  | C169 | Malignant neoplasm of stomach unspecified |
|  | C210 | Malignant neoplasm of anus unspecified |
|  | C211 | Malignant neoplasm of anal canal |
|  | C220 | Malignant neoplasm liver cell carcinoma |
|  | C46 | Kaposi's sarcoma |
|  | C53 | Malignant neoplasm of cervix uteri |
|  | D002 | Carcinoma in situ stomach |
|  | D013 | Carcinoma in situ anus and anal canal |
|  | D06 | Carcinoma in situ of cervix uteri |
| **Postoperative infections 30** | T802 | Infections following infusion transfusion & therap inject |
|  | T814 | Infection following a procedure not elsewhere classified |
|  | T826 | Infect and inflammatory reaction due to cardiac valve pros |
|  | T827 | Infect inflamm reac due oth card vasc devs implant and graft |
|  | T835 | Infect inflam react due pros dev implt & graft urinary syst |
|  | T836 | Infect inflam react due pros dev implant graft in gen tract |
|  | T845 | Infect and inflammatory reaction due to internal joint pros |
|  | T846 | Infect and inflamm react due int fixation dev [any site] |
|  | T847 | Inf inflam reac due oth int orth prosth devs implts & grfts |
|  | T857 | Inf inflamm react due oth int prosth devs implants & grafts |
|  | T874 | Infection of amputation stump |
| **Adverse effect of ID treatment 31** | R761 | Abnormal reaction to tuberculin test |
|  | R762 | False-positive serological test for syphilis |
|  | T36 | Poisoning by systemic antibiotics |
|  | T37 | Poisoning by oth systemic anti-infective and antiparasitics |
|  | T485 | Poisoning by anti-common-cold drugs |
|  | T487 | Poisoning by oth & unsp agents prim acting on the resp sys |
|  | T490 | Poisoning by local antifung anti-infec & anti-inflam drg NEC |
|  | T495 | Poisoning by topical ophthalmological drugs and preparations |
|  | T496 | Poisoning by topical otorhinolaryngological drugs and preps |
|  | T499 | Poisoning by topical agent unspecified |
|  | T788 | Other adverse effects not elsewhere classified |
|  | T789 | Adverse effect unspecified |
|  | T880 | Infection following immunization |
|  | T881 | Other complications following immunization NEC |
|  | T887 | Unspecified adverse effect of drug or medicament |
| **Other bacterial infections 32** | A20 | Plague |
|  | A21 | Tularaemia |
|  | A22 | Anthrax |
|  | A23 | Brucellosis |
|  | A24 | Glanders and melioidosis |
|  | A25 | Rat-bite fevers |
|  | A26 | Erysipeloid |
|  | A27 | Leptospirosis |
|  | A28 | Other zoonotic bacterial diseases not elsewhere classified |
|  | A30 | Leprosy [Hansen's disease] |
|  | A31 | Infection due to other mycobacteria |
|  | A32 | Listeriosis |
|  | A33X | Tetanus neonatorum |
|  | A34X | Obstetrical tetanus |
|  | A35X | Other tetanus |
|  | A36 | Diphtheria |
|  | A37 | Whooping cough |
|  | A38X | Scarlet fever |
|  | A42 | Actinomycosis |
|  | A43 | Nocardiosis |
|  | A44 | Bartonellosis |
|  | A480 | Gas gangrene |
|  | A483 | Toxic shock syndrome |
|  | A484 | Brazilian purpuric fever |
|  | A488 | Other specified bacterial diseases |
|  | A49 | Bacterial infection of unspecified site |
|  | A65X | Nonvenereal syphilis |
|  | A66 | Yaws |
|  | A67 | Pinta [carate] |
|  | A68 | Relapsing fevers |
|  | A69 | Other spirochaetal infections |
|  | A70X | Chlamydia psittaci infection |
|  | A71 | Trachoma |
|  | A74 | Other diseases caused by chlamydiae |
|  | A75 | Typhus fever |
|  | A77 | Spotted fever [tick-borne rickettsioses] |
|  | A78X | Q fever |
|  | A79 | Other rickettsioses |
|  | B95 | Strep and staph as cause of dis classified other chapters |
|  | B96 | Oth bact agents as cause of dis classified to oth chapters |
| **Other viral infections 33** | A90X | Dengue fever [classical dengue] |
|  | A91X | Dengue haemorrhagic fever |
|  | A92 | Other mosquito-borne viral fevers |
|  | A93 | Other arthropod-borne viral fevers not elsewhere classified |
|  | A94X | Unspecified arthropod-borne viral fever |
|  | A95 | Yellow fever |
|  | A96 | Arenaviral haemorrhagic fever |
|  | A98 | Other viral haemorrhagic fevers not elsewhere classified |
|  | A99X | Unspecified viral haemorrhagic fever |
|  | B00 | Herpesviral [herpes simplex] infections |
|  | B01 | Varicella [chickenpox] |
|  | B02 | Zoster [herpes zoster] |
|  | B03X | Smallpox |
|  | B04X | Monkeypox |
|  | B05 | Measles |
|  | B06 | Rubella [German measles] |
|  | B07X | Viral warts |
|  | B08 | Oth viral inf characterized skin / mucous membr les NEC |
|  | B09X | Unspec viral inf characterized skin / mucous membr lesions |
|  | B25 | Cytomegaloviral disease |
|  | B26 | Mumps |
|  | B27 | Infectious mononucleosis |
|  | B33 | Other viral diseases not elsewhere classified |
|  | B34 | Viral infection of unspecified site |
|  | B97 | Viral agents as cause of dis class to other chapters |
| **Other mycoses 34** | B35 | Dermatophytosis |
|  | B36 | Other superficial mycoses |
|  | B37 | Candidiasis |
|  | B38 | Coccidioidomycosis |
|  | B39 | Histoplasmosis |
|  | B40 | Blastomycosis |
|  | B41 | Paracoccidioidomycosis |
|  | B42 | Sporotrichosis |
|  | B43 | Chromomycosis and phaeomycotic abscess |
|  | B44 | Aspergillosis |
|  | B45 | Cryptococcosis |
|  | B46 | Zygomycosis |
|  | B47 | Mycetoma |
|  | B48 | Other mycoses not elsewhere classified |
|  | B49X | Unspecified mycosis |
| **Other protozoal infections 35** | B50 | Plasmodium falciparum malaria |
|  | B51 | Plasmodium vivax malaria |
|  | B52 | Plasmodium malariae malaria |
|  | B53 | Other parasitologically confirmed malaria |
|  | B54X | Unspecified malaria |
|  | B55 | Leishmaniasis |
|  | B56 | African trypanosomiasis |
|  | B57 | Chagas' disease |
|  | B58 | Toxoplasmosis |
|  | B60 | Other protozoal diseases not elsewhere classified |
|  | B64X | Unspecified protozoal disease |
| **Infections of pregnancy and puerperium 36** | O030 | Incomplete spont abort comp by genital tract & pelvic infec |
|  | O035 | Complete or unsp spont abort comp by gen tract & pelvic infn |
|  | O050 | Incomplete other abortion comp by genital tract & pelvic inf |
|  | O055 | Complete/unsp other abortion comp by gen tract & pelvic infn |
|  | O060 | Incomplete unspec abortion comp by genital tract & pelvic in |
|  | O065 | Unspec abort complete/unspec comp by gen tract & pelvic inf |
|  | O070 | Failed medical abortion complic by genital tract/pelvic infn |
|  | O075 | Oth and unsp faild attempt abort comp gen tract/pelvic inf |
|  | O080 | Gen tract and pelv infect follow abort/ectop and molar preg |
|  | O040 | Incomplete med abort comp by genital tract & pelvic infec |
|  | O045 | Complete or unspec med abort comp by gen tract & pelvic infn |
|  | O23 | Infections of genitourinary tract in pregnancy |
|  | O411 | Infection of amniotic sac and membranes |
|  | O753 | Other infection during labour |
|  | O85X | Puerperal sepsis |
|  | O86 | Other puerperal infections |
|  | O910 | Infection of nipple associated with childbirth |
|  | O911 | Abscess of breast associated with childbirth |
|  | O912 | Nonpurulent mastitis associated with childbirth |
|  | O98 | Mat infect and parasitic dis EC comp preg/childbirth/puerp |
| **Perinatal infections 37** | P002 | Fetus and newborn affected by mat infect and parasitic dis |
|  | P027 | Fetus and newborn affected by chorioamnionitis |
|  | P23 | Congenital pneumonia |
|  | P35 | Congenital viral diseases |
|  | P36 | Bacterial sepsis of newborn |
|  | P37 | Other congenital infectious and parasitic diseases |
|  | P38X | Omphalitis of newborn with or without mild haemorrhage |
|  | P39 | Other infections specific to the perinatal period |
| **ID treatment and prevention 38** | B94 | Sequelae of oth and unspec infectious and parasitic dis |
|  | Z030 | Observation for suspected tuberculosis |
|  | Z21X | Asymptomatic human immunodef virus [HIV] infect status |
| **Other IDs 39** | B65 | Schistosomiasis [bilharziasis] |
|  | B66 | Other fluke infections |
|  | B67 | Echinococcosis |
|  | B68 | Taeniasis |
|  | B69 | Cysticercosis |
|  | B70 | Diphyllobothriasis and sparganosis |
|  | B71 | Other cestode infections |
|  | B72X | Dracunculiasis |
|  | B73X | Onchocerciasis |
|  | B74 | Filariasis |
|  | B75X | Trichinellosis |
|  | B76 | Hookworm diseases |
|  | B77 | Ascariasis |
|  | B78 | Strongyloidiasis |
|  | B79X | Trichuriasis |
|  | B80X | Enterobiasis |
|  | B81 | Other intestinal helminthiases not elsewhere classified |
|  | B82 | Unspecified intestinal parasitism |
|  | B83 | Other helminthiases |
|  | B85 | Pediculosis and phthiriasis |
|  | B86X | Scabies |
|  | B87 | Myiasis |
|  | B88 | Other infestations |
|  | B89X | Unspecified parasitic disease |
|  | B94 | Sequelae of oth and unspec infectious and parasitic dis |
|  | B99X | Other and unspecified infectious diseases |
|  | E033 | Postinfectious hypothyroidism |
|  | E321 | Abscess of thymus |
|  | F024 A | Dementia in human immunodef virus [HIV] disease |
|  | F071 | Postencephalitic syndrome |
|  | I88 | Nonspecific lymphadenitis |
|  | T64X | Toxic effect of aflatoxin and other mycotoxin food contams |

# Area: infections – Blood borne viruses (HIV, Hepatitis B and Hepatitis C)

### (Health Protection Scotland HSP records)

### Background

The Sexual Health and Blood Borne Virus Framework

(<http://www.scotland.gov.uk/Publications/2011/08/24085708/0>) sets out the Scottish Government’s agenda for tackling blood borne viruses (BBV; namely HIV, hepatitis C and hepatitis B). The Framework adopts an outcome based approach to improve health and wellbeing in Scotland, with a strong focus on challenging inequalities. Understanding the ethnic variation associated with BBV infections in Scotland is crucial to inform the development of testing, treatment and care services and thus achieve the outcomes listed in the Framework, specifically in respect of reducing the health inequalities gap and that people affected by BBV lead longer, healthier lives. The planned linkage of BBV databases in SHELS4 will therefore provide key data on ethnicity and country of birth (otherwise unavailable), to inform Government, NHS Boards and other stakeholders on the delivery of BBV services in Scotland.

### Preliminary analysis

1. Exploration of linkage rate to the CHI of each BBV dataset using ethnicity and country of birth information available within each dataset
2. Exploration of linkage rate to the Census (as explained in 3.3c) generally as well as using ethnicity derived from Onomap.
3. Descriptive analysis (as explained in 4.2)

### Objectives

1. Explore ethnic inequalities in diagnosis prevalence, in diagnosis incidence, in late diagnosis incidence, in attendance at specialist services and in antiviral treatment in following conditions*:

- Hepatitis C
- HIV
- Hepatitis B

*when the data are available; the reference group being White Scottish

1. Analyse by sex if numbers allow and examine the potential effect of available covariates (see next page)
2. Repeat the analysis and explore differences by country of birth.

### Outcomes

1. 1st diagnosis disease-specific

- Prior to Census (up to April 2001) to get prevalent cases
- Over study period (1/5/2001 - 30/4/2013 or latest reliable date)
- In the community vs. in the hospital/routine screen

1. Late diagnosis disease-specific (identified by adverse disease related outcome, defined below in tables)
2. Attendance at specialist services (HCV and HIV)

- Proportion attending specialist services within 1 year of initial diagnosis
- Time to attendance: Hazard Ratios of attendance within 1 year of initial diagnosis (censored at 1 year) or any time after initial diagnosis (complete follow-up period)

1. Treatment with antiviral therapy (HCV)

- Time to therapy: Hazard Ratios of initiation on therapy within 1 year of attending specialist services (censored at 1 year) or any time after attending specialist services (complete follow-up period)

### Main covariates (sex stratified)

- Age

Socioeconomic variables which will be added in the regression analysis if assessed as having a consistent and positive association with the outcome across ethnic group:

For population of any age:

- Area based socioeconomic status: Scottish Index of Multiple Deprivation (SIMD)
- House ownership
- Highest qualification (household)

We will also investigate the use of a combined individual and household level education where the individual level of education is used for people aged 16-74 and the household level for children and elderly.

For adult population (restricted to 16-74 years old):

- Highest qualification (individual)
- Economy activity in the previous week of census completion

Other covariates:

- Risk group (available only for BBV diagnosed cases)
- Urban/rural indicator
- Health board (grouped either as GGC/other or 4 largest NHS Boards/other)

## Project analysis plan – stage 5a: Prevalence and Incidence of diagnosis with Hepatitis C, HIV and Hepatitis B

**(1) Prevalence and Incidence of BBV diseases diagnosed by ethnicity and sex**

| **Background** | See above. |
| --- | --- |
| **Aims** | Establish the pattern of ethnic differences in the prevalence and incidence of being diagnosed for Hepatitis C, HIV and Hepatitis B in Scotland.  Generate the prevalence rate ratios (PRR) and relative risks of being diagnosed with Hepatitis C, HIV and Hepatitis B for the linked Census population in each ethnic group stratified by sex and adjusted for covariates (e.g age, socio-economic status, health board).  Repeat by country of birth instead of ethnicity. |
| **Hypotheses** | 1. There are ethnic variations in prevalence and incidence of getting diagnosed with Hepatitis C, HIV and Hepatitis B. We hypothesise these vary between specific ethnic group and White Scottish.  2. At the time of first diagnosis, compared to White Scottish people, people from ethnic minority groups have a different proportion of being diagnosed in the community or in hospital setting.  3. These variations cannot be explained by available covariates (e.g age, socio-economic status, health board).  4. Similar hypothesis with COB |
| **Data and ICD codes** | Linkage census database, BBV databases and ACaDMe  3 linked datasets for :   - Hepatitis C (HCV) - HIV - Hepatitis B (HBV) |
| **Numerator** | (a) Prevalence (for HCV, HIV): People diagnosed disease-positive for a specific disease prior to the Census (up to April 2001)  (b) Incidence (for HCV, HIV, HBV): People diagnosed disease-positive for a specific disease between May 2001 – April 2013 (or latest reliable date)  (c) Further analysis: People diagnosed in the community (GP, other) versus in the hospital/routine screen  Definition of diagnosed disease-positive:  - For HCV: PCR positive and/or Antibody positive  - For HBV: HBV surface Antigen positive |
| **Denominator** | (a) Linked 2001 census population (N)  (b) Linked 2001 census population  Person-Years (PY) excluding those who have already been diagnosed prior to the Census and adjusted for deaths, leaving NHS Scotland and incident event.  No age restriction (except for HIV, database confined to age >14)  (c) All people diagnosed disease-positive for a specific disease and linked to Census. |
| **Tabulation** | For each diagnosis: by sex and ethnicity |
| **Analysis** | For each diagnosis, by sex:  1- By ethnicity:  - Number of cases (Total, prior Census 2001, since Census 2001)  - Tabulate for age, socio-economic status and health board  - Poisson regression of number of cases (with confidence intervals and p-values) adjusted for age and other relevant covariates.  Interaction will be considered if numbers allow.  Goodness of fit statistics  2- By COB  Same type of analysis as 1 |
| **Adjust/stratify** | Explore the effect of available covariates such as socio-economic factors and health board. |
| **Additional comments** | Further analysis: Information on the HIV database on location (community vs other) very questionable. For HBV, antenatal screening could either be in the community or in a hospital setting.  Consider analysing changes over calendar time for (b) and calendar year of diagnosis for (c), if numbers allow. |

## Project analysis plan – stage 5b: Incidence of late diagnosis with Hepatitis C, HIV and Hepatitis B

**(1) Incidence of BBV diseases diagnosed late by ethnicity by age and sex**

| **Background** | See above. |
| --- | --- |
| **Aims** | Establish the pattern of ethnic differences in the incidence of being diagnosed late for Hepatitis C, HIV and Hepatitis B in Scotland.  Generate the relative risk of being diagnosed late with Hepatitis C, HIV and Hepatitis B for the linked Census population and for those diagnosed, in each ethnic group stratified by sex and adjusted for covariates (e.g age, socio-economic status, health board).  Repeat by country of birth instead of ethnicity. |
| **Hypotheses** | 1. There are ethnic variations in the incidence of getting diagnosed late with Hepatitis C, HIV and Hepatitis B. We hypothesise these vary between specific ethnic group and White Scottish.  2. These variations cannot be explained by available covariates (e.g age, socio-economic status, health board).  3. Similar hypothesis with COB |
| **Data and ICD codes** | Linkage census database, BBV databases and ACaDMe  3 linked datasets for :   - Hepatitis C (HCV) - HIV - Hepatitis B (HBV) |
| **Numerator** | (a) Incidence (for HCV, HIV, HBV): People diagnosed late for a specific disease between May 2001 – April 2013 (or latest reliable date)  (b) Incidence for any case diagnosed (for HCV, HIV): People diagnosed late for a specific disease at any time point  Definition of late diagnosis:  - For HCV: diagnosed HCV-positive and having an end-stage liver disease (ESLD)-related hospitalization and/or death within either 1 or 2* years of diagnosis  - For HIV: diagnosed HIV positive and having either (a) a low CD4-cell count or (b) a record of disease progression to AIDS, at the time of diagnosis (i.e. within 1 month of diagnosis) - For HBV: diagnosed HBV-positive and having an ESLD-related hospitalization and/or death within either 1 or 2* years of diagnosis  *The option of 2 years was included here to help increase the numbers involved. |
| **Denominator** | People diagnosed disease-positive for a specific disease and linked to Census:  (a) For diagnosis between May 2001 and April 2013 (or latest reliable date)  (b) For all diagnosis (anytime)  PY calculated over the period of late diagnosis identification (1 month, 1 or 2 years) and adjusted for death, leaving NHS Scotland and late diagnosis.  For HIV, with valid information on CD4-cell count at time of HIV diagnosis. |
| **Tabulation** | For each diagnosis: by sex and ethnicity |
| **Analysis** | For each diagnosis, by sex:  1- By ethnicity:  - Number of cases (since Census 2001)  - Tabulate for age, socio-economic status and health board  - Poisson regression of number of cases (with confidence intervals and p-values) adjusted for age and other relevant covariates.  Interaction will be considered if numbers allow.  Goodness of fit statistics  2- By COB  Same type of analysis as 1 |
| **Adjust/stratify** | Explore the effect of available covariates such as socio-economic factors and health board. |
| **Additional comments** | For HBV and HCV, explore the possibility of defining a late diagnosis within 2 years of initial diagnosis if numbers are low within 1 year of initial diagnosis.  Only 1 late diagnosis definition (ie within 1 or 2 years) will be disclosed depending on numbers.  Consider analysing changes over calendar time. |

## Project analysis plan – stage 5c: Attendance at specialist services for Hepatitis C and HIV

**(1) Attendance at specialist services for HCV and HIV by ethnicity and sex**

| **Background** | See above. |
| --- | --- |
| **Aims** | Establish the pattern of ethnic differences in attendance at specialist services for those diagnosed with Hepatitis C and HIV in Scotland, and uptake of antiviral therapy for those attending services for Hepatitis C in Scotland  Generate proportions and hazard ratios of attending specialist services within 1 year of original diagnosis when being diagnosed with Hepatitis C or HIV, in each ethnic group stratified by sex and adjusted for covariates (e.g age, socio-economic status, health board).  Generate proportions and hazard ratios of initiation onto antiviral therapy within 1 year from attendance at specialist service for Hepatitis C, in each ethnic group stratified by sex and adjusted for covariates (e.g age, socio-economic status, health board).  Repeat by country of birth instead of ethnicity. |
| **Hypotheses** | 1. There are ethnic variations in attending specialist services, and uptake of antiviral therapy, when diagnosed with Hepatitis C and HIV. We hypothesise these vary between specific ethnic group and White Scottish.  2. These variations cannot be explained by available covariates (e.g age, socio-economic status, health board).  3. Similar hypothesis with COB |
| **Data and ICD codes** | Linkage census database, BBV databases and ACaDMe  2 linked datasets for :   - Hepatitis C (HCV) - HIV |
| **Numerator** | (a) People diagnosed who attended specialist services within 1 year of initial diagnosis  (b) People diagnosed with chronic HCV who were initiated on antiviral therapy within 1 year of attendance |
| **Denominator** | (a) People diagnosed disease-positive (anytime) for a specific disease and linked to Census  No age restriction (except for HIV, database confined to age >14)  See further exclusions, under additional comments below.  (b) People diagnosed with chronic HCV who attended specialist services  Censored for deaths and migrations |
| **Tabulation** | For each diagnosis: by sex and ethnicity |
| **Analysis** | 1- By ethnicity:  (a) Proportion of people who attended specialist services within 1 year of initial diagnosis  - Poisson regression of number of cases (with confidence intervals and p-values) adjusted for age and other relevant covariates.  Interaction will be considered if numbers allow.  Goodness of fit statistics  (b) Similar analysis to (a).  Explore the effect of available covariates: age, socio-economic status, risk group, urban/rural indicator, NHS board  2- By COB  Same analysis as 1 |
| **Additional comments** | For (a) Confine study populations to periods/health boards with comprehensive data on attendance at specialist services. And will need to do check/review cases where date of diagnosis equates to date of attendance. (Refer to McDonald S, et al. JVH)  - Consider analysing changes over calendar time. |

# Project analysis plan – stage 6: Bowel Cancer Screening

### (Bowel Cancer Screening data and Cancer Registry SMR06)

| **Background** | Inequalities in uptake of bowel cancer screening by ethnicity were observed in pilots in England (UK colorectal cancer screening pilot) but there is no participant level comparable data in Scotland. |
| --- | --- |
| **Aim** | Establish pattern of ethnic differences in bowel cancer screening uptake and outcomes in Scotland. |
| **Hypotheses** | 1. There are ethnic variations in screening uptake in men and women. We hypothesise these to vary in specific ethnic group compared to White Scottish.  A lower uptake in minority ethnic group is expected (observed in South Asian population in England).  2. These variations cannot be explained by available covariates |
| **Data and ICD codes** | Linkage database, Scottish Bowel Screening Programme and Cancer Registry (SMR06) database.  ICD codes for cancer (ICD10). See Table 3 for list of codes. |
| **Numerator** | 1- People screened  2- Positive screening test results (SCRERES codes 3,5,6,8)  3- Colonoscopy performed (completed and not)  4- Pathology detected (polyps, adenoma, cancer (but not including polyp cancer))  For first and second rounds of screening separately (screening data from 2009 – 2013: this will allow data from two complete screening rounds in each Health Board), where the size of the denominators allow. |
| **Denominator** | Within the linked census population:  1- People invited to screening  2- People screened (participants)  3- Positive screening test results  4- Positive screening test results  In addition- Pathology detected (cancer) will be analysed with denominators 1, 2 and 3 as well as people not screened (non-participants), participants with negative screening test  Adjusted for deaths and migrations in the denominator populations. |
| **Tabulation** | For each outcome: Sex and ethnic group  For screening uptake: By CoB [born in Scotland as the reference group) and by religion (Church of Scotland as the ref group). By pilot HB area or not. |
| **Analysis** | For each screening round offered (by individual) separately:   - Screening uptake - Positive screening test results rate - Positive predictive value (PPV) for cancer - Colonoscopy completion rate - pathology detected following colonoscopy (number of polyps, adenomas, crude cancer detection rate, Dukes’ stages A-D, site)   If time and numbers allow (likely to be small):   - False negative rates (screened, with negative test results but cancer is diagnosed before the next screening invitation) and Negative Predictive Value (NPV)   - Number of cases  - Inspect 5-year age band (age at screening) stratified results and rates with standard adjustment for age.  - Report rates or percentages as appropriate  - Report ethnic differences in risk ratio (Poisson) with confidence intervals and p-values, compared to white Scottish (reference population)  Adjust for age if necessary and appropriate covariates |
| **Adjust/stratify** | Explore the effect of a range of available covariates (religion, country of birth and socio-economic factors) |
| **Handling bias, chance and interaction -** See bias notes | |

**Table 3: ID diagnostic groups and associated ICD codes for large bowel (colorectal) cancer (or non-invasive tumours)**

| **ID Group** | **ICD-10 Codes** |
| --- | --- |
| Malignant neoplasm of colon | C18.0-C18.9 |
| Malignant neoplasm of rectosigmoid junction | C19X |
| Malignant neoplasm of rectum | C20X |
| Malignant neoplasm of anus and anal canal | C21.0-C21.8 |
| Carcinoma in situ of colon | D01.0 |
| Carcinoma in situ of rectosigmoid junction | D01.1 |
| Carcinoma in situ of rectum | D01.2 |
| Carcinoma in situ of anus and anal canal | D01.3 |
| Neoplasm of uncertain or unknown behaviour of appendix | D37.3 |
| Neoplasm of uncertain or unknown behaviour of colon | D37.4 |
| Neoplasm of uncertain or unknown behaviour of rectum (or rectosigmoid junction) | D37.5 |

Primary analysis:

- Screening uptake
- Positive screening test results rate
- PPV for cancer

Secondary analysis:

- Colonoscopy completion rate
- pathology detected following colonoscopy (polyps, adenomas, crude cancer detection rate, Dukes’ stages A-D, site)
- False negative rates and Negative Predictive Value

|  |  | **Cancer** | **No Cancer** |  |
| --- | --- | --- | --- | --- |
| **FOB Screen Test outcome** | Positive test | True Positive (TP) | False Positive (FP) | **Positive Predictive value** = TP/ (TP+FP) |
|  | Negative test | False Negative (FN) | True Negative (TN) | **Negative Predictive value** = TN / (TN+FN) |
|  |  | **Sensitivity**  = TP / (TP+FN) | **Specificity**  = TN / (FP+TN) |  |

**Hypothesis ethnic specific:**

Relative to Scottish reference population,

- Screening uptake will be same or higher in Other White British, similar in White Irish and lower in other White, in South Asian (SA) and non-white minority ethnic group.
- Positive predictive value (PPVs) will be lower in some minority ethnic group.
- Colon cancer less common in minority ethnic groups.

**Diagram**

**Denominators Numerators**

- People invited to screening
- People screened
- People invited but not screened
- Positive screening test results
- People with negative screening test
- Colonoscopy
- People screened
- Positive screening test results
- Colonoscopy
- Pathology detected

# Project analysis plan – stage 7: Primary Care risk factors

**7a- CVD outcomes**

| **Background** | We have previously explored ethnic variations in cardiovascular (CVD) outcomes in phase 2. |
| --- | --- |
| **Aim** | Explore whether previously shown ethnic variations in CVD outcomes (phase 2) in White Scottish, Other white British and Pakistani in Scotland change when adjusted for specified risk factors (i.e. smoking, diabetes). |
| **Hypotheses** | There are ethnic variations (eg between Pakistani men and Scottish men) in rates of CVD (all CVD, may possibly look at MI if numbers allow). These variations may change substantially (>10%) on adjustment for smoking, diabetes. |
| **Data and ICD codes** | Linked Census and Primary care databases (phase 3) further merged with:   - CVD hospitalisations & deaths databases (phase 2) |
| **Numerator** | First CVD hospital discharge or death between May 2001 –April 2008 (phase 2 outcome) |
| **Denominator** | Linked 2001 census population to primary care records (around 53,000 people) - Restricted to people aged 30 years old and above  PY at risk |
| **Tabulation** | Sex and ethnic group |
| **Analysis** | For first CVD event:  Definition of risk factors:   - Smoking status: identify “Never smoker” recorded anytime and set as default, update as “Ever smoker” if any “smoker” or “ex-smoker” status is recorded up to April 2008. - Diabetes: any diabetes (excluding gestational) recorded prior to April 2008.   **1. Cross tabulation of primary care risk factors** (complete linked database)  - with Ethnicity  **2. Assess data completeness in relation to CVD** (complete linked database)  - What proportion of patients with both health outcome (any first CVD event) and a primary care record has a record of diabetes by ethnic group?  - What proportion of patients with both health outcome (any first CVD event) and a primary care record has a record of smoking status (ever, never, not known) by ethnic group?  **3. Explaining ethnic variation in outcomes using risk factor data**  - Are ethnic variations in outcomes (CVD risks) altered on adjustment for risk factors (i.e. smoking, diabetes)?  Use count regression method (negative binomial or Poisson if data confirmed as having no extra-Poisson variation) to examine unadjusted estimates (CVD incidence risks) by ethnic group with those adjusted for risk factors. |
| **Adjust/stratify** | Explore the effect of available covariates such as socio-economic factors (include education in the model as for CVD analyses in phase 2) |
| **Handling bias, chance and interaction -** See bias notes | |

**Table 5: Read codes used to define Diabetes and Smoking risk factors**

**4.a. Diabetes**

All the read codes starting with C10 were requested for extraction. They are included in the definition of Diabetes excluding C10K (Type A insulin resistance), C10L (Fibrocalculous pancreatopathy) and C10FS (Maternally inherited diabetes mellitus). No other code related to gestational diabetes (e.g. L1808, 8CE00, Q44B) is included nor was requested for extraction.

**4.b. Smoking status**

All the read codes starting with 137 were extracted. Smoking status includes all 137 codes as classified in the table below:

| **Read Code** | **Title** | **Classified as** |
| --- | --- | --- |
| 1371 | Never smoked tobacco | Never smoker |
| 1372 | Trivial smoker - < 1 cig/day | Smoker |
| 1373 | Light smoker 1-9 cigs/day | Smoker |
| 1374 | Moderate smoker 10/19 cigs/day | Smoker |
| 1375 | Heavy smoker 20-39 cigs/day | Smoker |
| 1376 | Very heavy smoker 40+ cigs/day | Smoker |
| 1377 | Ex-Trivial smoker - < 1 cig/day | Ex-smoker |
| 1378 | Ex-Light smoker 1-9 cigs/day | Ex-smoker |
| 1379 | Ex-Moderate smoker 10/19 cigs/day | Ex-smoker |
| 137A | Ex-Heavy smoker 20-39 cigs/day | Ex-smoker |
| 137B | Ex-Very heavy smoker 40+ cigs/day | Ex-smoker |
| 137C | Keeps trying to stop smoking | Smoker |
| 137D | Admitted tobacco cons untrue? | NC* |
| 137E | Tobacco consumption unknown | NC* |
| 137F | Ex-smoker - amount unknown | Ex-smoker |
| 137G | Trying to give up smoking | Smoker |
| 137H | Pipe Smoker | Smoker |
| 137I | Passive smoker | NC* |
| 137I0 | Exposed to tobacco smoke at home | NC* |
| 137J | Cigar smoker | Smoker |
| 137K | Stopped smoking | Ex-smoker |
| 137L | Current non-smoker | Never smoker |
| 137M | Rolls own cigarettes | Smoker |
| 137N | Ex-pipe smoker | Ex-smoker |
| 137O | Ex-cigar smoker | Ex-smoker |
| 137P | Cigarette Smoker | Smoker |
| 137Q | Smoking started | Smoker |
| 137R | Current smoker | Smoker |
| 137S | Ex smoker | Ex-smoker |
| 137T | Date ceased smoking | Ex-smoker |
| 137U | Not a passive smoker | NC* |
| 137V | Smoking reduced | Smoker |
| 137W | Chews tobacco | NC* |
| 137X | Cigarette consumption | Smoker |
| 137Y | Cigar consumption | Smoker |
| 137Z | Tobacco consumption NOS | Smoker |
| 137a | Pipe tobacco consumption | Smoker |
| 137b | Ready to stop smoking | Smoker |
| 137c | Thinking about stopping smoking | Smoker |
| 137d | Not interested in stopping smoking | Smoker |
| 137e | Smoking restarted | Smoker |
| 137f | Reason for restarting smoking | Smoker |
| 137g | Cigarette pack-years | Smoker |
| 137h | Minutes from waking to first tobacco consumption | Smoker |
| 137i | Ex-tobacco chewer | NC* |
| 137j | Ex-cigarette smoker | Ex-smoker |
| 137k | Refusal to give up smoking status | NC* |
| 137l | Ex-roll up cigarette smoker | Ex-smoker |
| 137m | Failed attempt to stop smoking | Smoker |
| 137n | Total time smoked | Smoker |
| 137o | Waterpipe tobacco consumption | NC* |

* NC = Not classified

**7b- All-cause mortality**

| **Background** | We are exploring ethnic variations in all-cause mortality in phase 4. |
| --- | --- |
| **Aim** | Explore whether ethnic variations in all-cause mortality (phase 4) in White Scottish, Other White British and Pakistani in Scotland change when adjusted for specified risk factors (i.e. smoking, diabetes). |
| **Hypotheses** | There are ethnic variations in all-cause mortality rates. These variations may change substantially (>10%) on adjustment for smoking and diabetes. |
| **Data and ICD codes** | Linked Census and Primary care databases (phase 3) further merged with:   - All-cause Mortality database (phase 4) |
| **Numerator** | Death between May 2001 –April 2013 (phase 4 outcome) |
| **Denominator** | Linked 2001 census population to primary care records (around 53,000 people)  PY at risk |
| **Tabulation** | Sex and ethnic group |
| **Analysis** | For all-cause mortality:  Definition of risk factors:   - Smoking status: identify “Never smoker” recorded anytime and set as default, update as “Ever smoker” if any “smoker” or “ex-smoker” status is recorded up to April 2013. - Diabetes: any diabetes (excluding gestational) recorded prior to April 2013.   **1. Cross tabulation of primary care risk factors** (complete linked database)  - with Ethnicity  **3. Explaining ethnic variation in outcomes using risk factor data**  - Are ethnic variations in outcomes (all-cause mortality rates) altered on adjustment for risk factors (i.e. smoking, diabetes)?  Use count regression method (negative binomial or Poisson if data confirmed as having no extra-Poisson variation) to examine unadjusted estimates (all-cause mortality risks) by ethnic group with those adjusted for risk factors. |
| **Adjust/stratify** | Explore the effect of available covariates such as socio-economic factors |
| **Handling bias, chance and interaction -** See bias notes | |

### Appendix A

### Template for output tables for RR

Male

|  |  |  | Age-adjusted RRs | | | | | RRs adjusted for age + other variables | | | |
| --- | --- | --- | --- | --- | --- | --- | --- | --- | --- | --- | --- |
|  | N event | PY at risk | Poisson rates  (for 100,000 PY) | RR | lower CI | upper CI | p | RR | lower CI | upper CI | p |
| White Scottish |  |  |  | 100 | . | . | . | 100 | . | . | . |
| Other White British |  |  |  |  |  |  |  |  |  |  |  |
| White Irish |  |  |  |  |  |  |  |  |  |  |  |
| Other White |  |  |  |  |  |  |  |  |  |  |  |
| Ethnic 5 |  |  |  |  |  |  |  |  |  |  |  |
| Ethnic 6 |  |  |  |  |  |  |  |  |  |  |  |
| Ethnic 7 |  |  |  |  |  |  |  |  |  |  |  |
| … |  |  |  |  |  |  |  |  |  |  |  |
| Other covariate 1 |  |  |  |  |  |  |  |  |  |  |  |
| Other covariate 2 |  |  |  |  |  |  |  |  |  |  |  |
| … |  |  |  |  |  |  |  |  |  |  |  |

Female

|  |  |  | Age-adjusted RRs | | | | | RRs adjusted for age + other variables | | | |
| --- | --- | --- | --- | --- | --- | --- | --- | --- | --- | --- | --- |
|  | N event | PY at risk | Poisson rates  (for 100,000 PY) | RR | lower CI | upper CI | p | RR | lower CI | upper CI | p |
| White Scottish |  |  |  | 100 | . | . | . | 100 | . | . | . |
| Other White British |  |  |  |  |  |  |  |  |  |  |  |
| White Irish |  |  |  |  |  |  |  |  |  |  |  |
| Other White |  |  |  |  |  |  |  |  |  |  |  |
| Ethnic 5 |  |  |  |  |  |  |  |  |  |  |  |
| Ethnic 6 |  |  |  |  |  |  |  |  |  |  |  |
| Ethnic 7 |  |  |  |  |  |  |  |  |  |  |  |
| … |  |  |  |  |  |  |  |  |  |  |  |
| Other covariate 1 |  |  |  |  |  |  |  |  |  |  |  |
| Other covariate 2 |  |  |  |  |  |  |  |  |  |  |  |
| … |  |  |  |  |  |  |  |  |  |  |  |

### Appendix B

### Statistical Disclosure Control Guidance for the Scottish Health and Ethnicity Linkage Study

(Draft revised version July 2014)

These guidelines apply disclosure control principles to outputs from the SHELS data. More general guidance can be obtained from the ESSNet document on SDC on microdata research.^[[1]](#footnote-1)^

### 1. Numbers of persons

1.1 Sub-group sizes and statistics

Many outputs report the number of persons (N) who fall into a given category such as a cell in a frequency table or matrix. The guideline is that if N is 5 or below, it is deleted and replaced by a full stop. If N is 6 or above, its disclosure-controlled value (from now on referred to as its controlled value) is reported. Where N has the value 6 or 7, its controlled value is 10; otherwise its controlled value is the closest multiple of 5. The following table gives controlled values for original values of N from 0 to 17 and the general rule for k > 3:

| Table 1: Original and controlled values | | | | |
| --- | --- | --- | --- | --- |
| Original N | 0 to 5 | 6 to 12 | 13 to 17 | (5k - 2) to (5k + 2) |
| Controlled N | . | 10 | 15 | 5k |

Any statistics (such as means, risk ratios and confidence intervals) are reported unless N is 5 or below in which case they are also replaced by full stops as per the following table:

| Table 2: Publication protocol | |
| --- | --- |
| 0 ≤ N ≤ 5 | Replace both N and any statistics by full stops |
| N = 6 or 7 | Replace N by 10 and report statistics |
| N ≥ 8 | Replace N by the nearest multiple of 5 and report statistics |

1.2 Marginal totals

It is assumed that row and column totals are either reported as part of the table or that they can be calculated from outputs published elsewhere from the same data set. To achieve this while maintaining disclosure, marginal totals of rows, columns and tables will be subject to the same disclosure control as individual cell values.^[[2]](#footnote-2)^

Cell values and marginal totals expressed as percentages will not be released, though controlled values can after their release be expressed as percentages by the researchers.

1.3 Disclosure between tables

If a statistic is suppressed in one table it must not be derivable by comparing data released in any other table or in any other previously released tables. The use of rounded values for publication means that such comparisons cannot be made to the necessary level of precision and hence disclosure checking between tables is not required.

**2. Graphical output**

2.1 Histograms and bar charts (whether simple or stacked) can be reported if, and only if, the same data in frequency table form could be reported. Columns corresponding to cells of fewer than six cases must be deleted.

2.2 In general, graphical output can be used only to make released (or releasable) tabular or other formats more accessible to the user. Hence scatterplots for example are not permitted if they allow the identification of groups of fewer than six persons as this would not be released.

**3. Derived statistics**

3.1 Correlation coefficients can be reported if they are based on at least four degrees of freedom (corresponding to at least six persons).

3.2 Values of test statistics where the number of degrees of freedom is based on the number of microdata records^[[3]](#footnote-3)^ can be reported where the number of degrees of freedom represents at least six persons.

### Appendix C

**Guidelines on the Destruction and Retention of Output**

The SHELS project involved the analysis of a linked data set part of which was taken from results of the 2001 Census and as such it is covered by the legislation governing the use and management of Census data. These guidelines are consistent with that legislation and with Scottish Government practice on data management to ensure that the security of person-level data is secure and is seen to be secure by all parties.

This document concerns the destruction of “output” or “release requests” (i.e. the results of the statistical analysis undertaken by the SHELS researchers). This must be distinguished from the destruction of the data sets themselves which are held in the secure setting at Ladywell House. These will in due course be destroyed after the end of the SHELS project, but this matter is dealt with elsewhere.

Output usually consists of word processor files, spreadsheets and paper hard copy which have been considered and approved by the Disclosure Committee. They can be divided into (i) those which are going into the public domain such as a journal or seminar presentation and (ii) those which are for circulation only within the team of researchers who have signed the confidentiality undertaking. No restrictions apply to anything in the first category. The documents which will be published can be stored and disseminated in any way and no document destruction criteria apply.

Output restricted to the team of researchers will be marked with a heading stating this when it is released from National Records of Scotland. After the researchers have completed their consideration of the output and the analysis to be used has been decided and implemented, all output not intended for publication must be destroyed with the exception of material held in case of post-publication queries. Material in this latter category must be packaged appropriately and transferred to the secure setting in Ladywell House where it will be stored for five years or until the end of the SHELS project.

The destruction of material not to be published or retained should follow the guidance below. After it has been destroyed, confirmation of this should be forwarded to the Principal Investigator using the form on the reverse of this sheet.

Emails:

Delete the emails from Inbox and then from the backup storage.

Word processing and spreadsheet files on hard drives or USB sticks/drives:

Send to Recycle Bin, Wastepaper Basket or equivalent and then empty the Recycle Bin or Wastepaper Basket.

Paper Destructive Procedures:

Destroy using commercially available equipment that meets BS EN 15713:2009. A cross cutting shredder with a cutting width of 4mm or less (BS EN15713: 2009 shred number 6) is recommended. OR:

Destroy by disintegration using a SEAP 8100 approved disintegrator with a mesh size of 6mm or less. OR:

Destroy by incineration using a SEAP 8100 incinerator or a SEAP 8200 approved organisation.

Compact Discs (CDs) Destructive Procedures:

Break/cut the disc into two or more pieces, ensuring that no piece is greater than 50% of the total disc area.

### Output Destruction Form

### Project: Scottish Health and Ethnicity Linkage Study (SHELS)

### Principal Investigator: Prof. Raj Bhopal, University of Edinburgh

**Form completed by :**

**Please clearly indicate your answers by marking ‘🗸’ in the blank boxes.**

| Project topic: Ethnic variations in chest pain and angina in men and women | | | |
| --- | --- | --- | --- |
| **Type of output** | **Method used** | **Output destroyed** | **Reason**  **( if not destroyed)** |
| E-mails | (e.g. deleted from inbox and from backup) |  |  |
| Word files and spreadsheets | (e.g. deleted from directory and from recycle bin) |  |  |
| Hard copy paper drafts | (e.g. shredded) |  |  |
| Manuals |  |  |  |
| Letters |  |  |  |
| CDs and DVDs | (e.g. cut into two) |  |  |

**Mention any other source destroyed.**

| **Type of output** | **Method used** | **Output destroyed** | **Reason**  **( if not destroyed)** |
| --- | --- | --- | --- |
|  |  |  |  |
|  |  |  |  |
|  |  |  |  |

**Please sign and forward (a paper or scan copy) of the checklist to Prof. Raj Bhopal, Principal Investigator, SHELS Project.**

**Destroyed by: Signature:**

**(Name in Capitals)**

**Full Address: Date:**

**E-mail id:**

**Phone no.:**

### Appendix D

### Content of census and health related data

**Contents of the Census data**

| **Fields:**  Ethnic group  Religion, current  Religion of upbringing  Country of birth  Age  Sex  Long term illness  Self assessed health  Marital status  Labour force status  Socioeconomic status  Highest qualification  Scottish Index of Multiple Deprivation deciles  Car ownership  Housing tenure  Household size  Number of rooms  Urban/rural indicator  Health board (Glasgow, Lothian, Tayside, Other)  Mobile (temporary) accommodation  Self-contained accommodation  Central heating  Moved within last year  Economic activity last week  Occupation – will be requested from NRS either as a variable based on Standard Industrial Classification or a calculated variable for Occupational risk factor group |
| --- |

**Contents of Health Related Data for NRS deaths records (all cause mortality)**

| **Fields:**  Encrypted CHI*  Date of death  Primary and secondary causes of death |
| --- |

*NB. Encrypted CHI is only used by ISD staff to link the health and Census data and is then removed from the analyses files that are used by the SHELS research team.

**Contents of Health Related Data for SMR01/Deaths – All Cause Hospitalisations**

| **Fields:**  Encrypted CHI*  Age in years  Sex  Admission date  Admission type  Admission reason  Duration of hospital admission  Days waiting  Main condition (for readmission analysis per disease)  Date of discharge  Discharge type  Date of death  Causes of death - primary and secondary |
| --- |

NB: Calculated fields such as length of stay and episode markers will be added to the extracted dataset by ISD linkage team

**Contents of Health Related Data for SMR01/Deaths for Injuries and Poisoning and all Infectious Diseases**

| **Fields:**  Encrypted CHI  Age in years  Sex  Admission date  Admission type  Admission reason  Duration of hospital admission  Main condition (all ICD 9&10 codes for infectious diseases; injuries/poisoning codes)  Other condition (all ICD 9&10 codes for infectious diseases; injuries/poisoning codes)  Date of discharge  Discharge type  Date of operation  Operation code  Inpatient/day case marker  Date of death  Causes of death - primary and secondary (all death following hospitalisation as well as disease-specific death) |
| --- |

NB: Calculated fields such as length of stay and episode markers will be added to the extracted datasets by ISD linkage team

**Contents of Health Related Data for HPS databases:** (The HepB, HepC and HIV datasets at HPS cover different time periods and do not all contain the same data variables, due to the differing epidemiology and clinical management of each disease).

**Hepatitis B/SMR01/Deaths**

| **Fields:**  Encrypted CHI  Sex  Age at diagnosis (years)  NHS Board of residence (at diagnosis)  Date of earliest HBsAg positive specimen (MMYYYY)  Source of 1^st^ positive test (hospital, routine/antenatal screen, GP, other community setting)  HBV test result (recent/acute, chronic)  Ethnicity assigned from Onomap (incomplete)  Date of late diagnosis (MMYYYY)  Late diagnosis indicator  Date of migration (MMYYYY)  Date of death (MMYYYY) |
| --- |

NB: Ethnicity from the HPS data (where available) will be used to check validity of current reports for the Scottish Government by comparing to similar tables using self-assigned ethnicity from the 2001 Census.

**Contents of Health Related Data for HPS databases:**

**Hepatitis C/SMR01/Deaths**

| **Fields:**  Encrypted CHI  Sex  Age at diagnosis (years)  NHS Board of residence (at diagnosis)  Date of earliest positive specimen (MMYYYY)  Source of 1^st^ positive HCV test (hospital, routine screen, GP, other community setting)  HCV test result (recent/acute, chronic, resolved)  Description of result  HCV genotype  Risk group  Date of first attendance (specialist services) (MMYYYY)  Time from diagnosis to 1^st^ attendance (specialist services) (day)  Date started antiviral therapy (MMYYYY)  Time from diagnosis to start of antiviral therapy (day)  Response to antiviral therapy (sustained viral response (SVR), non-SVR)  Late diagnosis date (MMYYYY)  Late diagnosis indicator  Date of death (MMYYYY)  Date of migration (MMYYYY) |
| --- |

NB. Country of Birth in the HepC database is very incomplete, ethnicity is not available, hence not included in this dataset.

**Contents of Health Related Data for HPS databases:**

**HIV Diagnoses/SMR01/Deaths**

| **Fields:**  Encrypted CHI  Sex  Age at diagnosis (year)  NHS Board of residence (at diagnosis)  Date of earliest positive specimen (MMYYYY)  Source of HIV diagnosis (hospital, routine screen, GP, other community setting)  Risk group  Country of birth (incomplete prior to 2007)  Ethnicity (incomplete)  Date of AIDS diagnosis (symptoms) (MMYYYY)  New case (Known in Scotland or elsewhere/New to Scotland/Unknown)  Infected outside to Scotland (Yes/No)  Follow up status (Attending/Not attending/Lost/Dead/Left Scotland/Recent)  Date last attended healthcare services  Date of 1^st^ attendance in HIV specialist care (MMYYYY)  Time from diagnosis to 1^st^ attendance in HIV specialist care (day)  Date of 1^st^ attendance for CD4 measurement (MMYYYY)  Time from diagnosis to 1^st^ attendance for CD4 measurement (day)  1^st^ CD4 result (category: low, medium, high)  Date of late diagnosis (MM YYYY)  Late diagnosis indicator  Date of death (MMYYYY)  Date of migration (MMYYYY) |
| --- |

NB: Country of birth/Ethnicity from the HPS data (where available) will be used to check validity of current reports for the Scottish Government by comparing to similar tables using self-assigned ethnicity from the 2001 Census.

**Contents of Health Related Data for Scottish Bowel Cancer Screening Program**

| **Fields:**  Encrypted CHI  Date screening test kit sent to participant  Health Board of Residence  Screening round  Sex  Age in years  Screening test result  Flag for kit completed in error  Health Board identifier/code  Date of notification of a screening result  Colonoscopy performed  Date colonoscopy performed  Reason for not having a colonoscopy  Colonoscopy completed  Invasive cancer detected  ICD-10 classification of neoplasm  Tumour classification (after surgery)  Nodal classification (after surgery)  Metastases classification (after surgery)  TNM derived Dukes’ stage  Polyps detected  Adenoma detected  Count of adenomas  Maximum dimension of the largest adenoma  Polyp cancer detected  Polypectomy performed at colonoscopy  Complication from the colonoscopy requiring admission  Death |
| --- |

**Contents of Health Related Data for SMR06 (Cancer Registry) for linking to Bowel Cancer Screening dataset**

| **Fields:**  Encrypted CHI  Date of incidence/Incidence date  Site ICD9  ICD10 Cancer site  ICDO2  Type ICD 03  Grade cell type  Stage colorectal |
| --- |

| Contents of Health Related Data for Primary Care Risk Factor Data  (from SHELS phase 3 covered by ethical approval reference 11/MRE00/4) | |
| --- | --- |
| Full Field name | English description |
| Encrypted CHI |  |
| Date of registration | First date of patient registration in practice |
| Date of deregistration | Date of last deregistration or death |
| Tobacco consumption | All recording of smoking codes |
| Tobacco consumption Date | Date/time variable |
| Family history of disease | All recordings of family history of disease |
| Family history of disease date | Date/time variable |
| Exercise | All recording of exercise status |
| Exercise Date | Date/time variable |
| Diabetes | All diagnoses of diabetes |
| Diabetes Date | Date/time variable |
| CHD | All diagnoses of Coronary Heart Disease  (including Angina) |
| CHD Date | Date/time variable |
| Stroke | All diagnoses of Stroke & Transient Ischaemic Attack |
| Stroke Date | Date/time variable |
| Atrial Fibrillation | All diagnoses of Atrial fibrillation |
| AF Date | Date/time variable |
| Statins | All prescriptions of Lipid lowering therapies |
| Statins Date | Date/time variable |
| Height | All values or measurements |
| Height date | Date/time variable |
| Weight | All values or measurements |
| Weight date | Date/time variable |
| Cholesterol | All values or measurements |
| Cholesterol Date | Date/time variable |
| Systolic_blood_pressure | All values or measurements |
| Systolic_blood_pressureDate | Date/time variable |
| Diastolic_blood_pressure | All values or measurements |
| Diastolic_blood_pressureDate | Date/time variable |
| Asthma | All diagnosis of asthma |
| Asthma date | Date/time variable |
| Asthma prescriptions | All prescriptions of inhaled corticosteroids (BNF) |
| Asthma prescriptions date | Date/time variable |

### Appendix E

Recommended terminology for use in papers

| **Reasons for multiplying RRs by 100** | Results are then percentages and it is easier for interpretation by readers of papers as fewer decimal points are used. |
| --- | --- |
| **Results should be labelled rate or risk ratios** | Rate ratios are when incidence rates are calculated using the person time denominator. Risk ratios are the outcome of incidence rates calculated using the population denominator (also known as the cumulative incidence rate or, synonymously, cumulative incidence proportion).  If the ratios relate to prevalence data, and not incidence data, the correct phrase is prevalence ratio, or synonymously, prevalence rate ratio or prevalence proportion ratio-the latter being, strictly, the correct phrase)  For analysis using proportions eg readmissions and bowel cancer screening outcomes, results should be labelled risk ratios. |
|  |  |

1. Brandt M., Franconi L., Guerke C., Hundepool A., Lucarelli M., Mol J., Ritchie F., Seri G. and Welpton R. (2010), *Guidelines for the checking of output based on microdata research*, Final report of ESSnet sub-group on output SDC. Available at http://securedata.data-archive.ac.uk/media/11679/essnet_sdc.pdf [↑](#footnote-ref-1)
2. The controlled marginal totals will often not equal the sums of the controlled cell values of which they are composed. Overall however the controlled values will remain as close to the original data as possible while maintaining disclosure control. [↑](#footnote-ref-2)
3. This is to prevent reporting of test statistics with few degrees of freedom where this indicates few microdata records. It does not for example cover the chi-square test for independence in a contingency table. This could be disclosed since the number of degrees of freedom depends on the number of cells in the table, not on the numbers of observations in those cells, and it is the latter which are determined by the numbers of microdata records. [↑](#footnote-ref-3)
