# Supplementary material for: Mortality, ethnicity, and country of birth on a national scale, 2001–2013: A retrospective cohort (Scottish Health and Ethnicity Linkage Study)
Source: PLoS Med. 2018 Mar 1;15(3):e1002515. doi: 10.1371/journal.pmed.1002515 (PMC5832197; doi:10.1371/journal.pmed.1002515)
Supplement: S1 Appendix — (DOCX) [file pmed.1002515.s003.docx]

**S1 Appendix**

**Supplementary methods and data file.**

**1 Details on linkage methods**

Fig A, republished from our open-access publication, together with this edited version of the linkage methods [1], illustrates how linkage was based on information from three datasets: healthcare records, which include personal identifiers and clinical information; the CHI (Community Health Index) file which contains personal identifiers and the CHI number; and the census file which contains the census number, personal identifiers and details of individuals’ ethnicity (and many other characteristics). The CHI dataset lists everyone in Scotland registered with a general practitioner or eligible for NHS screening services and forms a unique identifier for NHS use. More than 99% of the Scottish population is estimated to be listed on the CHI. Date of birth, sex, surname and forename (using soundex codes to allow for variations in spelling), address and full postcode, available in Census and CHI, were used to link the census number to the CHI number. For the records deemed to be matches, 73.6% were exact matches on the 6 linking variables. For the remainder, a probability matching process was performed. At this stage, no other variables were in the dataset. The number of people with a valid ethnic group code and the linkage rate, with the estimated total population, are shown in Table A.

Methods have been developed to identify how false positives occur and what kind of strategies a human checker employs to decide whether a pair match is ‘good’. These decision strategies were built into a ‘partitioning’ computer algorithm. These ‘partitions’ then allow the allocation of effort to the most profitable ‘partitions’ which yield the lowest false-positive and highest true-positive rates. Using methods previously described we estimated an upper limit to the false-positive linkage rate of 0.08% [1].

CHI and the census numbers were encrypted before linkage to other datasets. A one-way cryptographic (‘hashing’) algorithm was used to encrypt the CHI number. The census number was encrypted using an algorithm developed by NRS (National Records of Scotland). Once the linkage was completed personal identifying variables (such as names, address, postcode and dates of birth) were removed leaving a file with an encrypted CHI number and its corresponding encrypted census number (look up file).

A census extract containing ethnic group (and limited other data including age, sex, country of birth and indicators of socio-economic status) was joined to the above look-up file using the encrypted census number. The encrypted census numbers were then discarded, leaving ethnicity and other variables from the census, the encrypted CHI number and a newly generated index number unrelated to other numbers for the exclusive use of this project. The relevant parts of the database of hospitalisations and deaths held at ISD were linked via the encrypted CHI numbers. The encrypted CHI was replaced with an unrelated serial number (to keep together the multiple records on the same people), resulting in depersonalised clinical health records carrying census derived ethnicity codes and other relevant census data.

**Fig A. Overview of Record Linkage Process.**


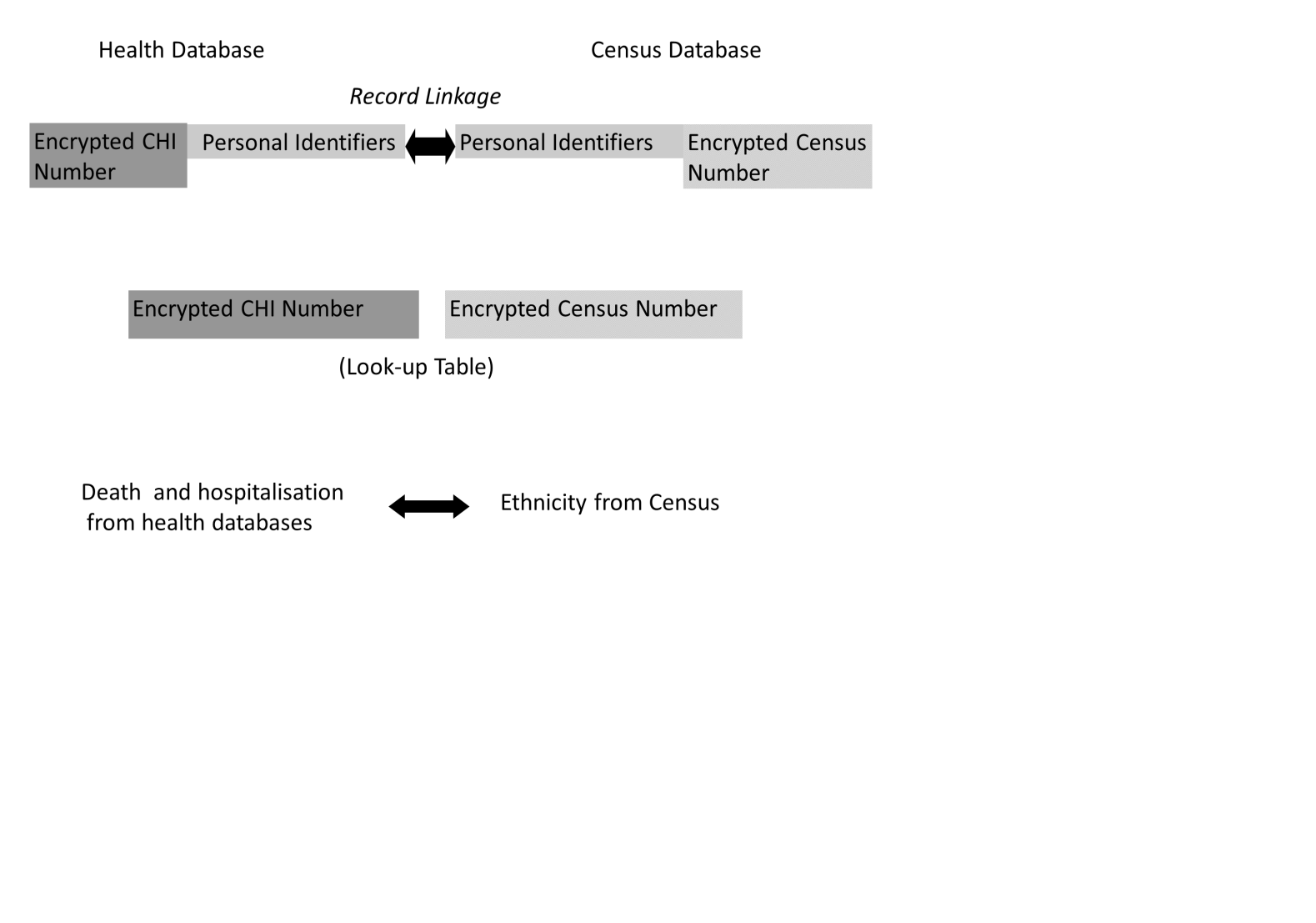


**Table A. Linkage proportions of 2001 Census to Community Health Index (CHI) by ethnic group in relation to total population in census and estimated for Scotland**

|  | **Ethnicity** | **Number of people completing census** | **Percentage** |
| --- | --- | --- | --- |
| 1 | White Scottish | 4290153 | 95.3 |
| 2 | Other White British | 357788 | 93.6 |
| 3 | White Irish | 47173 | 92.2 |
| 4 | Other White | 74655 | 87.9 |
| 5 | Any Mixed Background | 12117 | 91.7 |
| 6 | Indian | 13717 | 89.9 |
| 7 | Pakistani | 28538 | 89.8 |
| 8 | Bangladeshi | 1783 | 88.0 |
| 9 | Other South Asian | 5810 | 85.1 |
| 10 | Caribbean | 1659 | 89.5 |
| 11 | African | 4514 | 86.5 |
| 12 | Black Scottish or other Black | 1057 | 89.1 |
| 13 | Chinese | 15115 | 87.4 |
| 14 | Other ethnic group | 8945 | 86.2 |
|  |  |  |  |
|  | Total completing census | 4.86 million | 94.0 |
|  | Total population of Scotland (including estimated numbers) | 5.06 million | 91 |
|  |  |  |  |
|  | SHELS cohort in relation to those completing census | 4.62 million | 95.1 |
|  | SHELS cohort in relation to estimated Scottish population | 4.62 million | 91.3 |

**2 Socio-demographic characteristics of the study cohort**

Table B gives data on mean age at census, whether UK/RoI born or not, and Scottish Index of Multiple Deprivation (SIMD), highest qualification of individual (18-74 years) and for other age groups highest qualification in the household, and household tenure. Some description of this table is in the results section of the main text.

**Table B. Socio-demographic characteristics of linked population by sex and ethnic group**

| **Sex and ethnic group*** | **N** | **Age at Census in years** | | **Country**  **of Birth** | **SIMD** | | **Highest Qualification (individual)** | | | | **Highest Qualification (household)** | | | **Household Tenure** | |
| --- | --- | --- | --- | --- | --- | --- | --- | --- | --- | --- | --- | --- | --- | --- | --- |
|  |  | ***(mean, SD)*** | | ***UK/RoI born***  ***(%)*** | ***Most***  ***Deprived (%)*** | ***Least***  ***Deprived (%)*** | | ***None*** | ***Low*** | ***High*** | ***None*** | ***Low*** | ***High*** | ***Rented*** | ***Owned*** |
| **Male** |  |  | |  |  |  | |  |  |  |  |  |  |  |  |
| White Scottish | 1949484 | 38 | (22) | 99.1 | 20.1 | 19.8 | | 34.2 | 41.4 | 24.4 | 20.2 | 40.8 | 39.0 | 31.4 | 68.6 |
| Other White British | 160235 | 42 | (20) | 95.3 | 8.0 | 29.6 | | 17.2 | 34.5 | 48.4 | 10.0 | 28.1 | 61.9 | 27.7 | 72.3 |
| White Irish | 20341 | 45 | (20) | 98.4 | 22.2 | 21.7 | | 32.7 | 30.5 | 36.8 | 19.8 | 28.8 | 51.4 | 34.6 | 65.4 |
| Other White | 29944 | 36 | (21) | 30.5 | 12.4 | 32.6 | | 24.4 | 26.5 | 49.2 | 11.5 | 22.3 | 66.2 | 43.4 | 56.6 |
| Any Mixed Background | 5310 | 21 | (18) | 76.0 | 19.7 | 26.1 | | 21.1 | 42.1 | 36.8 | 12.0 | 28.3 | 59.7 | 42.9 | 57.1 |
| Indian | 6448 | 31 | (19) | 48.4 | 9.7 | 38.5 | | 21.5 | 27.6 | 50.9 | 10.0 | 25.5 | 64.5 | 27.8 | 72.2 |
| Pakistani | 12929 | 27 | (19) | 58.0 | 15.8 | 24.8 | | 39.7 | 33.9 | 26.5 | 17.3 | 35.2 | 47.5 | 23.8 | 76.2 |
| Other South Asian | 3549 | 29 | (19) | 38.9 | 24.2 | 28.3 | | 25.3 | 28.3 | 46.4 | 15.9 | 25.4 | 58.7 | 46.3 | 53.7 |
| African origin | 3277 | 30 | (18) | 39.9 | 27.6 | 22.4 | | 17.3 | 30.3 | 52.4 | 9.9 | 21.9 | 68.2 | 56.9 | 43.1 |
| Chinese | 6532 | 30 | (18) | 38.4 | 13.4 | 38.5 | | 39.0 | 29.2 | 31.8 | 21.2 | 30.5 | 48.3 | 30.2 | 69.8 |
|  |  |  |  |  |  |  | |  |  |  |  |  |  |  |  |
| **Female** |  |  |  |  |  |  | |  |  |  |  |  |  |  |  |
| White Scottish | 2138643 | 41 | (24) | 99.1 | 21.3 | 19.2 | | 35.7 | 40.6 | 23.8 | 21.9 | 40.3 | 37.8 | 34.8 | 65.2 |
| Other White British | 174748 | 44 | (21) | 95.0 | 8.2 | 28.9 | | 19.8 | 39.4 | 40.8 | 11.5 | 30.0 | 58.5 | 30.5 | 69.5 |
| White Irish | 23162 | 49 | (21) | 98.6 | 20.1 | 22.8 | | 32.9 | 29.1 | 38.0 | 20.8 | 25.8 | 53.4 | 35.9 | 64.1 |
| Other White | 35711 | 37 | (21) | 26.4 | 10.8 | 33.4 | | 21.5 | 27.2 | 51.3 | 10.7 | 20.7 | 68.7 | 41.3 | 58.7 |
| Any Mixed Background | 5799 | 24 | (20) | 74.5 | 18.7 | 27.3 | | 19.9 | 42.3 | 37.8 | 11.5 | 28.3 | 60.2 | 44.2 | 55.8 |
| Indian | 5888 | 30 | (19) | 51.1 | 9.6 | 39.0 | | 27.0 | 32.3 | 40.7 | 10.8 | 27.3 | 61.9 | 26.4 | 73.6 |
| Pakistani | 12702 | 26 | (18) | 60.5 | 15.4 | 24.6 | | 44.3 | 32.9 | 22.8 | 17.1 | 35.8 | 47.1 | 24.1 | 75.9 |
| Other South Asian | 2963 | 29 | (20) | 44.5 | 22.0 | 28.9 | | 29.5 | 33.7 | 36.7 | 18.0 | 26.9 | 55.2 | 45.5 | 54.5 |
| African origin | 3056 | 30 | (18) | 42.1 | 27.5 | 24.5 | | 17.8 | 36.1 | 46.1 | 10.2 | 22.2 | 67.6 | 54.9 | 45.1 |
| Chinese | 6672 | 31 | (18) | 33.9 | 12.1 | 39.1 | | 37.6 | 29.0 | 33.3 | 20.1 | 29.5 | 50.3 | 29.9 | 70.1 |

*As numbers were small some groups have been combined using the standard and published SHELS approach i.e. Bangladeshis have been combined with other South Asians and Black Scottish and other Black have been combined with African as an African Origin group.

**3. Additional details on calculating person-years and Poisson regression modelling**

To calculate person-years (PY) we used the linked census population where 1^st^ of May 2001 was the start of the period of PY calculation and 30^th^ of April 2013 the end (12 years of follow-up). The end of the period of PY calculation was adjusted by date of death or date of recall of notes from NHS Scotland to another part of the UK (as a marker of emigration) if they occurred prior to April 2013. Age standardised rates for ethnic minority groups by sex were derived by multiplying relative rate ratios adjusted for age and the White Scottish crude rates per 100,000 PY. We constructed Poisson models with robust variance, by sex and ethnic group firstly with age only (our primary analysis) and then included variables where we had a prior, specified hypothesis (secondary analysis); so there was no unspecified exploration (fishing), and no modelling with forward or backward selection. To examine interactions we included a new variable in the model combining ethnicity and UK/RoI born or not. In this analysis (main manuscript Fig 3 and Table G) all groups are compared to the White Scottish population born in the UK/RoI. Robust variance means the empirical (robust) estimator of the covariance matrix. It has the property of being a consistent estimator of the covariance matrix, even if the working correlation matrix is mis-specified [2-4]. We used SAS for our statistical analysis and the user documentation advises that if you include the statement ‘REPEATED SUBJECT=…/TYPE=unstr;’ that empirical (or robust) estimators are produced, even if you have only one observation per subject. The subject identifier is needed in the CLASS statement. We can supply the full computer code to interested readers.

**4. Strength of association between SES covariates and mortality**

The strength of association with the outcome for the three SES variables was examined in each ethnic group. With few exceptions, the strength of the associations between all-cause mortality and each SES variable was similar (overlapping confidence intervals) across the ethnic groups as shown in Table C. The percentages indicate the increased mortality associated with one level change in socio-economic position

**Table C: Strength of association (% change in relative rate ratio for each category change) between all-cause mortality with three SES indicators by ethnic group and sex, with 95% CIs**.

| **Ethnic group** | **Number of deaths** | **SIMD**  **(5 categories)** | **Highest qualification (combined household and individual into 3 categories)** | **Household tenure (2 categories)** |
| --- | --- | --- | --- | --- |
| **Male** |  |  |  |  |
| **White Scottish** | 251755 | 14.7 [11.2;18.1] | 23.4 [14.5;31.4] | 46.0 [38;53] |
| **Other White British** | 17855 | 15.2 [11.4;18.9] | 24.8 [20.3;29.1] | 41.2 [32.7;48.6] |
| **White Irish** | 3420 | 16.1 [11.9;20.1] | 21.6 [13.1;29.3] | 45.2 [34.5;54.2] |
| **Other White** | 2785 | 13.5 [9.5;17.3] | 18.1 [11.7;24.1] | 38.5 [26.1;48.8] |
| **Any Mixed Background** | 240 | 22.4 [16.4;28.1] | 33.4 [26.3;39.9] | 53.3 [39.3;64] |
| **Indian** | 280 | 18.1 [9;26.3] | 30.4 [15.7;42.6] | 51.4 [45.8;56.5] |
| **Pakistani** | 450 | 12.6 [4.9;19.7] | 9.7 [-10.4;26.1] | 40.5 [18.9;56.3] |
| **Other South Asian** | 150 | 16.1 [8.8;22.8] | 29.7 [19;39] | 47.5 [37.4;55.9] |
| **African origin** | 160 | 17.8 [8.9;25.8] | 23.0 [13.1;31.7] | 37.0 [18.6;51.2] |
| **Chinese** | 215 | 10.6 [2.4;18] | 20.9 [12;28.9] | 39.7 [22.8;52.8] |
|  |  |  |  |  |
| **No. of ethnic groups (of 10) where the association was positive, and,** |  | 10 | 10 | 10 |
|  |  |  |  |  |
| **95% CI excludes minus numbers** |  | 10 | 9 | 10 |

…cont’d

| **Female** |  |  |  |  |
| --- | --- | --- | --- | --- |
| **White Scottish** | 285440 | 10.6 [6.5;14.5] | 19.0 [8.3;28.4] | 42.8 [35.5;49.2] |
| **Other White British** | 19310 | 10.6 [6.7;14.3] | 21.1 [13.1;28.4] | 41.7 [34.4;48.2] |
| **White Irish** | 3895 | 7.7 [2.3;12.7] | 15.9 [7.4;23.6] | 37.1 [28;45.1] |
| **Other White** | 2670 | 11.7 [6.5;16.5] | 19.2 [11.9;25.8] | 45.4 [39.9;50.5] |
| **Any Mixed Background** | 230 | 16.0 [6.9;24.3] | 17.2 [-9.5;37.5] | 40.3 [31.1;48.3] |
| **Indian** | 175 | 14.9 [7.3;21.9] | 7 [-6.9;19] | 50.7 [45.3;55.5] |
| **Pakistani** | 295 | 7.4 [3.5;11] | 10.3 [-6;24.1] | 17.3 [0;31.7] |
| **Other South Asian** | 135 | 7.7 [-2.4;16.8] | 22.6 [10.5;33] | 32.6 [18.3;44.3] |
| **African origin** | 125 | 25.0 [18.6;30.9] | 20.1 [8;30.5] | 47.9 [36.3;57.4] |
| **Chinese** | 210 | 8.9 [0.7;16.3] | -0.5 [-15;11.9] | 32.2 [17.9;44.1] |
|  |  |  |  |  |
| **No. of ethnic groups (of 10) where the association was positive, and,** |  | 10 | 9 | 10 |
|  |  |  |  |  |
| **95% CI excludes minus numbers** |  | 9 | 6 | 10 |

*As numbers were small some groups have been combined using the standard and published SHELS approach i.e. Bangladeshis have been combined with other South Asians and Black Scottish and other Black have been combined with African as an African Origin group.

**5. Moving 3-year averages**

Table D shows 3-year moving average in mortality RRs. Some text on this table is in the results section of the paper.

**Table D. RRs adjusted by age by 3-year time period (moving averages) in mortality 2001-2013 by sex and ethnic group. (Each period starts in May and finishes in April.)**

|  | 2001-2004 | 2002-2005 | 2003-2006 | 2004-2007 | 2005-2008 | 2006-2009 | 2007-2010 | 2008-2011 | 2009-2012 | 2010-2013 |
| --- | --- | --- | --- | --- | --- | --- | --- | --- | --- | --- |
| Male |  |  |  |  |  |  |  |  |  |  |
| White Scottish | 100 | 100 | 100 | 100 | 100 | 100 | 100 | 100 | 100 | 100 |
| Other White British | 72.9 | 74.0 | 76.3 | 75.6 | 76.1 | 77.9 | 79.2 | 79.5 | 77.0 | 76.8 |
| White Irish | 104.9 | 99.7 | 102 | 101.7 | 105.4 | 104.9 | 100.6 | 96.5 | 97.8 | 99.3 |
| Other White | 88.0 | 84.8 | 85.3 | 83.2 | 81.4 | 82.8 | 85.3 | 89.6 | 85.2 | 82.2 |
| Any Mixed Background | 118.0 | 123.3 | 129.7 | 128.6 | 133.0 | 117.4 | 128.1 | 133.4 | 118.3 | 90.0 |
| Indian | 55.0 | 61.0 | 60.5 | 68.3 | 66.0 | 66.1 | 75.6 | 65.5 | 58.8 | 50.0 |
| Pakistani | 65.1 | 70.4 | 61.4 | 64.8 | 61.5 | 62.0 | 65.5 | 66.5 | 74.0 | 67.0 |
| Bangladeshi | . | . | 54.5 | . | 51.9 | 69.4 | 89.0 | 72.6 | 63.2 | . |
| Other South Asian | 95.7 | 113.6 | 123.4 | 102.9 | 98.8 | 97.4 | 99.6 | 76.5 | 56.2 | 68.4 |
| Caribbean | 64.8 | 57.2 | 77.0 | 70.0 | 108.2 | 104.2 | 134.7 | 119.4 | 128.4 | 109.0 |
| African | 102.1 | 88.0 | 82.3 | 92.0 | 89.9 | 71.3 | 70.9 | 59.9 | 80.5 | 88.6 |
| Black Scottish or Other Black | 100.6 | 131.8 | 159.2 | 125.2 | 96.7 | 87.9 | 101.2 | 114.9 | 96.4 | 87.7 |
| Chinese | 61.0 | 57.7 | 50.2 | 44.3 | 41.7 | 42.4 | 43.6 | 45.8 | 54.2 | 66.0 |
|  |  |  |  |  |  |  |  |  |  |  |
| Female |  |  |  |  |  |  |  |  |  |  |
| White Scottish | 100 | 100 | 100 | 100 | 100 | 100 | 100 | 100 | 100 | 100 |
| Other White British | 78.5 | 78.5 | 79.0 | 80.2 | 79.1 | 79.4 | 79.3 | 80.5 | 82.3 | 82.6 |
| White Irish | 89.5 | 90.6 | 91.9 | 93.6 | 96.2 | 95.4 | 94.2 | 90.7 | 90.1 | 88.5 |
| Other White | 82.9 | 78.7 | 77.1 | 79.1 | 81.3 | 83.2 | 81.0 | 77.3 | 77.9 | 74.8 |
| Any Mixed Background | 98.7 | 76.8 | 85.8 | 85.8 | 106.5 | 114.4 | 102.9 | 85.2 | 75.9 | 83.7 |
| Indian | 66.1 | 54.9 | 50.0 | 51.7 | 59.1 | 66.3 | 59.5 | 59.1 | 60.3 | 76.9 |
| Pakistani | 61.1 | 62.7 | 61.7 | 74.4 | 81.1 | 78.4 | 75.0 | 73.0 | 78.5 | 82.6 |
| Bangladeshi | . | . | 115.9 | 142.7 | . | . | . | . | . | . |
| Other South Asian | 96.7 | 129.0 | 120.8 | 122.4 | 107.6 | 111.6 | 112.2 | 113.7 | 114.2 | 101.1 |
| Caribbean | 66.8 | 63.8 | 63.4 | 62.6 | 100.7 | 91.8 | 108.5 | 62.3 | 61.4 | 51.5 |
| African | 125.2 | 182.1 | 166.6 | 150.2 | 88.3 | 85.1 | 91.3 | 86.7 | 66.2 | 69.8 |
| Black Scottish or Other Black | 155.5 | 156.9 | 166.4 | 92.5 | 81.3 | 68.2 | 66.6 | 76.1 | 108.6 | 130.7 |
| Chinese | 65.8 | 70.3 | 66.1 | 65.6 | 68.7 | 67.5 | 73.6 | 65.9 | 63.4 | 60.41 |

**6 Data underlying main manuscript Fig 3 (males) and 4 (females) on the primary care subcohort**

**Table E. Mortality between 2001 and 2013 in four ethnic groups in a general practice record linked sub-cohort providing data on smoking and diabetes mellitus. Rates are for 100,000 PY and age-adjusted, and RRs are adjusted for age, CoB, Scottish Index of Multiple Deprivation (SIMD), education, household tenure (3 SES variables combined) and smoking and diabetes with 95% CIs)**

|  | | | | **RRs and adjustment variables** | | | | |
| --- | --- | --- | --- | --- | --- | --- | --- | --- |
| **Ethnic group** | **No. of deaths** | **PY at risk** | **Rates** | **Age** | **Age, CoB, and 3 SES variables** | **Age, CoB, 3 SES**  **variables and smoking** | **Age, CoB, 3 SES**  **variables and diabetes** | **Age, CoB, 3 SES**  **variables, smoking and diabetes** |
| **Males**  White Scottish | 1080 | 162300 | 664.8 | 100.00 | 100.0 | 100.0 | 100.0 | 100.0 |
| Other White British | 55 | 14540 | 683.0 | 74.1 (55.9, 98.2) | 103.0 (76.4, 138.8) | 102.7 (76.0, 138.9) | 103.2 (77.3, 137.8) | 103.1 (77.0,137.9) |
| Indian | 30 | 5370 | 829.6 | 96.9 (67.9, 138.3) | 117.2 (80.7, 170.0) | 124.8 (86.2, 180.7) | 108.2 (73.9, 158.5) | 114.7 (78.3, 167.9) |
| Pakistani | 75 | 23810 | 520.0 | 69.7 (54.8, 88.6) | 77.1 (57.5, 103.3) | 78.2 (58.3, 104.8) | 70.7 (52.4, 95.3) | 72.0 (53.4, 97.0) |
| **Female**  White Scottish | 980 | 191605 | 511.5 | 100.00 | 100.0 | 100.0 | 100.0 | 100.0 |
| Other White British | 55 | 17530 | 510.8 | 75.3 (58.1, 97.5) | 98.5 (75.1, 129.1) | 99.9 (76.5, 130.4) | 100.4(76.7, 131.5) | 101.7 (77.9, 132.8) |
| Pakistani | 55 | 23680 | 599.3 | 86.8 (63.8, 118.0) | 103.9 (73.7, 146.6) | 117.2 (83.2, 165.1) | 92.5 (66.0, 129.8) | 103.9 (73.9, 145.9) |

*The number of deaths in Indian women was rounded to 10 with wide 95% CIs making interpretation difficult so the data line has been removed.

**7. Data underlying main manuscript Fig 5 (males) and 6 (females) on mortality by whether UK/RoI born or not**

**Table F. Age-adjusted mortality rates per 100000 PY and RRs, by sex, ethnic group and UK/RoI-born or not.**

**Male**

| **Born in/outside UK/RoI and ethnic group** | **Number of deaths** | **PY** | **Rates (per 100,000 )** | **RR** | **lower CI** | **upper CI** |
| --- | --- | --- | --- | --- | --- | --- |
| In UK/RoI White Scottish | 199245 | 20989260 | 949.3 | 100.0 | . | . |
| Other White British | 12410 | 1498905 | 683.8 | 72.0 | 63.8 | 81.3 |
| White Irish | 2685 | 199170 | 950.4 | 100.1 | 88.3 | 113.5 |
| Other White | 530 | 96830 | 827.7 | 87.2 | 75.2 | 101.1 |
| Any Mixed Background | 160 | 43885 | 1270.6 | 133.9 | 112.7 | 158.9 |
| Indian | 40 | 33800 | 722.9 | 76.2 | 53.2 | 108.9 |
| Pakistani | 60 | 86570 | 600.5 | 63.3 | 46.6 | 85.8 |
| Bangladeshi | 10 | 3855 | 1098.0 | 115.7 | 56.0 | 239.1 |
| Other South Asian | 45 | 10775 | 1284.1 | 135.3 | 99.8 | 183.4 |
| Caribbean | 15 | 4455 | 1111.5 | 117.1 | 74.3 | 184.5 |
| African | 20 | 5540 | 1583.6 | 166.8 | 106.3 | 261.9 |
| Black Scottish or Other Black | 15 | 3535 | 867.4 | 91.4 | 59.0 | 141.6 |
| Chinese | 25 | 28385 | 615.8 | 64.9 | 43.4 | 96.9 |

| Outside UK/RoI White Scottish | 1485 | 190495 | 760.9 | 80.2 | 71.4 | 90.0 |
| --- | --- | --- | --- | --- | --- | --- |
| Other White British | 565 | 72175 | 704.7 | 74.2 | 64.8 | 85.1 |
| White Irish | 25 | 3020 | 633.4 | 66.7 | 47.8 | 93.1 |
| Other White | 1400 | 181680 | 744.5 | 78.4 | 69.8 | 88.1 |
| Any Mixed Background | 35 | 12380 | 592.9 | 62.5 | 41.4 | 94.3 |
| Indian | 225 | 32145 | 575.3 | 60.6 | 49.1 | 74.8 |
| Pakistani | 375 | 59855 | 630.8 | 66.5 | 57.0 | 77.5 |
| Bangladeshi | 10 | 4845 | 286.6 | 30.2 | 16.6 | 54.8 |
| Other South Asian | 70 | 16025 | 693.6 | 73.1 | 56.2 | 95.1 |
| Caribbean | 30 | 2790 | 815.3 | 85.9 | 64.7 | 113.9 |
| African | 40 | 14595 | 647.0 | 68.2 | 46.1 | 100.8 |
| Black Scottish or Other Black | 10 | 1245 | 844.3 | 88.9 | 44.9 | 176.3 |
| Chinese | 165 | 40300 | 479.6 | 50.5 | 41.6 | 61.3 |

**Female**

| **Born in/outside UK/RoI and ethnic group** | **Number of deaths** | **PY** | **Rates (per 100,000 )** | **RR** | **lower CI** | **upper CI** |
| --- | --- | --- | --- | --- | --- | --- |
| In UK/RoI White Scottish | 178625 | 22373250 | 798.4 | 100.0 | . | . |
| Other White British | 10660 | 1563770 | 601.0 | 75.3 | 67.9 | 83.5 |
| White Irish | 2460 | 213955 | 698.5 | 87.5 | 79.0 | 96.9 |
| Other White | 435 | 97450 | 719.9 | 90.2 | 77.3 | 105.2 |
| Any Mixed Background | 115 | 46080 | 824.0 | 103.2 | 84.6 | 125.9 |
| Indian | 30 | 31735 | 702.7 | 88.0 | 59.3 | 130.7 |
| Pakistani | 50 | 87785 | 681.7 | 85.4 | 62.4 | 116.8 |
| Bangladeshi | 10 | 3255 | 1254.0 | 157.1 | 88.7 | 278.1 |
| Other South Asian | 45 | 10205 | 1141.3 | 143.0 | 105.0 | 194.7 |
| Caribbean | 20 | 4700 | 935.9 | 117.2 | 76.5 | 179.6 |
| African | 15 | 4765 | 964.2 | 120.8 | 68.2 | 213.9 |
| Black Scottish or Other Black | 20 | 3530 | 863.3 | 108.1 | 69.5 | 168.3 |
| Chinese | 25 | 25195 | 898.9 | 112.6 | 74.8 | 169.4 |
|  |  |  |  |  |  |  |
| Outside UK/RoI White Scottish | 1330 | 207940 | 637.4 | 79.8 | 71.5 | 89.1 |
| Other White British | 495 | 80665 | 568.1 | 71.2 | 62.8 | 80.6 |
| White Irish | 10 | 2950 | 536.2 | 67.2 | 39.3 | 114.7 |
| Other White | 1295 | 222465 | 576.9 | 72.3 | 64.4 | 81.1 |
| Any Mixed Background | 25 | 13890 | 441.4 | 55.3 | 38.0 | 80.5 |
| Indian | 120 | 28190 | 446.8 | 56.0 | 45.6 | 68.6 |
| Pakistani | 245 | 56155 | 572.5 | 71.7 | 61.1 | 84.2 |
| Bangladeshi | 10 | 3655 | 313.5 | 39.3 | 17.8 | 86.7 |
| Other South Asian | 35 | 11500 | 562.3 | 70.4 | 51.6 | 96.2 |
| Caribbean | 10 | 3125 | 181.2 | 22.7 | 10.7 | 48.3 |
| African | 30 | 11100 | 718.6 | 90.0 | 60.2 | 134.5 |
| Black Scottish or Other Black | . | . | . | . | . | . |
| Chinese | 150 | 42810 | 492.8 | 61.7 | 51.3 | 74.2 |

**8 Possible implications of choosing a cut-off other than 10% to signify public health importance : Table prepared in in response to referee 3’s comments on sensitivity analysis.**

Our focus in the paper is on differences in age-adjusted rate ratios (RRs) compared with the White Scottish reference that are greater than 10% i.e. outside the range 90-110. A referee recommended a sensitivity analysis to see if the interpretation would change if we had chosen differences of greater than 5%, 15% or 20%. In Table G1 and G2, we use the age-adjusted RRs by sex. We then use the range of the 95% CIs and the central estimates to see the effect of different cut-offs.  If the limit of the 95% CI is outside the cut-off, we can have at least 95% confidence that such a difference is real, i.e. “strong” evidence. If the central estimate is outside the cut-off, but the 95% CI overlaps it, we consider that “weak” evidence of a real difference. For example, for Other White British females, the RR is 75.2 and the 95% CI 68.0-83.2.  As the upper limit of the 95% CI is 83.2, we have strong evidence that the difference is more than 15%.  As the central estimate is 75.2 we have weak evidence that the difference is more than 20%.

Table G shows that among males, there was strong evidence that the real difference was more than 10% for six ethnic groups, more than 15% for five and more than 20% for four, with weak evidence it was more than 20% for a further one group. Among females, there was strong evidence the real difference was more than 10% for six ethnic groups, more than 15% for four and more than 20% for two, with weak evidence that the difference was more than 20% for a further five groups. Thus, choosing different cut-offs did alter the number of ethnic groups for which there was strong evidence their RR was lower than the cut-off. Only one group, males of Mixed Background, had a 95% CI lower limit that was more than 10% above the reference and all but one male and three female groups had central estimates that were at least 5% lower. Thus varying the cut-off did not alter the general conclusion that mortality rates among most ethnic groups are lower than those of the White Scottish reference, and in some groups considerably so and therefore of importance for population health. For some of the smaller ethnic groups, the small number of deaths during the follow-up period meant that the 95% CIs were wide, thereby weakening the evidence.

**Table G1. The size of differences in age standardised rate ratios for males in ethnic groups compared to the White Scottish reference, using 95% CI limits or central estimates.**

| **Difference in RR relative to White Scottish** | **Strong evidence of difference**  **(based on 95% CI limits )** | **Weak evidence of difference**  **(based on central estimate regardless of 95% CI )** |
| --- | --- | --- |
| >20% higher |  |  |
| 15-20% higher |  |  |
| 10-15%% higher |  | Mixed Background |
| 5-10% higher |  |  |
| 0-5% % higher |  |  |
| 0-5% lower |  | White Irish |
| 5-10% lower |  | Caribbean  Black Scottish or Other Black |
| 10-15% lower | Other White | Other South Asian |
| 15-20% lower | Other White British | Other White  African |
| >20% lower | Indian  Pakistani  Bangladeshi  Chinese | Other White British |

**Table G. The size of differences in age standardised rate ratios for females in ethnic groups compared to the White Scottish reference, using 95% CI limits or central estimates.**

| **Difference in RR relative to White Scottish** | **Strong evidence**  **(based on 95% CI limits )** | **Weak evidence**  **(based on central estimate regardless of 95% CI)** |
| --- | --- | --- |
| >20% higher |  |  |
| 15-20% higher |  |  |
| 10-15% higher |  |  |
| 5-10% higher |  |  |
| 0-5 % higher |  |  |
| 0-5% lower | White Irish | Other South Asian  African |
| 5-10% lower |  | Black Scottish or Other Black |
| 10-15% lower | Pakistani  Caribbean | White Irish  Mixed Background |
| 15-20% lower | Other White British  Other White |  |
| >20% lower | Indian  Chinese | Other White British  Other White  Bangladeshi  Pakistani  Caribbean |

**9 Examination of country of birth analysis with particular reference to Fishbacher et al’s earlier findings.**

**Table H. Comparison of published standardised mortality ratios (SMRs) by sex and CoB, 1997-2003, with Scotland born as reference [5], and current age-adjusted mortality rate ratios (RRs), 2001-2013, by sex and CoB or ethnic group with White Scottish as reference.**

| **CoB**  **group*** | **SMR**** | **This study using CoB** | | **RR** | **This study using equivalent ethnic group** | **RR** |
| --- | --- | --- | --- | --- | --- | --- |
| **A - Male** |  |  |  | |  |  |
| Scotland | 100 | Scotland | | 100 | White Scottish | 100 |
| Other UK | 75.4 | Other UK | | 76.3 | Other White British | 72.3 |
| N. Ireland | 100.3 | N. Ireland | | 93.6 | White Irish | 99.9 |
| R. of Ireland | 108.2 | R. of Ireland | | 102.8 |  |  |
| India | 88.4 | India | | 70.0 | Indian | 62.6 |
| Pakistan | 62.9 | Pakistan | | 64.6 | Pakistani | 66.1 |
| Bangladesh | 43.6 | Bangladesh | | 39.9 | Bangladeshi | 50.7 |
| China | 72.0 | China | | 64.3 | Chinese | 52.2 |
| Hong Kong | 69.3 | Hong Kong | | 55.3 |  |  |
|  |  |  | |  |  |  |
| **B – Female** |  |  | |  |  |  |
| Scotland | 100 | Scotland | | 100 | White Scottish | 100 |
| Other UK | 82.0 | Other UK | | 80.0 | Other White British | 75.2 |
| N. Ireland | 93.5 | N. Ireland | | 90.6 | White Irish | 87.5 |
| R. of Ireland | 101.7 | R. of Ireland | | 89.0 |  |  |
| India | 97.2 | India | | 74.2 | Indian | 60.7 |
| Pakistan | 70.7 | Pakistan | | 72.4 | Pakistani | 73.8 |
| Bangladesh | 44.1 | Bangladesh | | 40.7 | Bangladeshi | 66.0 |
| China | 74.5 | China | | 70.3 | Chinese | 65.8 |
| Hong Kong | 86.1 | Hong Kong | | 62.2 |  |  |

Note:

*No data were published by Fischbacher et al on Africa or Caribbean born population; and a comparison with any Mixed Background group and disaggregating the two parts of Ireland and between China and Hong Kong is not possible by ethnicity.

** Fischbacher et al analysed data for the age group 25 years or more. For confidence intervals around estimates see cited articles and as given in text

Reference List

(1) Fischbacher CM, Bhopal R, Povey C, et al. (2007) Record linked retrospective cohort study of 4.6 million people exploring ethnic variations in disease: myocardial infarction in South Asians. BMC Public Health 7(1): 142.

(2) White H (2009) Maximum Likelihood Estimation of Misspecified Models. Econometrica 50: 1-25.

(3) Royall RM (1986) Model Robust Confidence Intervals Using Maximum Likelihood Estimators. International Statistical Review 54(2): 221-226.

(4) Zeger SL, Liang KY, Albert PS (1988) Models for longitudinal data: a generalized estimating equation approach. Biometrics 44(4): 1049-1060.

(5) Wild S, McKeigue P. Cross sectional analysis of mortality by country of birth in England and Wales, 1970-92. BMJ. 1997;314(7082):705-10.
